# PRMT inhibitor promotes *SMN2* exon 7 inclusion and synergizes with nusinersen to rescue SMA mice

**DOI:** 10.15252/emmm.202317683

**Published:** 2023-09-19

**Authors:** Anna J Kordala, Jessica Stoodley, Nina Ahlskog, Muhammad Hanifi, Antonio Garcia Guerra, Amarjit Bhomra, Wooi Fang Lim, Lyndsay M Murray, Kevin Talbot, Suzan M Hammond, Matthew JA Wood, Carlo Rinaldi

**Affiliations:** ^1^ Department of Physiology Anatomy and Genetics University of Oxford Oxford UK; ^2^ Department of Paediatrics University of Oxford Oxford UK; ^3^ Institute of Developmental and Regenerative Medicine (IDRM) Oxford UK; ^4^ Centre for Discovery Brain Sciences, College of Medicine and Veterinary Medicine University of Edinburgh Edinburgh UK; ^5^ Euan McDonald Centre for Motor Neuron Disease Research University of Edinburgh Edinburgh UK; ^6^ Nuffield Department of Clinical Neurosciences, John Radcliffe Hospital University of Oxford Oxford UK; ^7^ Kavli Institute for Nanoscience Discovery University of Oxford Oxford UK; ^8^ MDUK Oxford Neuromuscular Centre Oxford UK

**Keywords:** nusinersen, PRMT inhibitor, small molecule, spinal muscular atrophy, Musculoskeletal System, Neuroscience, Pharmacology & Drug Discovery

## Abstract

Spinal muscular atrophy (SMA) is a leading genetic cause of infant mortality. The advent of approved treatments for this devastating condition has significantly changed SMA patients' life expectancy and quality of life. Nevertheless, these are not without limitations, and research efforts are underway to develop new approaches for improved and long‐lasting benefits for patients. Protein arginine methyltransferases (PRMTs) are emerging as druggable epigenetic targets, with several small‐molecule PRMT inhibitors already in clinical trials. From a screen of epigenetic molecules, we have identified MS023, a potent and selective type I PRMT inhibitor able to promote *SMN2* exon 7 inclusion in preclinical SMA models. Treatment of SMA mice with MS023 results in amelioration of the disease phenotype, with strong synergistic amplification of the positive effect when delivered in combination with the antisense oligonucleotide nusinersen. Moreover, transcriptomic analysis revealed that MS023 treatment has minimal off‐target effects, and the added benefit is mainly due to targeting neuroinflammation. Our study warrants further clinical investigation of PRMT inhibition both as a stand‐alone and add‐on therapy for SMA.

## Graphical abstract

Spinal muscular atrophy (SMA) is a leading genetic cause of infant mortality. To address the urgent need to develop new approaches for improved and long‐lasting benefits for SMA patients, an orally available, epigenetic‐targeting small molecule was identified and tested in SMA models alone and in combination with a recently approved treatment.

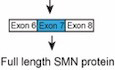

**The paper explained**

**Problem**

Spinal muscular atrophy (SMA) is a neuromuscular disorder resulting from inactivating mutations in the survival motor neuron 1 (*SMN1*) gene, making it a prominent genetic contributor to infant mortality on a global scale. Over the past 10 years, the emergence of effective treatments has notably decelerated the progression of the disease and enhanced well‐being of individuals affected by SMA. However, these treatments have several disadvantages, including substantial costs and side effects associated with long‐term use. Consequently, there is a pressing need for novel therapeutic approaches, whether used independently or in conjunction with existing ones.

**Results**

From a screen of potent and highly selective small molecules, a protein methyltransferases (PRMTs) inhibitor, MS023, was identified, capable of elevating SMN protein levels in SMA models and to synergistically amplify the effects of nusinersen, a clinically approved antisense oligonucleotide (ASO) for SMA patients.

**Impact**

These results highlight a link between protein arginine methylation and SMN regulation. Furthermore, these data provide proof of concept evidence for the use of MS023, a potent and selective PRMTs inhibitor, both as a stand‐alone and an add‐on treatment for SMA patients.

## Introduction

Spinal muscular atrophy (SMA) is a genetic neuromuscular condition affecting 1:8,000–10,000 live births (Pearn, 1978) and, to this date, a leading inherited cause of infant mortality worldwide (Crawford & Pardo, 1996). SMA is caused by inactivating mutations, mainly homozygous deletions, in the survival motor neuron 1 (*SMN1*) gene on chromosome 5 (Lefebvre *et al*, 1995). The encoded SMN protein is ubiquitously expressed, localising both to the cytoplasmic and nuclear compartments within the cell, where it exerts numerous essential functions, including biogenesis of small nuclear ribonucleoproteins (snRNPs), its most widely studied function (Friesen *et al*, 2001a; Pellizzoni *et al*, 2002; Zhang *et al*, 2011), 3′ processing of histone mRNAs (Tisdale *et al*, 2013), control of transcription (Strasswimmer *et al*, 1999;Suraweera *et al*, 2009; Yanling Zhao *et al*, 2016), R‐loop resolution (Suraweera *et al*, 2009; Yanling Zhao *et al*, 2016), RNA trafficking (Rossoll *et al*, 2003; Piazzon *et al*, 2008; Tadesse *et al*, 2008; Akten *et al*, 2011; Fallini *et al*, 2011, 2014, 2016; Hubers *et al*, 2011; Rage *et al*, 2013) and pre‐mRNA splicing (Pellizzoni *et al*, 1998; Charroux *et al*, 1999; Shafey *et al*, 2010; Makarov *et al*, 2012). The *SMN2* gene is a centromeric copy of telomeric *SMN1*, with a critical C to T substitution in position 6 of exon 7, which creates an exonic splicing silencer (ESS) and is recognised by a splicing factor, HNRNPA1 (Kashima & Manley, 2003; Kashima *et al*, 2007a). As a consequence, the *SMN2* gene mainly encodes a shorter and rapidly degraded SMN isoform lacking exon 7 (Δ7 SMN), with only 10–15% of *SMN2* transcripts still capable of generating a full‐length SMN protein (Lorson *et al*, 1999; Monani *et al*, 1999). The number of *SMN2* copies varies in the general population and is the main modifier of disease severity identified so far, with a higher number of copies being associated with a milder SMA phenotype (McAndrew *et al*, 1997; Feldkötter *et al*, 2002). Depending on the age of onset and motor milestones achieved, SMA has been divided into four clinical types (I–IV) (Munsat & Davies, 1992), with type I SMA infants showing symptoms before 6 months of age and never gaining the ability to sit unaided.

Loss of SMN leads to degeneration of lower α‐motor neurons by molecular mechanisms, which are not fully understood. Other neuronal and non‐neuronal cell populations are affected in SMA patients and include sensory neurons (Rudnik‐Schöneborn *et al*, 2003; Jablonka *et al*, 2006; Mentis *et al*, 2011; Gogliotti *et al*, 2012; Martinez *et al*, 2012), skeletal muscle (Arnold *et al*, 2004; Martínez‐Hernández *et al*, 2009; Kim *et al*, 2020) heart and vasculature (Araujo *et al*, 2009; Bevan *et al*, 2010; Heier *et al*, 2010; Rudnik‐Schöneborn *et al*, 2010; Somers *et al*, 2016; Lipnick *et al*, 2019), liver (Crawford *et al*, 1999; Deguise *et al*, 2019), and pancreas (Bowerman *et al*, 2012), supporting the notion that SMA is a multisystemic condition.

In the last decade, the advent of successful therapeutic approaches, combined with improvements in standards of care, has effectively changed the course of this disease, significantly slowing down the progression of all SMA types (Harding *et al*, 2015; Mercuri *et al*, 2020, 2022). One of such approved strategies entails intrathecal injections of nusinersen, an antisense oligonucleotide (ASO) targeting intronic splicing silencer N1 (ISS‐N1), promoting *SMN2* exon 7 inclusion and increasing levels of full‐length SMN protein. Concomitantly, these treatment opportunities pose new challenges, including their yet‐to‐be determined long‐term effects, the rise of new phenotypes in treated patients, which is particularly relevant for approaches such as nusinersen, solely targeting the central nervous system (CNS), the need for repeated invasive administrations, and high costs. Altogether, these considerations highlight the urgency for development of therapeutic combinations to address these important limitations and to provide additional benefit to patients.

Epigenetic regulation of gene expression, which involves covalent and sequence‐specific modifications of histone and non‐histone proteins, is a dynamic and reversible process that establishes normal cellular phenotypes and, when dysregulated, contributes to a wide range of human diseases, including SMA (Allis *et al*, 2007; Hauke *et al*, 2009; Portela & Esteller, 2010; Zheleznyakova *et al*, 2013; Murray *et al*, 2015; Cao *et al*, 2016). In recent years, key protein families that mediate epigenetic signalling through the acetylation and methylation of histones and non‐histone proteins, including histone deacetylases (HDACs), protein methyltransferases (PRMTs), histone lysine methyltransferases (KMTs) and demethylases (KDMs), and bromodomain‐containing proteins (BRD) have emerged as attractive druggable targets using small molecules, due to the dynamic nature of disease‐associated epigenetic states (Arrowsmith *et al*, 2012). Several small‐molecule inhibitors of histone deacetylases have been tested in SMA models (Mohseni *et al*, 2013), but their lack of specificity, low potency, and poor understanding of their mechanisms of action have significantly limited their translation into the clinic (Mercuri *et al*, 2007; Kissel *et al*, 2014; Krosschell *et al*, 2018). A recent study has shown that nusinersen, while promoting exon 7 inclusion, also induces a silencing histone mark H3K9me2 on *SMN2* gene, creating a roadblock to RNA polymerase II elongation. Histone deacetylase inhibitor—valproic acid, counteracts chromatin effects of the ASO, resulting in higher exon 7 inclusion upon combined treatment compared to nusinersen alone (Marasco *et al*, 2022). The primary aim of this project is to identify next generation small molecules targeting epigenetic proteins able to increase SMN protein and evaluate their therapeutic potential in SMA animal models alone and as an add‐on treatment.

## Results

### Epigenetic screening of *SMN2* modulators

**Figure 1:**
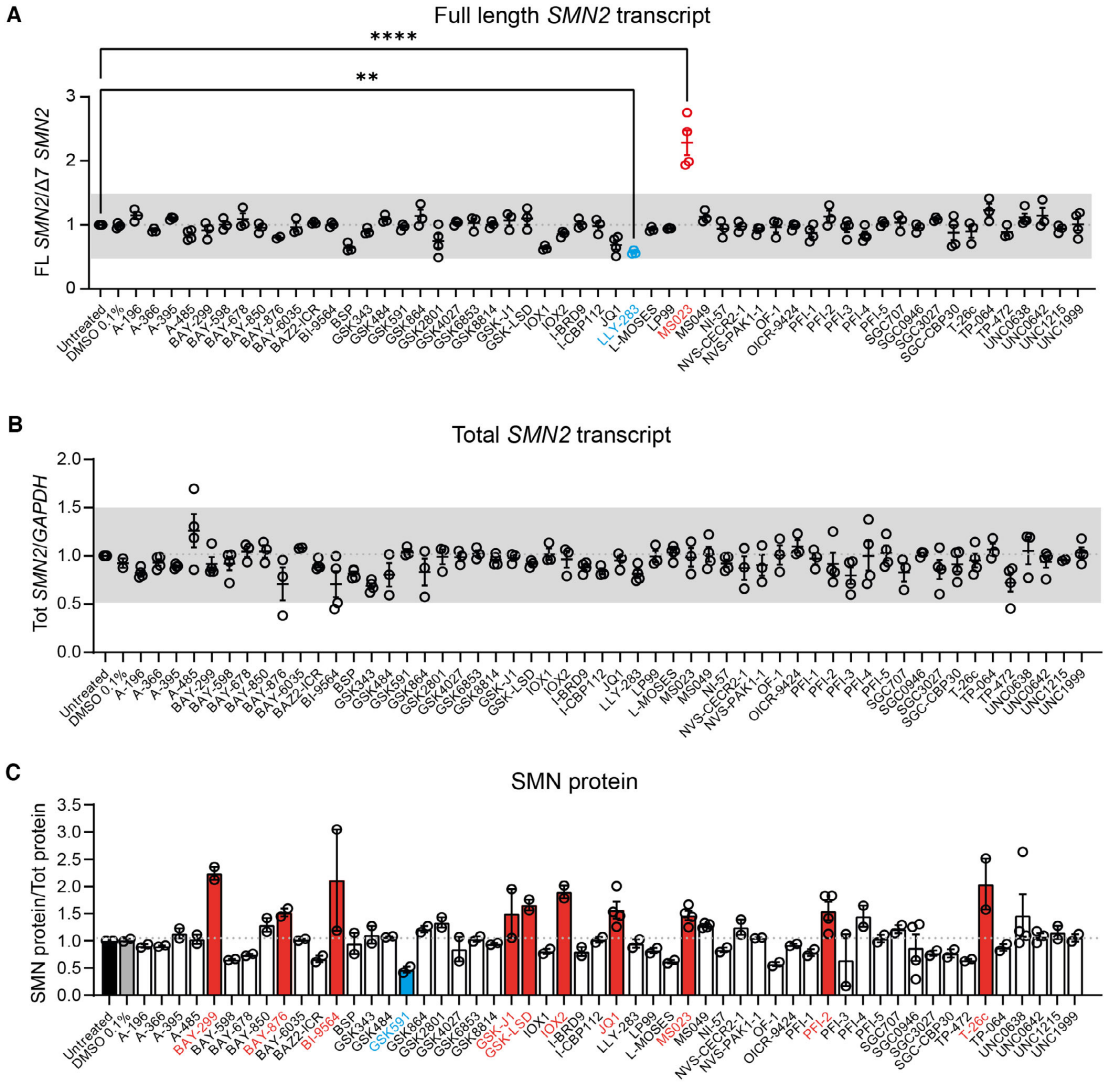
Screening of epigenetic small molecules in SMA fibroblasts A–CSMA type II patient‐derived fibroblasts were treated with the indicated small molecule at the appropriate maximum tolerated dose (range: 1–10 μM) (*n* = 3–4). Cells were harvested for RNA (A, B) and protein (C) quantification 48 and 72 h post‐treatment, respectively. (A) Full‐length (FL) *SMN2* transcript levels relative to Δ7 *SMN*2 are expressed as fold change compared to untreated SMA fibroblasts, normalised to one (dashed line). FL *SMN2*/Δ7 *SMN2* ratios were significantly increased by MS023 and decreased by LLY‐283 treatment. (B) Tot *SMN2* transcript levels relative to *GAPDH* are expressed as fold change compared to untreated SMA fibroblasts, normalised to one (dashed line). (A, B) Each dot represents a biological replicate (*n* = 3–4). The grey bar indicates values within the 0.5–1.5 range. (C) SMN protein levels relative to total protein are expressed as fold change compared to untreated SMA fibroblasts, normalised to one (dashed line). Each dot represents a biological replicate (*n* = 2–4). Values ≥ 1.5 and ≤ 0.5 are depicted in red and blue, respectively. (A–C) Data are represented as mean ± standard error of the mean (s.e.m.) and compared with a one‐way ANOVA test with multiple comparisons (**P ≤ 0.01; ****P ≤ 0.0001). Source data are available online for this figure.

**Figure EV1:**
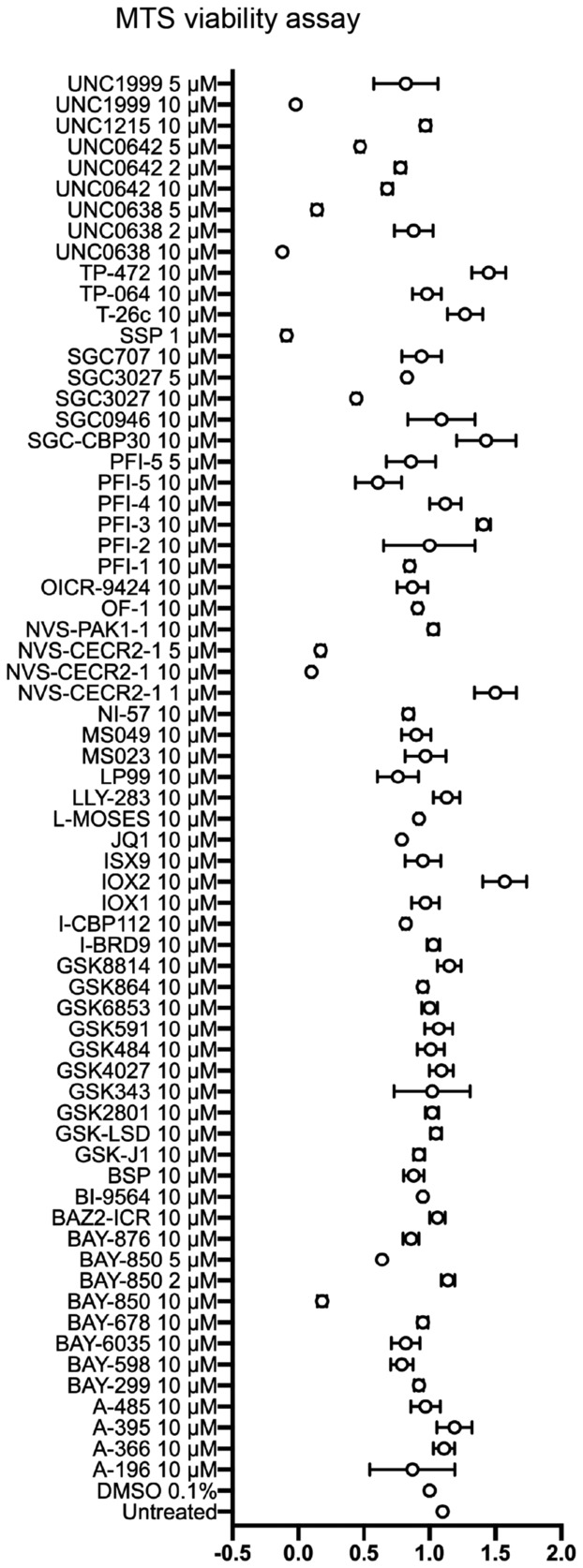
Effect of epigenetic small molecules on SMA type II patient‐derived fibroblast viability Viability of cells, assayed by MTS assay, treated with epigenetic small molecules, relative to vehicle‐treated cells (0.1% DMSO), normalised to one (*n* = 2–3). Data are represented as mean ± s.e.m.

In order to identify small molecules that selectively modulate *SMN2* pre‐mRNA splicing to include exon 7, we performed a cell‐based screen in SMA type II‐patient derived fibroblasts carrying three copies of the *SMN2* gene, using a collection of 54 chemical probes from the Structural Genomics Consortium (SGC) collection (https://www.thesgc.org/chemical‐probes) (Scheer *et al*, 2019; Wu *et al*, 2019) (Table EV1). This unique library includes compounds targeting key epigenetic regulatory proteins with a high degree of potency and selectivity, and a favourable therapeutic index (Ackloo *et al*, 2017). The maximum non‐toxic concentrations for each compound, established by a viability assay in these cells, were used in the screen (Fig EV1). Of the 54 molecules, only one molecule, selective type I PRMT inhibitor MS023, was able to promote exon 7 inclusion in *SMN2* pre‐mRNA, without affecting total *SMN2* mRNA levels (Fig 1A and B). MS023 treatment in these fibroblasts also increased SMN protein levels up to 1.6‐fold, as determined by Western blot analysis (Fig 1C). Notably, treatment with PRMT5 inhibitors: LLY‐283 and GSK591 resulted in reduction of exon 7 inclusion in *SMN2* pre‐mRNA and in SMN protein, respectively (Fig 1A–C), overall suggesting that protein arginine asymmetric and symmetric dimethylation by different families of PRMTs exert opposite effects on SMN regulation. Other compounds, including bromodomain inhibitors (BAY‐299, BI‐9564, JQ1) and lysine demethylase inhibitors (GSK‐J1, GSK‐LSD), also elicited a ≥ 1.5‐fold increase in SMN protein without affecting mRNA levels, hinting at a direct or indirect effect on SMN protein regulation (Fig 1A–C). PRMTs are involved in several critical biological functions (Blanc & Richard, 2017), and represent a promising therapeutic target for many human diseases from cancer to neurodegeneration, with at least eight PRMT inhibitors attaining clinical trial testing in human cancers (Yang & Bedford, 2013; Guccione & Richard, 2019; Hwang *et al*, 2021). Altogether, the direct effect of PRMT type I inhibition on *SMN2* exon 7 inclusion and the potential for clinical impact of this class of molecules, prompted us to further investigate MS023 as a therapeutic agent for SMA.

### Type I PRMT inhibition promotes exon 7 inclusion in SMN2 pre‐mRNA by decreasing HNRNPA1 binding

**Figure 2:**
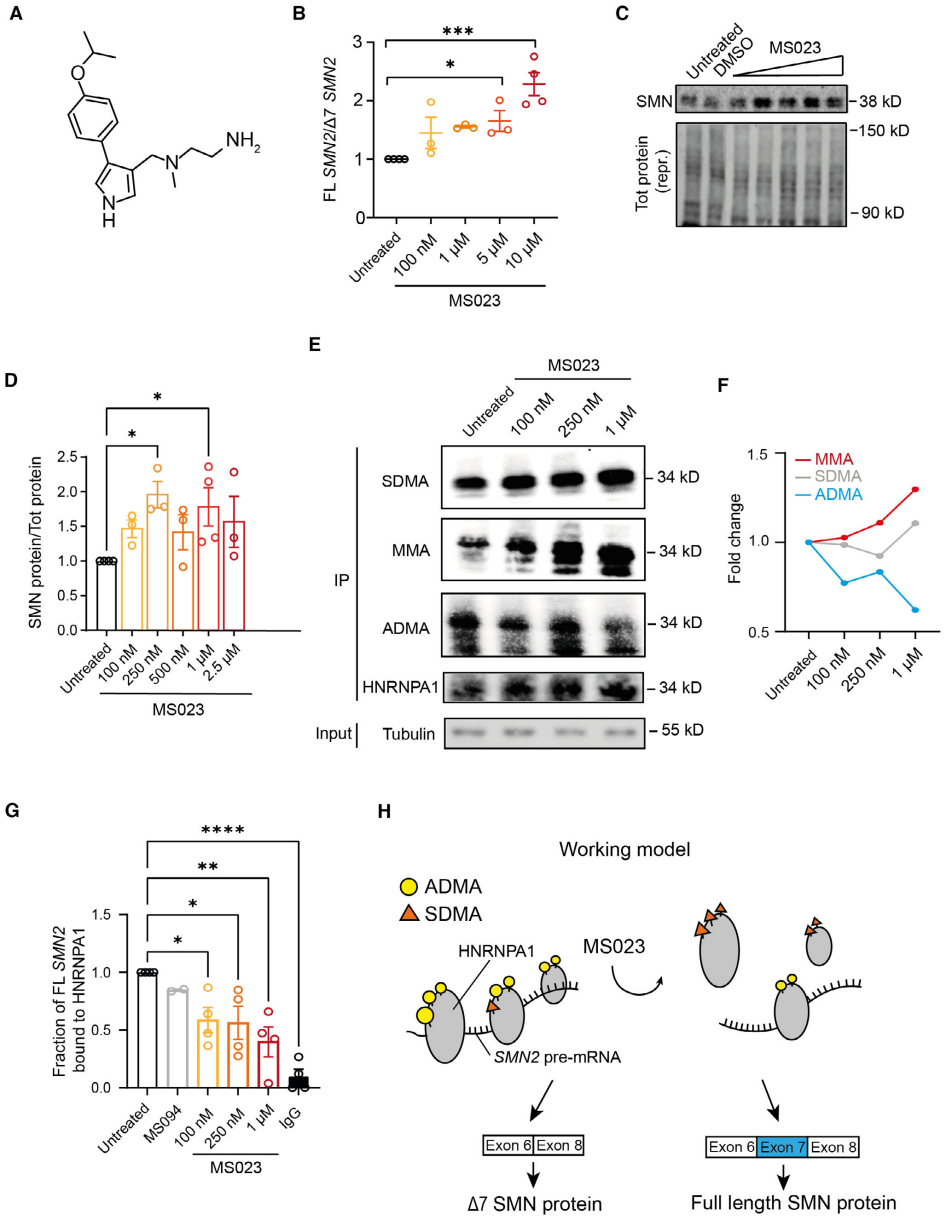
MS023 promotes exon 7 inclusion into *SMN2* transcript via HNRNPA1 binding inhibition AChemical structure depiction of MS023 (PubChem CID: 92136227).B–DSMA type II patient‐derived fibroblasts were treated with the indicated concentration of MS023 (range: 100 nM–10 μM), (*n* = 3–4). Cells were harvested for RNA (B) and protein (C, D) quantification 48 and 72 h post treatment, respectively (B). Full‐length (FL) *SMN2* transcript levels relative to Δ7 *SMN*2 are expressed as fold change compared to untreated SMA fibroblasts, normalised to one. Each dot represents a biological replicate (*n* = 3–4). (C) Western blot showing SMN protein levels upon treatment with increasing MS023 concentrations (top). A representative section of total protein stain, used for protein normalisation, is shown (bottom). The size in kilodalton is indicated on the right. (D) Quantification of SMN protein levels relative to total protein is shown. Each dot represents a biological replicate (*n* = 3–4).EArginine monomethylation (MMA) and symmetric dimethylation (SDMA) of HNRNPA1 are increased and asymmetric dimethylation (ADMA) of HNRNPA1 is decreased upon MS023 treatment, as shown by HNRNPA1 immunoprecipitation, followed by a Western blot. SMA type II patient‐derived fibroblasts were treated with the indicated concentration of MS023 (range: 100 nM–1 μM). HNRNPA1 was immunoprecipitated and Western blots were performed using anti‐MMA, anti‐SDMA, anti‐ADMA, anti‐HNRNPA1, and anti‐tubulin antibodies.FQuantification of arginine methylation changes normalised to total HNRNPA1 is shown.GRatio of FL *SMN2* transcripts bound to HNRNPA1 protein and relative to *GAPDH* was assayed using crosslinking immunoprecipitation (CLIP) in SMA patient fibroblasts treated with increasing concentrations of MS023. IgG antibody and MS094 were used as controls, MS094 is a negative control for MS023 lacking PRMT inhibition property. Each dot represents a biological replicate (*n* = 2–4).HRepresentation of the mechanism of exon 7 inclusion into the *SMN2* transcript by MS023. Blue circles, purple squares, and red triangles represent asymmetric dimethylarginine (ADMA), symmetric dimethylarginine (SDMA), monomethylarginine (MMA), respectively. Data information: (B), (D), and (G) Data are represented as mean ± s.e.m. and compared with a one‐way ANOVA test with multiple comparisons (*P ≤ 0.05; **P ≤ 0.01; ***P ≤ 0.001; ****P ≤ 0.0001). Source data are available online for this figure.

**Figure EV2:**
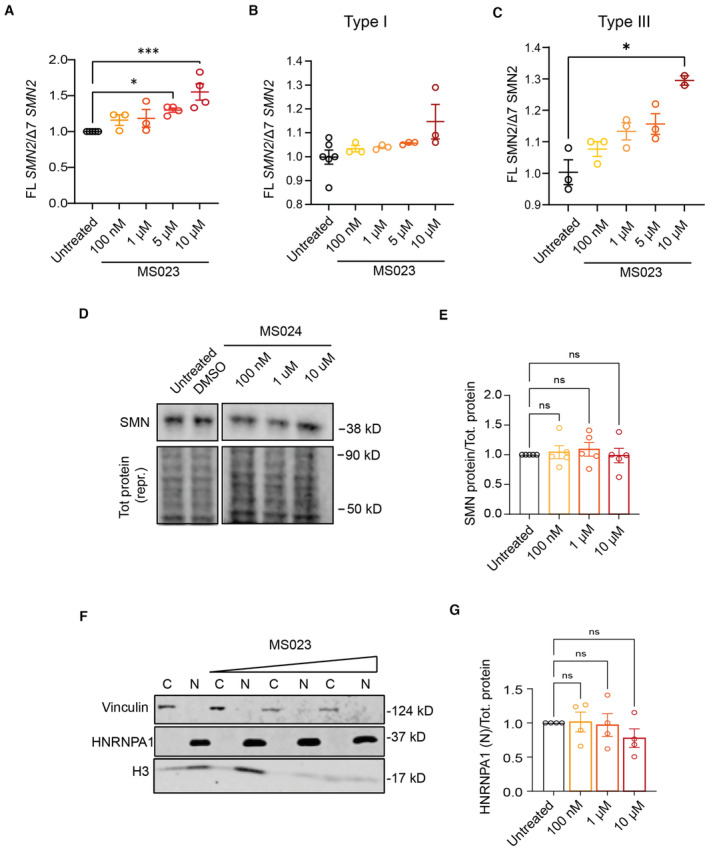
The increase in *SMN2* exon 7 inclusion by MS023 is specific and does not depend on altered HNRNPA1 levels ASMA type II patient‐derived fibroblasts were treated with the indicated concentration of MS023 (range: 100 nM–10 μM), (*n* = 3–4). Cells were harvested for RNA after 48 h incubation. Tot *SMN2* transcript levels relative to *GAPDH* are expressed as fold change compared to untreated SMA fibroblasts, normalised to one. Each dot represents a biological replicate (*n* = 3–4).B, CFull‐length (FL) SMN2 transcript levels relative to Δ7 SMN2 are expressed as fold change compared to untreated SMA type I and type III fibroblasts, normalised to one. Each dot represents a biological replicate (*n* = 2–6).DWestern blot showing SMN protein levels upon treatment with increasing MS094 (MS023 negative control) concentrations (top). A representative section of total protein stain, used for protein normalisation, is shown (bottom). The size in kilodalton is indicated on the right.EQuantification of SMN protein levels relative to total protein is shown. Each dot represents a biological replicate (*n* = 5).FWestern blot showing vinculin protein (top), HNRNPA1 (middle), and histone 3 (bottom) levels upon treatment with increasing MS023 concentrations and in the cytoplasm (C) and nucleus (N). The size in kilodalton is indicated on the right.GQuantification of HNRNPA1 protein levels relative to total protein is shown. Each dot represents a biological replicate (*n* = 4). Data information: (A), (B), (C), (E), and (G), Data are represented as mean ± s.e.m. and compared with a one‐way ANOVA test with multiple comparisons (*P ≤ 0.05; **P ≤ 0.001).

MS023 is a recently identified potent and selective inhibitor of type I PRMTs harbouring an ethylenediamine group, a critical moiety for its activity (Fig 2A; Eram *et al*, 2016). Treatment of type II SMA fibroblasts with MS023 led to a dose‐dependent increase in both *SMN2* exon 7 inclusion and protein levels (Figs 2B–D and EV2A and Appendix Fig S1), and this effect was confirmed also in fibroblasts from severe (type I) and mild (type III) SMA patients (Fig EV2B and C). No change in SMN levels was observed upon treatment with MS094 (Fig EV2D and E), an inactive MS023 analogue where the terminal primary amino group in the ethylenediamine group is replaced with a hydroxyl group (Eram *et al*, 2016), further confirming its dependency on PRMT activity. In order to identify the PRMT substrate mediating the effect of MS023 on *SMN2*, we interrogated a recently published dataset of the arginine methyl proteome in human NB4 cells upon treatment with MS023 (Fong *et al*, 2019). Out of 72 responsive targets, we noted that MS023 determined largely a downregulation of asymmetric dimethylarginine (ADMA) and an increase in monomethylarginine (MMA) sites in the heterogeneous nuclear ribonucleoprotein A1 (HNRNPA1), alongside changing the methylation pattern of other splicing factors implicated in *SMN2* splicing regulation (Fong *et al*, 2019). HNRNPA1 directly binds *SMN2* pre‐mRNA across multiple sites and is a well‐established negative regulator of exon 7 splicing (Cartegni *et al*, 2006; Kashima *et al*, 2007b; Bose *et al*, 2008; Chen *et al*, 2008; Hua *et al*, 2008; Koed Doktor *et al*, 2011; Xiao *et al*, 2012; Singh *et al*, 2013). Given that PRMT‐regulated methylation regulates binding of RNA‐binding proteins to RNAs (Blanc & Richard, 2017), we hypothesised that MS023‐induced ADMA‐to‐MMA switch in HNRNPA1 methylation affects its binding to *SMN*2 pre‐mRNA, resulting in exon 7 inclusion. In order to test this hypothesis, we treated SMA fibroblasts with increasing concentrations of MS023 and confirmed a dose‐responsive increase in both symmetric dimethylarginine (SDMA) and MMA methylation and a concomitant reduction in ADMA levels of HNRNPA1 protein (Fig 2E and F; Appendix Fig S2). Concomitantly, we observed a dose‐responsive reduction of the fraction of FL *SMN2* transcripts bound to HNRNPA1, as determined by a crosslinking and immunoprecipitation (CLIP) assay (Fig 2G), which validated our working model (Fig 2H). Treatment with MS023 did not change HNRNPA1 levels nor its subcellular localisation, further suggesting that the treatment‐induced change in arginine methylation specifically affects HNRNPA1 binding affinity with its RNA target (Fig EV2F and G). Taken together, these findings indicate that MS023 promotes *SMN2* exon 7 inclusion via decreased binding of HNRNPA1 to *SMN2* pre‐mRNA.

### Oral administration of MS023 improves the phenotype of SMA mice alone and in synergy with nusinersen

**Figure 3:**
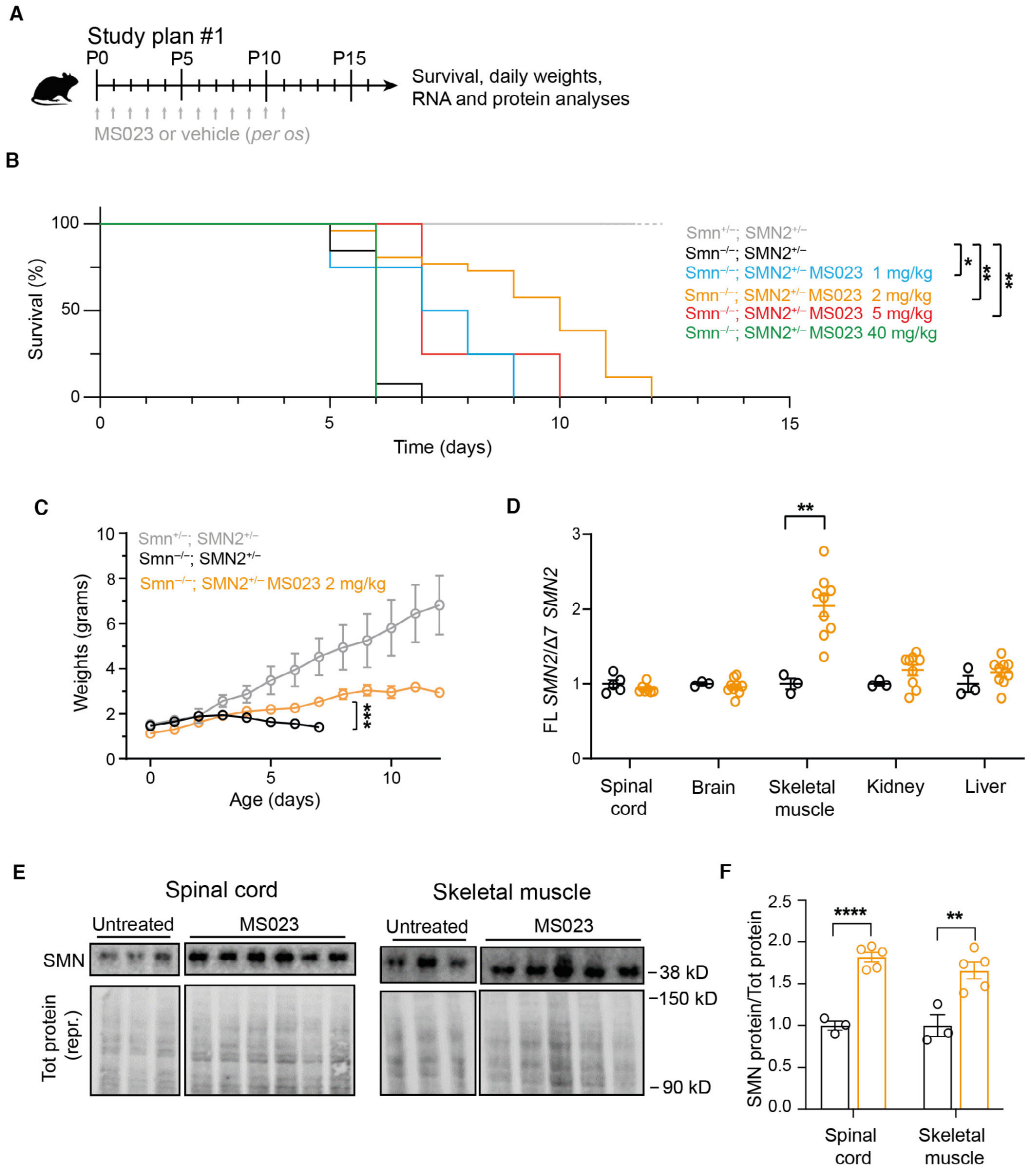
Oral administration of MS023 improves the phenotype of SMA mice Diagram of the study design: SMA mice were treated daily with oral administrations of MS023 or vehicle (0.5% DMSO in saline) from postnatal day 0 (P0) using a Hamilton syringe.Kaplan‐Meier survival estimation of unaffected mice (Smn^+/−^; SMN2^+/−^) and SMA affected mice (Smn^−/−^; SMN2^+/−^) treated with vehicle (*n* = 14), 1 mg/kg MS023 (*n* = 4), 2 mg/kg MS023 (*n* = 26), 5 mg/kg MS023 (*n* = 4), or 40 mg/kg MS023 (*n* = 4).Body weights of unaffected (*n* = 12), MS023‐treated (*n* = 26), and vehicle‐treated (*n* = 12) SMA mice from postnatal day 0 are shown.FL *SMN2* transcript levels relative to Δ7 *SMN2* in the spinal cord and skeletal muscle of treated mice compared to vehicle‐treated mice, normalised to one. Each dot represents a biological replicate (*n* = 3–9).Western blot showing SMN protein levels upon MS023 treatment in spinal cords and skeletal muscles of treated mice (top). A representative section of total protein stain, used for protein normalisation, is also shown (bottom). The size in kilodalton is indicated on the right.Quantification of SMN protein levels in the spinal cord and skeletal muscle relative to total protein is shown. Each dot represents a biological replicate (*n* = 3–8). Data information: (B–D), and (F), Data are compared with Mantel‐Cox test (*P ≤ 0.05; **P ≤ 0.01) (B) or represented as mean ± s.e.m. and compared with a one‐way ANOVA test with multiple comparisons (**P ≤ 0.01; ***P ≤ 0.001, ****P ≤ 0.0001) (C, D, F). Source data are available online for this figure.

**Figure 4:**
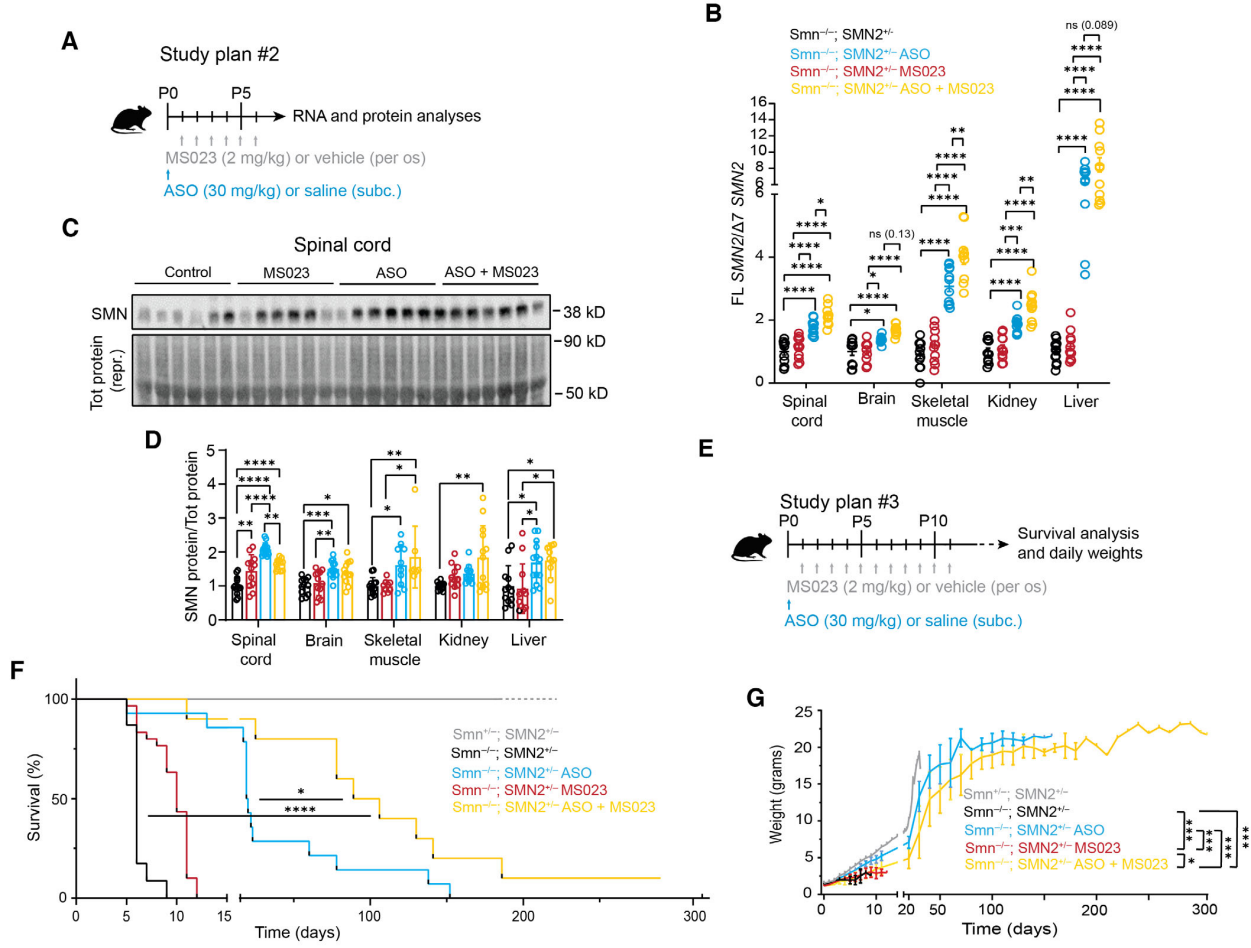
Combinatorial treatment with MS023 and ASO exerts synergistic effects in SMA mice Diagrams of the study design. Study plan #2: At P0 SMA mice were injected subcutaneously with *SMN2*‐targeting ASO (30 mg/kg) or saline; from P1 mice were treated daily with oral administrations of MS023 (2 m/kg) or vehicle (0.5% DMSO in saline) using a Hamilton syringe until P6. At P7, mice were sacrificed and tissues collected for analysis.FL *SMN2* transcript levels relative to Δ7 *SMN2* in spinal cord, brain, skeletal muscle, kidney and liver of treated SMA mice compared to vehicle‐treated SMA mice were normalised to one. Each dot represents a biological replicate (*n* = 10–12).Western blot showing SMN protein levels upon the indicated treatments in spinal cords of SMA mice (top). A representative section of total protein stain, used for protein normalisation, is also shown (bottom). Size in kilodalton is indicated on the right.Quantification of SMN protein levels relative to total protein is shown. Each dot represents a biological replicate (*n* = 7–13).Study design #3: At P0 mice were injected subcutaneously with *SMN2*‐targeting ASO (30 mg/kg) or saline. From P1 mice were treated daily with oral administrations of MS023 (2 m/kg) or vehicle (0.5% DMSO in saline) using a Hamilton syringe until P12. Mice were weighed daily until they reached their humane end point.Kaplan–Meier survival estimation of unaffected mice (Smn^+/−^; SMN2^+/−^) and SMA affected mice (Smn^−/−^; SMN2^+/−^) treated with *SMN2*‐targeting ASO (*n* = 15), MS023 (*n* = 14), *SMN2*‐targeting ASO and MS023 (*n* = 10), and vehicle (*n* = 23).Body weights of unaffected (*n* = 12) and SMA mice (vehicle‐treated: *n* = 7; ASO: *n* = 13; MS023; *n* = 22; ASO + MS023: *n* = 10) from postnatal day 0 are shown. Unaffected mice were followed until 25 weeks of age. Data information: (B), (D), (F), and (G), Data are represented as mean ± s.e.m. and compared with a one‐way ANOVA test with multiple comparisons (*P ≤ 0.05; **P ≤ 0.01; ***P ≤ 0.001, ****P ≤ 0.0001) (B, D, G) or with a Mantel‐Cox test (*P ≤ 0.05; ****P ≤ 0.0001) (F). Source data are available online for this figure.

**Figure EV3:**
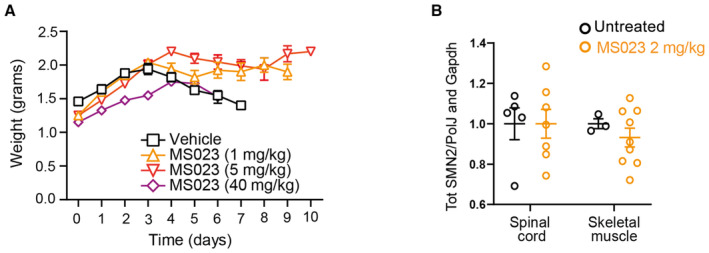
Oral administration of MS023 improves the phenotype of SMA mice Body weights of untreated (*n* = 12), MS023‐ (1 mg/kg: *n* = 10; 5 mg/kg: *n* = 9; 40 mg/kg: *n* = 4), or vehicle‐treated (*n* = 5) SMA mice from postnatal day 0 are shown.Tot *SMN2* transcript levels relative to *PolJ* and *Gapdh* in spinal cord and skeletal muscle of treated SMA mice compared to vehicle‐treated SMA mice, normalised to one. Each dot represents a biological replicate (*n* = 10–12). Data information: (A, B) Data are represented as mean ± s.e.m. and compared with a one‐way ANOVA test with multiple comparisons.

**Figure EV4:**
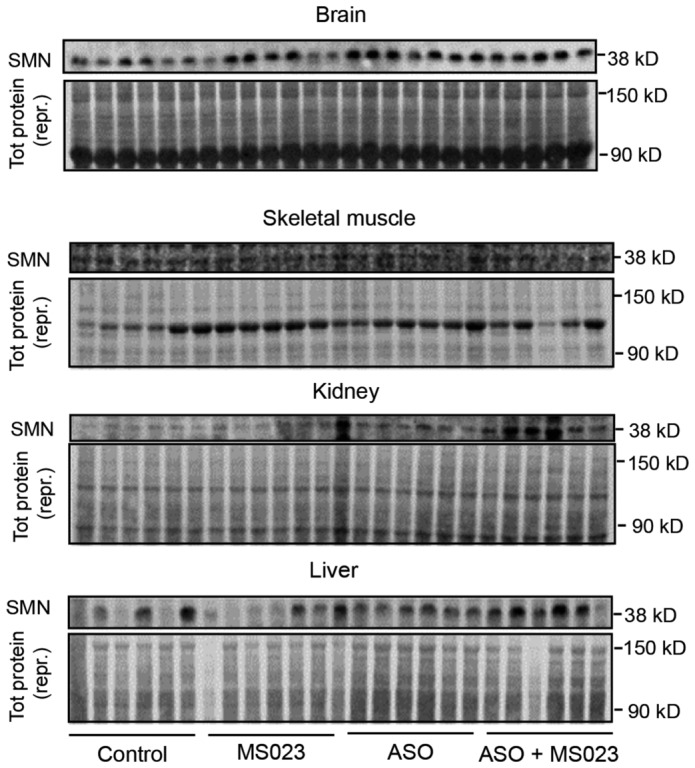
Combinatorial treatment with MS023 and ASO exerts synergistic effects in SMA mice Western blot showing SMN protein levels following the indicated treatments in the brain, skeletal muscle, kidney, and liver of SMA mice (top). A representative section of total protein stain, used for protein normalisation, is also shown (bottom). The size in kilodalton is indicated on the right.

We next evaluated the efficacy and tolerability of MS023 treatment *in vivo* in a severe preclinical mouse model of SMA (Hsieh‐Li *et al*, 2000). These mice, which lack the mouse *Smn* gene and only carry a single copy of the human *SMN2* gene (Smn^−/−^; SMN2^+/−^), display a phenotype with weight loss and reduced motor activity starting at postnatal day 5 (P5) and typically reach a humane end point by P9. Daily oral administration of MS023 or vehicle (0.5% DMSO in 0.9% saline solution) was performed in SMA mice from P0 until reaching a humane end point (Fig 3A). We tested a range of doses (1, 2, 5, and 40 mg/kg) and found that treatment with both 2 and 5 mg/kg resulted in a significant increase in survival, with the 2 mg/kg dose achieving the best effect (median: 10 days) compared to vehicle‐treated mice (median: 6 days; *P* < 0.0001) (Fig 3B). Mice treated with this regimen also showed an improvement in disease‐associated weight loss (Fig 3C). No further amelioration was observed with 40 mg/kg MS023, suggesting that with this dose the therapeutic window has been surpassed (Figs 3B and EV3A). Notably, we detected an increase in full‐length (FL) *SMN2* transcript in skeletal muscle and SMN protein levels in the both spinal cord and skeletal muscle, the tissues mostly affected in the disease (Mercuri *et al*, 2022), of SMA mice treated with 2 mg/kg MS023 (Figs 3D–F and EV3B). Since the mechanism of action of the 2′‐O‐methoxyethyl phosphorothioate‐modified drug nusinersen, a currently approved ASO therapy for SMA patients, consists of promoting exon 7 inclusion into *SMN2* pre‐mRNA by blocking the recruitment of HNRNP splicing repressors at the ISS‐N1 site (Chiriboga *et al*, 2016; Finkel *et al*, 2016, 2017; Haché *et al*, 2016; Mercuri *et al*, 2018), we postulated that a combinatorial treatment with MS023 would synergistically lead to improved therapeutic benefit and allow for a cost‐effective ASO dosing regimen in SMA. In order to test this hypothesis, at P0 SMA mice were treated with a single subcutaneous administration of 30 mg/kg nusinersen, a suboptimal dose sufficient to slightly extend the SMA mice life span (Hammond *et al*, 2016), alone or in combination with daily oral administration of 2 mg/kg MS023 from P1 to P6 (Fig 4A). Analysis of tissues collected at P7 showed that the combinatorial treatment was able to further enhance exon 7 inclusion in *SMN2* pre‐mRNA (Fig 4B) and increase SMN protein levels (Figs 4C and D and EV4), both in the CNS and peripheral tissues. A further follow‐up study designed to assess the effect on survival (Fig 4E) revealed that the combinatorial treatment of nusinersen and MS023 dramatically prolonged the lifespan (median: 97.5 days) compared to nusinersen alone (median: 19.5; *P* = 0.02; Fig 4F) and improved the body weight of SMA mice (Fig 4G). Overall, these results suggest that oral administration of MS023 synergises with nusinersen to provide a therapeutic benefit in SMA.

### Molecular signature of the combinatorial treatment

**Figure 5:**
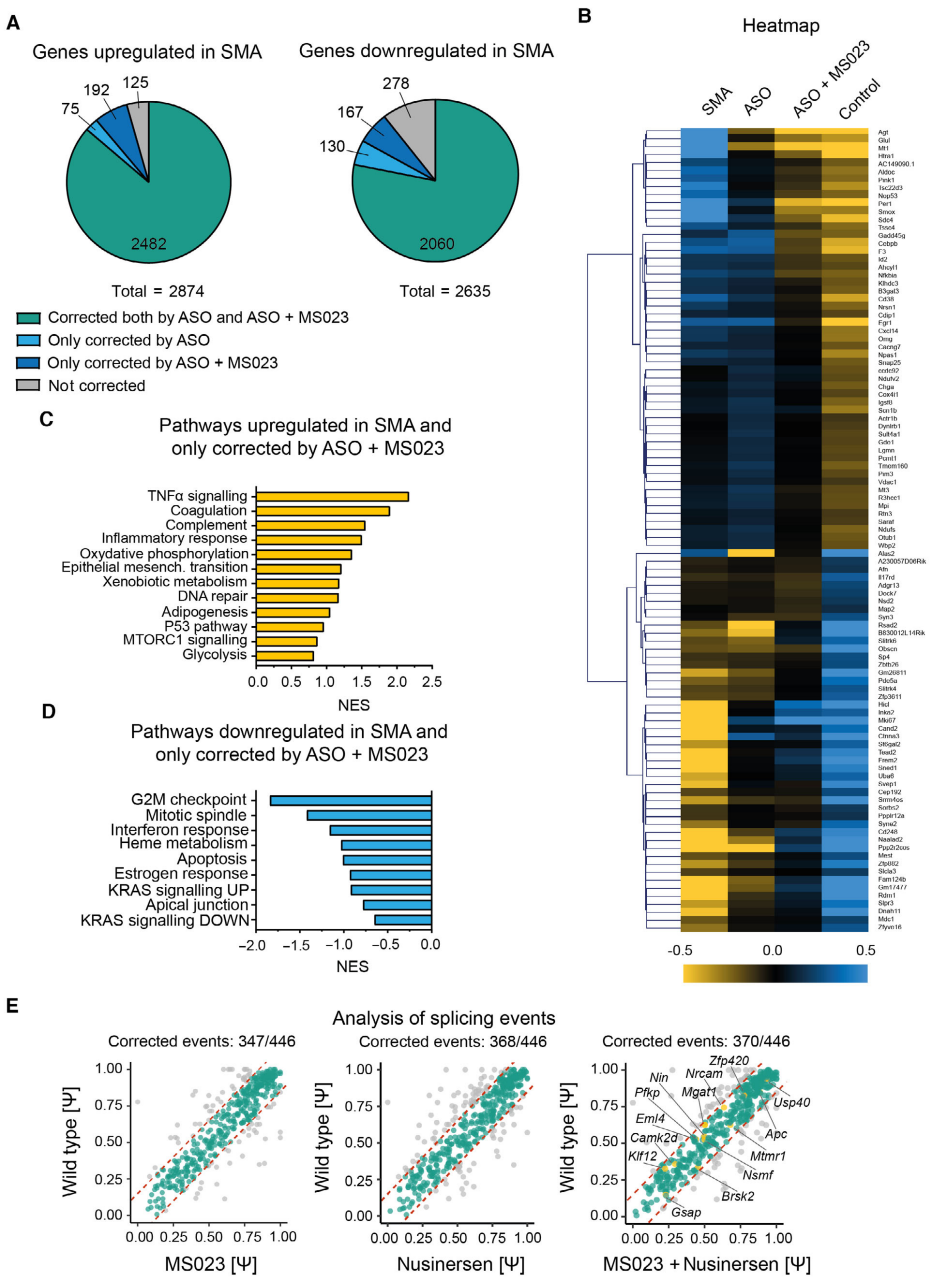
Combinatorial treatment with MS023 and ASO results in improved correction of the SMA transcriptomic signature compared to ASO alone APie charts showing the proportion of transcripts normalised upon the treatments among the genes that are upregulated (left) and downregulated (right) in SMA mice.BNormalised counts were used to generate the hierarchical clustering heatmap. Upregulated and downregulated genes are displayed in yellow and blue, respectively. Control refers to unaffected (Smn^+/−^; SMN2^+/−^) mice.C, D(C) Hallmark pathway analysis of genes upregulated and (D) downregulated in SMA corrected by the combinatorial treatment only.EPlot charts show the distribution of splicing events (Ψ) in SMA mice upon treatment relative to wild‐type littermates. The red dotted lines mark the ± 15% normalisation range, corrected values are indicated in green.

**Figure EV5:**
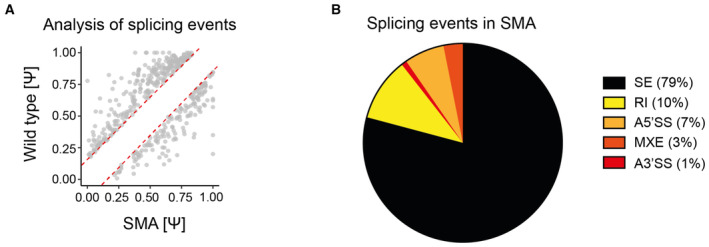
Altered splicing events in SMA Plot charts show the distribution of 446 aberrant splicing events (Ψ) in SMA mice relative to wild‐type littermates. The red dotted lines mark the ±15% normalisation range.Pie chart showing the proportion of altered splicing events in the spinal cord of SMA mice compared to wild type (A3′SS, alternative 3′ splice site; A5′SS, alternative 5′ splice site; MXE, mutually exclusive exon; RI, intron retention; SE, skipped exon).

In order to understand the molecular underpinnings of the added benefit provided by the combinatorial treatment, we performed a bulk transcriptomic analysis in the spinal cords of symptomatic (P7) SMA (Smn^−/−^; SMN2^+/−^) mice treated with MS023 only, nusinersen only, and MS023 in combination with nusinersen, compared to untreated SMA and unaffected (Smn^+/−^; SMN2^+/−^) mice, following the treatment paradigm described above (Fig 4A). Out of a total of 5,509 significantly dysregulated transcripts in SMA (*P* < 0.05, false discovery rate < 0.05), treatment with MS023 only, nusinersen only, and MS023 in combination with nusinersen resulted in the correction of 4,270 (77.5%), 4,747 (86.2%), and 4,901 (88.9%) targets, respectively (Dataset EV1). Combinatorial treatment was able to exclusively rescue 192/2874 (6.7%) of the upregulated genes and 167/2635 (6.3%) of the downregulated genes that were not corrected upon treatment with nusinersen alone (Fig 5A; Dataset EV2). We generated a heatmap of the top significant genes (*P* < 0.01) of this category (Fig 5B) and performed a hallmark analysis to depict and identify the changes that could explain the beneficial effects of the combinatorial treatment (Fig 5C and D). TNF‐α signalling, together with several other immune‐related pathways, including interferon response and complement activation, were highly enriched, overall suggesting that targeting neuroinflammation for therapy is key to achieving a beneficial effect in SMA. Interestingly, astrocyte dysfunction and chronic microglia activation have been observed early in SMA and other neurological conditions (Eikelenboom *et al*, 2002; Sargsyan *et al*, 2005; Heneka *et al*, 2014; Vukojicic *et al*, 2019), and their contribution to neuronal dysfunction and death is abundantly reported (Mcgivern *et al*, 2013; Rindt *et al*, 2015; Zhou *et al*, 2016; Martin *et al*, 2017). Notably, the combinatorial treatment showed a good safety profile, changing the expression of only 160 off‐target genes (i.e. unrelated to SMA) (Dataset EV3). Given the role of SMN in RNA biogenesis and spliceosomal protein assembly (Pellizzoni *et al*, 2001) and the observation of increased mis‐splicing events upon SMN depletion (Zhang *et al*, 2008, 2013; Bäumer *et al*, 2009; Huo *et al*, 2014; Custer *et al*, 2016; Doktor *et al*, 2017), we wondered whether the beneficial effect of the combinatorial treatment was due to restoration in the splicing profile. We identified 446 mis‐splicing events in the spinal cord of SMA mice, with the vast majority (*n* = 353, 79%) being skipped exons, in accordance with previous reports (Zhang *et al*, 2008, 2013; Bäumer *et al*, 2009; Huo *et al*, 2014; Custer *et al*, 2016; Doktor *et al*, 2017; Fig EV5A and B). Individual treatments with MS023 or nusinersen were both able to restore the splicing profile almost fully (347/446 and 368/446, respectively), with only 14 unique splicing events corrected exclusively upon the combinatorial treatment (Fig 5E; Table EV2), suggesting that mis‐splicing has a low threshold for normalisation in SMA and is a poor predictor of treatment response.

## Discussion

Thousands of SMA patients are treated each year with nusinersen, the first SMA treatment to receive FDA and EMA approval in 2016 and 2017, respectively. Despite having enormously changed the life expectancy and quality of life of patients, this approach is not without limitations: it fails to address the peripheral manifestations of the disease, is costly, and the intrathecal administrations require patients' hospitalisation and well‐trained clinicians, becoming more troublesome with time as the disease progresses (Mercuri *et al*, 2020). Research efforts are underway to develop both SMN‐dependent and SMN‐independent therapies for improved and sustained benefit and to clinically evaluate the safety and efficacy of combinatorial approaches in SMA patients (NCT04488133, NCT03032172).

From a screen of potent and highly selective epigenetic probes, we have identified MS023, a potent and selective next‐generation type I PRMT inhibitor (Eram *et al*, 2016), able to elevate SMN protein in SMA models at levels comparable to recently clinically approved small molecule risdiplam (Naryshkin *et al*, 2014) and to synergistically amplify the effects of nusinersen. Interestingly, targeting type II PRMTs by selective PRMT5 inhibitors GSK591 and LLY‐283 reduced FL *SMN2*, supporting a mechanism of regulation of *SMN2* by this class of enzymes. When assessing the *in vivo* effects of MS023 treatment, we observed a significant increase in SMN protein in the spinal cords of SMA mice, while transcript levels remained unchanged (Fig 3D–F), possibly suggesting that the dose of 2 mg/kg used in this study is at the threshold for correction. Additionally, *SMN2* mRNA levels have been shown to rapidly return to baseline within 32 h after both single and repeated dosing of splicing‐inducing small molecules such as risdiplam, while the SMN protein continues to increase (Naryshkin *et al*, 2014), and *SMN2* mRNA expression poorly correlates with protein expression in spinal cord samples from human SMA subjects (Ramos *et al*, 2019). Overall, these data hint at mechanisms other than transcriptional activity or splicing, such as translational efficiency and/or protein stability, that may contribute to SMN protein expression in spinal cord (Ramos *et al*, 2019).

Protein arginine methylation is a prevalent posttranscriptional modification occurring at the nitrogen atoms of the guanidinium group of over 4,000 proteins, many of which are RNA‐binding proteins, influencing their stability, function, and interaction with other macromolecules (Hornbeck *et al*, 2015). In mammals, arginine methylation of histone and non‐histone proteins is performed by nine PRMT enzymes, divided into three families: Type I PRMTs (PRMT1, PRMT2, PRMT3, PRMT4, PRMT6, and PRMT8) perform monomethylation followed by an asymmetric dimethylation (ADMA) of arginine, with both methyl groups on a single guanidino nitrogen atom; type II PRMTs (PRMT5 and PRMT9) catalyse monomethylation, followed by a symmetric dimethylation (SDMA) where one methyl group is transferred to each of the nitrogen atoms; and type III PRMT (PRMT7) only performs monomethylation (MMA) (Guccione & Richard, 2019). PRMTs have been shown to compete over the same substrates, frequently with opposite functional effects (Dhar *et al*, 2013).

Several studies have identified a link between PRMTs and SMN: to ensure fidelity of loading onto the correct snRNAs, three of the core spliceosomal Sm proteins (SmB, SmD1, and SmD3) undergo symmetric dimethylation by PRMT5, a modification recognised by the Tudor domain of SMN (Meister *et al*, 2001; Friesen *et al*, 2001b). SMN interaction with senataxin, a DNA‐RNA helicase, depends on the dimethylated arginine and is reduced upon PRMT5 knockdown (Yanling Zhao *et al*, 2016). Lastly, levels of PRMT4 (CARM1) are upregulated in SMA mice spinal cord and patients' cells (Sanchez *et al*, 2015). PRMTs represent a promising therapeutic target for many human diseases, from cancer (Wang *et al*, 2016; Drew *et al*, 2017; Wu *et al*, 2022) to neurodegeneration (Dormann *et al*, 2012; Scaramuzzino *et al*, 2015; Suárez‐Calvet *et al*, 2016), with eight PRMT inhibitors attaining clinical trial testing in human cancers (Yang & Bedford, 2013; Blanc & Richard, 2017; Guccione & Richard, 2019; Hwang *et al*, 2021). Consistent with previous methylome analyses in cells treated with type I PRMT inhibitors, GSK3368712 (Noto *et al*, 2020) or MS023 (Karuppagounder *et al*, 2016), we propose that the MS023 mechanism of action entails a switch in the arginine methylation profile from ADMA to SDMA/MMA of HNRNPA1, a major negative regulator of *SMN2* exon 7 inclusion (Cartegni *et al*, 2006; Kashima *et al*, 2007b; Bose *et al*, 2008; Chen *et al*, 2008; Hua *et al*, 2008; Koed Doktor *et al*, 2011; Xiao *et al*, 2012; Singh *et al*, 2013), resulting in reduced binding affinity to *SMN2* pre‐mRNA. This mechanism of action is different from the one recently proposed for risdiplam, which entails stabilisation of a yet unidentified RNP complex by binding to distinct sites of *SMN2* pre‐mRNA (Sivaramakrishnan *et al*, 2017). Notably, HNRNPA1 has been shown to modulate exon 7 splicing by directly binding *SMN2* pre‐mRNA across multiple sites, which include enhancer regions SE1 and SE2, as well as the ISS‐N1 region (Lejman *et al*, 2021). Therefore, here we postulate that MS023 enhances the effect of nusinersen by preventing HNRNPA1 multi‐site binding and concomitantly increasing the overall accessibility of the oligonucleotide to the ISS‐N1 region. Which are the treatment‐responsive HNRNPA1 methylation sites and how HNRNPA1 binding to *SMN2* pre‐mRNA changes upon MS023 remain open questions. They could be addressed with techniques such as heavy methyl SILAC (Ong *et al*, 2004) and label‐free surface plasmon resonance technology to assess MS023‐induced changes in methylation profiles and target binding affinity to surface‐immobilised *SMN2* exon 7, respectively. Out of the 72 targets identified by arginine methyl proteome profiling of human cells (Fong *et al*, 2019), MS023 changed the methylation pattern of other splicing factors implicated in the regulation of *SMN2* splicing (i.e. HNRNPU, HNRNPA2B1, ELAVL1, RBM10, KHDRBS1, SRSF9, TRA2B, and U2AF1; Singh & Singh, 2018), and the individual contribution of such proteins to the overall effect of this small molecule in SMA models remains to be established.

Hallmark analysis of the 359 genes whose expression levels in spinal cords were only corrected by the combinatorial treatment revealed enrichment of immune‐related pathways, such as TNF‐α signalling, interferon response, and complement activation, overall suggesting that therapeutic targeting of neuroinflammation is key to achieving optimal and long‐lasting effects in SMA. Interestingly, astrocyte dysfunction and chronic microglia activation have been observed early in SMA and other neurological conditions (Eikelenboom *et al*, 2002; Sargsyan *et al*, 2005; Heneka *et al*, 2014; Vukojicic *et al*, 2019) and play a determinative role in the disease pathogenesis (Mcgivern *et al*, 2013; Rindt *et al*, 2015; Zhou *et al*, 2016; Martin *et al*, 2017). Transcriptomic data in the preclinical model indicates that MS023 shows a favourable safety profile, with minimal off‐target effects both on gene expression and splicing alterations, probably due to the low dose required to achieve *SMN* modulation compared to cancer applications (i.e. 80 mg/kg/day; Fong *et al*, 2019) and in stark contrast to the pleiotropic effects of other epigenetic modulators such as valproic acid.

Altogether, these promising preclinical results warrant further clinical investigations of MS023 or other selective type I PRMTs inhibitors both as a stand‐alone and as an add‐on treatment with nusinersen in SMA patients.

## Materials and Methods

### Small molecules screening

The library of small molecules was obtained from the Structural Genomic Consortium (SGC) (Williamson, 2000). Upon reception the molecules were diluted in DMSO to 10 mM, aliquoted and stored at −20°C for the duration of the study. All the compounds, their targets and doses used in the study are listed in Table EV1.

### Cell lines and culture

Cells were grown in a humidified incubator at 37°C with 5% CO_2_. SMA type I and II patient fibroblasts were obtained from Coriell Institute (GM00232, GM03813). SMA type III patient fibroblasts were a kind gift of the Talbot's lab (University of Oxford). The cells were maintained in Dulbecco's Modified Eagle Medium GlutaMAX (Gibco) supplemented with 10% foetal bovine serum (Gibco) and 1× Antibiotic‐Antimycotic (Gibco). SMA patient fibroblasts were plated in triplicates, in 12‐well plates at 50,000 cells per well in 500 μl of medium for RNA or in duplicates in 6‐well plates at 100,000 cells per well in 1 ml of medium for protein. After 6 h, compounds of interest were diluted in medium to 2× final concentration and added to the cells. RNA was isolated after 48 h incubation, protein after 72 h.

### Cell viability assay

Cells were routinely tested for mycoplasma. The MTS Cell Proliferation Assay Kit (Abcam) was used to determine the highest non‐toxic concentration of small molecules in SMA patient fibroblasts. Briefly, 3,000 cells/wells were plated in triplicate in a 96‐well plate in half of the final volume of medium (100 μl). After 6 h, compounds of interest were diluted to 2× final concentration in 100 μl of medium and added in a range of concentrations (1–10 μM). 1 μM of staurosporine (Sigma) was used as a positive control for cell death. After 48 h, 20 μl of MTS reagent was added to each well. Four hours later, the absorbance was measured at 490 nm on CLARIOstar plate reader (BMG Labtech). The concentration was deemed non‐toxic if the absorbance was not significantly different from the control and the cells looked viable after visual inspection. In subsequent experiments, only the highest non‐toxic concentration of each compound was used, unless stated otherwise.

### RNA/cDNA preparation and RT‐qPCR

RNA extraction was performed using the Maxwell® RSC simply RNA Kit (Promega) following the manufacturer's instructions. The concentration was measured using a Nanodrop 1000 spectrophotometer (Thermo Fisher), and cDNA was generated using an ABI High Capacity cDNA Reverse Transcription Kit (Invitrogen) following the manufacturer's instructions. A qPCR reaction using Power SYBR Green Master Mix (Life Technologies) was performed and analysed on an Applied Biosystems StepOnePlus™ real‐time PCR system (Life Technologies). FL *SMN2*, Δ7 *SMN2*, Tot *SMN2*, *PolJ*, *Gapdh*, and *GAPDH* transcripts were amplified using gene‐ and species‐specific primers (Table EV3).

### Protein extraction and Western blot

Proteins were harvested from ~ 30 mg of tissue (*in vivo*) or two 6‐well plates (*in vitro*) and homogenised in RIPA buffer with complete mini‐proteinase inhibitors (Roche). The preparation of nuclear and cytoplasmic extracts from human fibroblasts was performed using NE‐PER Nuclear and Cytoplasmic Extraction Reagents (Thermo Fisher), as per manufacturer's instructions. Proteins (10–15 μg from cells and 20–30 μg from tissue) were probed for human SMN protein using anti‐SMN, clone SMN‐KH monoclonal IgG1 (Sigma, MABE230), Histone 3 (Cell Signalling, 9715), Vinculin (Sigma, 062M4762), HNRNPA1 (Santa Cruz, 4B10), MMA (Cell Signalling, 8015), ADMA (Sigma Aldrich, 07‐414), SDMA (Cell Signalling, 13222), β‐tubulin (Abcam, 108342), and FAST green total protein stain and secondary antibody IRDye 800CW goat anti‐mouse IgG (LI‐COR Biosciences). Membranes were imaged on a LI‐ COR Odyssey FC imager and analysed with Image Studio™ software (LI‐COR Biosciences).

### Crosslinking and immunoprecipitation assay (CLIP)

Briefly, for each condition, four 150 cm^2^ cell dishes of human patient fibroblasts were treated with 100 nM, 250 nM or 1 μM of MS023 for 48 h. After the incubation, the medium was aspirated, and the cells were subjected to 150 mJ/cm^2^ 254 nm UV light in a Stratalinker UV Crosslinker and pelleted. The cells were then lysed in NP‐40 lysis buffer and HNRNPA1 immunoprecipitation was performed with Dynabeads Protein G (Thermo Fisher) and the hnRNAP1 antibody (Santa Cruz). Following DNA and protein digestion, RNA was isolated and cDNA was generated as previously outlined. A qPCR reaction using Power SYBR Green Master Mix (Life Technologies) was performed and analysed on an Applied Biosystems StepOnePlus™ real‐time PCR system (Life Technologies). FL *SMN2* and *GAPDH* transcripts were amplified using gene‐ and species‐specific primers.

### Protein immunoprecipitation

To detect changes in HNRNPA1 arginine methylation, SMA patient fibroblasts were either left untreated or treated with increasing concentrations of MS023 (100 nM, 250 nM or 1 μM) for 48 h. Cell pellets were lysed in NP‐40 buffer, and HNRNPA1 immunoprecipitation was performed with Dynabeads Protein G (Thermo Fisher, 10003D) and hnRNAP1 antibody (Santa Cruz), following the manufacturer's protocol. Subsequently, Western blots were run according to the protocol described above.

### Mice

Mice were housed and all the procedures were carried out at the Biomedical Services Building, University of Oxford, and authorised by the UK Home Office in accordance with the Animals (Scientific Procedures) Act 1986 and by the University of Oxford ethics committee (PPL no: PDFEDC6F0). Mice were housed in biosecurity level 2, with a 12 h night/day cycle, in transparent plastic cages with daily water and food exchange. The pups were housed with their mother only (no father) until day 21, when they were weaned off into separate cages for males and females, with a maximum of six adult mice in one cage. Both male and female staff attended to the mice and performed experiments. ARRIVE guidelines were followed. All experiments were performed on the SMA mouse strain FVB.Cg‐Smn1^tm1Hung^Tg(SMN2)2Hung/J—the “Taiwanese” model (*Smn* ^−/−^; *SMN2* ^+/−^), generated and maintained as previously described (Hsieh‐Li *et al*, 2000; Gogliotti *et al*, 2010). MS023 was administered daily orally from P0 or P1 using a Hamilton syringe (Hamilton). Doses of 0, 1, 2, 5, or 40 mg/kg of MS023 were diluted in 0.5% DMSO and 0.9% saline and administered at a volume of 5 μl/g of body weight. Nusinersen (sequence: U*sC*sA*sC*sU*sU*sU*sC*sA*sU*sA*sA*sU*sG*sC*sU*sG*sG*s, where “S” is phosphorothioate backbone and “*” is a 2′‐O‐(2‐Methoxyethyl)‐oligoribonucleotides chemistry) was diluted in 0.9% saline and given once at 20 μl/g body weight, via subcutaneous injection at P0, in a dose of 30 mg/kg. Weights were recorded, and overall health was assessed daily. Since the pups were treated daily from P0 and identifying and marking individual pups between P0 and P7 poses a high risk of misidentifying an animal, in this study a litter constitutes an experimental unit, and all mice in the litter were subjected to the same treatment. Only litters between 7 and 11 pups were used to correct for average weight (mice in smaller litters tend to be bigger and live longer, and the opposite is true for litters of 12 and above), and treatment was allocated randomly to a litter before it was born. All the experimental units treated were included in the analysis. Personnel performing daily weights and welfare checks for combinatorial therapy were blinded; researcher performing oral administration, injections, and data analysis was not blinded. For survival analysis, the humane end point were reached upon 15% weight loss from a maximum weight or when the mouse was not able to right itself for 30 s. Mice were culled by decapitation (if younger than postnatal day 10) or cervical dislocation (if 10 days old or older). Tissues were harvested on the indicated postnatal day.

### RNA sequencing

Transcriptomic analysis was performed by Novogene (UK) Company Limited (https://en.novogene.com/) on P7 spinal cords of mice in the following treatment groups: untreated SMA mice, SMA mice treated with nusinersen, SMA mice treated with MS023, SMA mice treated with nusinersen and MS023, and untreated controls (four biological replicates in each group). RNA quantification and integrity were assessed using the RNA Nano 6000 Assay Kit of the Bioanalyzer 2100 system (Agilent Technologies, CA, USA). mRNA was purified using poly‐T oligo‐attached magnetic beads. Fragmentation was carried out using divalent cations under elevated temperatures in First Strand Synthesis Reaction Buffer. The first strand cDNA was synthesised using random hexamer primers and M‐MuLV Reverse Transcriptase. Second‐strand cDNA synthesis was subsequently performed using DNA Polymerase I and RNase H. The remaining overhangs were converted into blunt ends via exonuclease/polymerase activities. After adenylation of 3′ ends of DNA fragments, adaptor with hairpin loop structure were ligated to prepare for hybridisation. In order to select cDNA fragments of preferentially 370–420 bp in length, the library fragments were purified with AMPure XP system (Beckman Coulter, Beverly, USA). PCR products were purified (AMPure XP system) and library quality was assessed on the Agilent Bioanalyzer 2100 system. The clustering of the index‐coded samples was performed on a cBot Cluster Generation System using TruSeq PE Cluster Kit v3‐cBot‐HS (Illumina) according to the manufacturer's instructions. After cluster generation, the library preparations were sequenced on an Illumina Novaseq platform with a coverage of 25 million reads and 150 bp paired‐end reads were generated. Raw data (raw reads) of fastq format were firstly processed through in‐house perl scripts. In this step, clean data (clean reads) were obtained by removing reads containing adapter, reads 1 containing ploy‐N and low‐quality reads from raw data. Mus Musculus (GRCm38/mm10) reference genome was used, index of the reference genome was built using Hisat2 v2.0.5 and paired‐end clean reads were aligned to the reference genome using Hisat2 v2.0.5. The mapped reads of each sample were assembled by StringTie (v1.3.3b; Pertea *et al*, 2015) in a reference‐based approach. Quantification of gene expression level Feature Counts v1.5.0‐p3 was used to count the reads numbers mapped to each gene. Differential expression analysis was performed using the DESeq2 R package (1.20.0), Benjamini‐Hochberg‐adjusted *P*‐values reported. A corrected *P*‐value of 0.05 and absolute fold change of 2 were set as the threshold for significantly differential expression. Alternative splicing analysis rMATS (4.1.0) software was used to analysis the splicing event. We used the *psiPerEvent* operation of SUPPA to calculate the Ψ values from the transcript quantifications obtained for all the alternative splicing events generated as described above with the *generateEvents* module of SUPPA (Alamancos*et al*, 2014). The data were visualised with R (www.r-project.org/).

### Study approval

All animal procedures were authorised by the UK Home Office in accordance with the Animals (Scientific Procedures) Act 1986 and by the University of Oxford ethics committee (PPL no: PDFEDC6F0).

### Statistics

ANOVA tests were used to compare the means between two or more groups, respectively. Statistics of survival times of SMA mice were determined by Kaplan‐Meier estimation, and comparisons were made with the log‐rank test. A two‐way ANOVA was conducted to compare the effect of the treatment on the weights of the animals using treatment as a between‐subjects factor and time as a within‐subjects factor. Power analysis was performed using G*Power 3.1.9.2 software (Erdfelder *et al*, 2009). GraphPad Prism version 8 was used to perform the statistical analyses (GraphPad, La Jolla, CA). A *P*‐value < 0.05 was set as statistically significant.

## Author contributions

**Anna J Kordala:** Conceptualization; data curation; formal analysis; methodology; writing – original draft; writing – review and editing. **Jessica Stoodley:** Conceptualization; data curation; formal analysis; writing – review and editing. **Nina Ahlskog:** Data curation; formal analysis; writing – review and editing. **Muhammad Hanifi:** Data curation; formal analysis; writing – review and editing. **Antonio Garcia Guerra:** Data curation; formal analysis. **Amarjit Bhomra:** Data curation; formal analysis; writing – review and editing. **Wooi Fang Lim:** Data curation; writing – review and editing. **Lyndsay M Murray:** Writing – review and editing. **Kevin Talbot:** Resources; writing – review and editing. **Suzan M Hammond:** Conceptualization; writing – review and editing. **Matthew JA Wood:** Conceptualization; resources; funding acquisition; writing – review and editing. **Carlo Rinaldi:** Conceptualization; resources; data curation; formal analysis; funding acquisition; methodology; writing – original draft; project administration; writing – review and editing.

## Disclosure and competing interests statement

The authors declare that they have no conflict of interest.

## Supporting information



Appendix S1

Expanded View Figures PDF

Table EV1

Table EV2

Table EV3

Dataset EV1

Dataset EV2

Dataset EV3

PDF+

Source Data for Figure 1

Source Data for Figure 2

Source Data for Figure 3

Source Data for Figure 4

## Data Availability

The RNA‐seq datasets produced in this study (Fig 5) are available in the following database: Gene Expression Omnibus (GEO), accession number GSE206400 (https://www.ncbi.nlm.nih.gov/geo/query/acc.cgi?acc=GSE206400).
