# Supplementary material for: PRMT inhibitor promotes SMN2 exon 7 inclusion and synergizes with nusinersen to rescue SMA mice
Source: EMBO Mol Med. 2023 Sep 19;15(11):e17683. doi: 10.15252/emmm.202317683 (PMC10630883; doi:10.15252/emmm.202317683)
Supplement: Supplementary file 1 — Appendix S1 [file EMMM-15-e17683-s014.pdf]

**APPENDIX FIGURES**

**Table of content:**

**Appendix Figure S1.** Uncropped western blots from Figure 2C.
**Appendix Figure S2.** Uncropped western blots from Figure 2E.

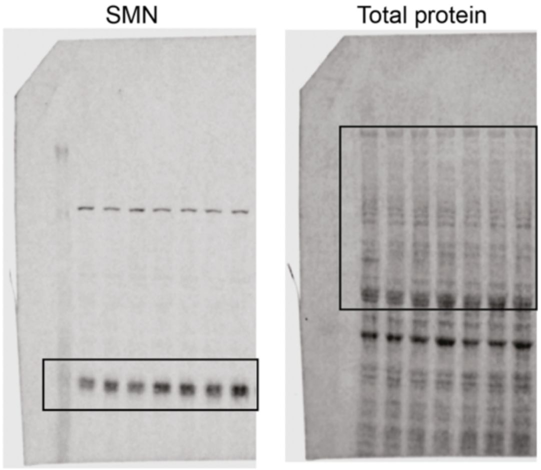


**Appendix Figure S1.** Uncropped western blots from Figure 2C. The black box indicates the signal corresponding to SMN antibody (left) and total protein area used for quantification (left).

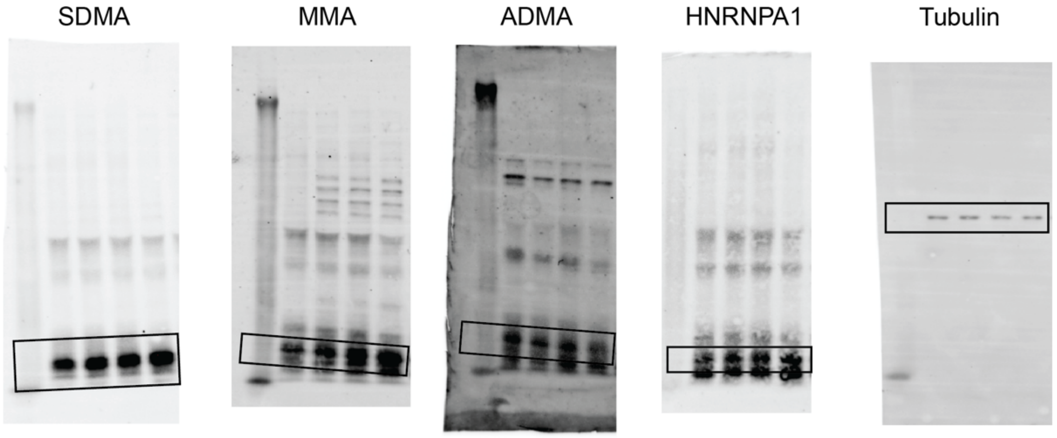


**Appendix Figure S2.** Uncropped western blots from Figure 2E. The black box indicates the signal corresponding to each antibody.
