# Supplementary material for: PRMT inhibitor promotes SMN2 exon 7 inclusion and synergizes with nusinersen to rescue SMA mice
Source: EMBO Mol Med. 2023 Sep 19;15(11):e17683. doi: 10.15252/emmm.202317683 (PMC10630883; doi:10.15252/emmm.202317683)
Supplement: Supplementary file 2 — Expanded View Figures PDF [file EMMM-15-e17683-s011.pdf]

Expanded View Figures

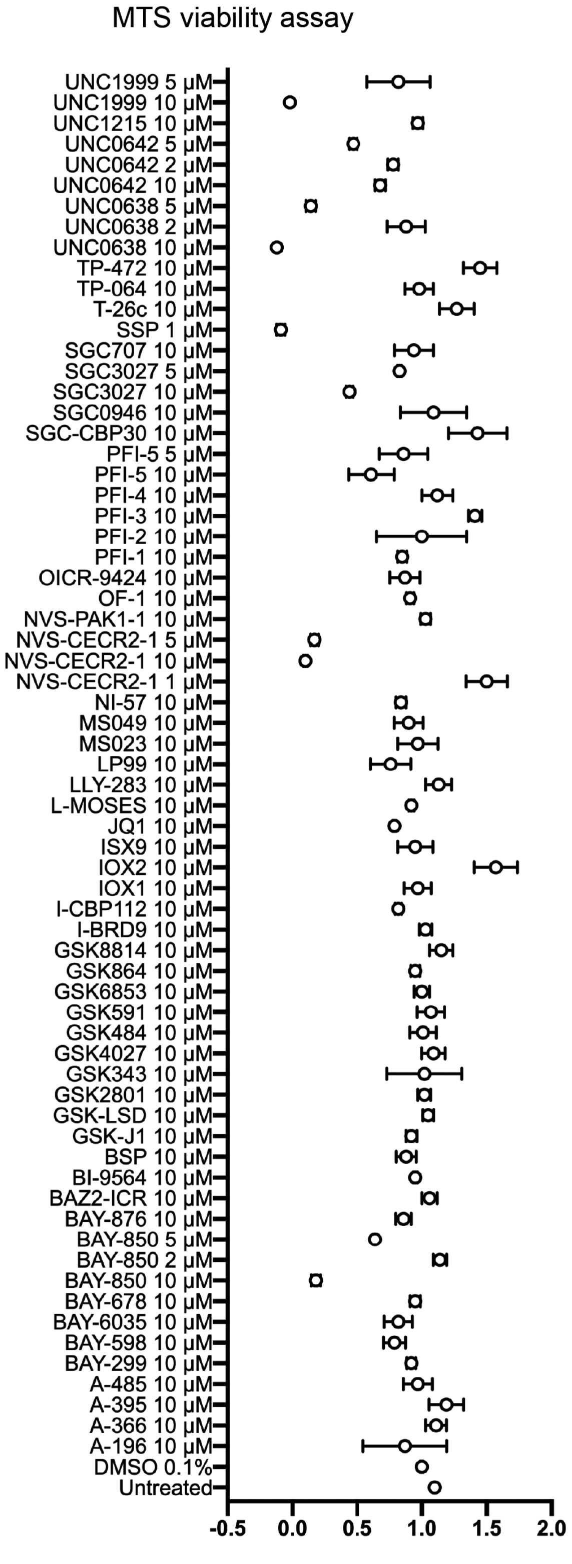

Figure EV1. Effect of epigenetic small molecules on SMA type II patient-derived fibroblast viability.

Viability of cells, assayed by MTS assay, treated with epigenetic small molecules, relative to vehicle-treated cells (0.1% DMSO), normalised to one (n = 2–3). Data are represented as mean ± s.e.m.

© 2023 The Authors

Figure EV2. The increase in *SMN2* exon 7 inclusion by MS023 is specific and does not depend on altered HNRNPA1 levels.

A SMA type II patient-derived fibroblasts were treated with the indicated concentration of MS023 (range: 100 nM–10 μM), (n = 3–4). Cells were harvested for RNA after 48 h incubation. Tot *SMN2* transcript levels relative to *GAPDH* are expressed as fold change compared to untreated SMA fibroblasts, normalised to one. Each dot represents a biological replicate (n = 3–4).

B, C Full-length (FL) SMN2 transcript levels relative to Δ7 SMN2 are expressed as fold change compared to untreated SMA type I and type III fibroblasts, normalised to one. Each dot represents a biological replicate (n = 2–6).

D Western blot showing SMN protein levels upon treatment with increasing MS094 (MS023 negative control) concentrations (top). A representative section of total protein stain, used for protein normalisation, is shown (bottom). The size in kilodalton is indicated on the right.

E Quantification of SMN protein levels relative to total protein is shown. Each dot represents a biological replicate (n = 5).

F Western blot showing vinculin protein (top), HNRNPA1 (middle), and histone 3 (bottom) levels upon treatment with increasing MS023 concentrations and in the cytoplasm (C) and nucleus (N). The size in kilodalton is indicated on the right.

G Quantification of HNRNPA1 protein levels relative to total protein is shown. Each dot represents a biological replicate (n = 4).

Data information: (A), (B), (C), (E), and (G), Data are represented as mean ± s.e.m. and compared with a one-way ANOVA test with multiple comparisons (*$P \leq 0.05$; **$P \leq 0.001$).

 © 2023 The Authors

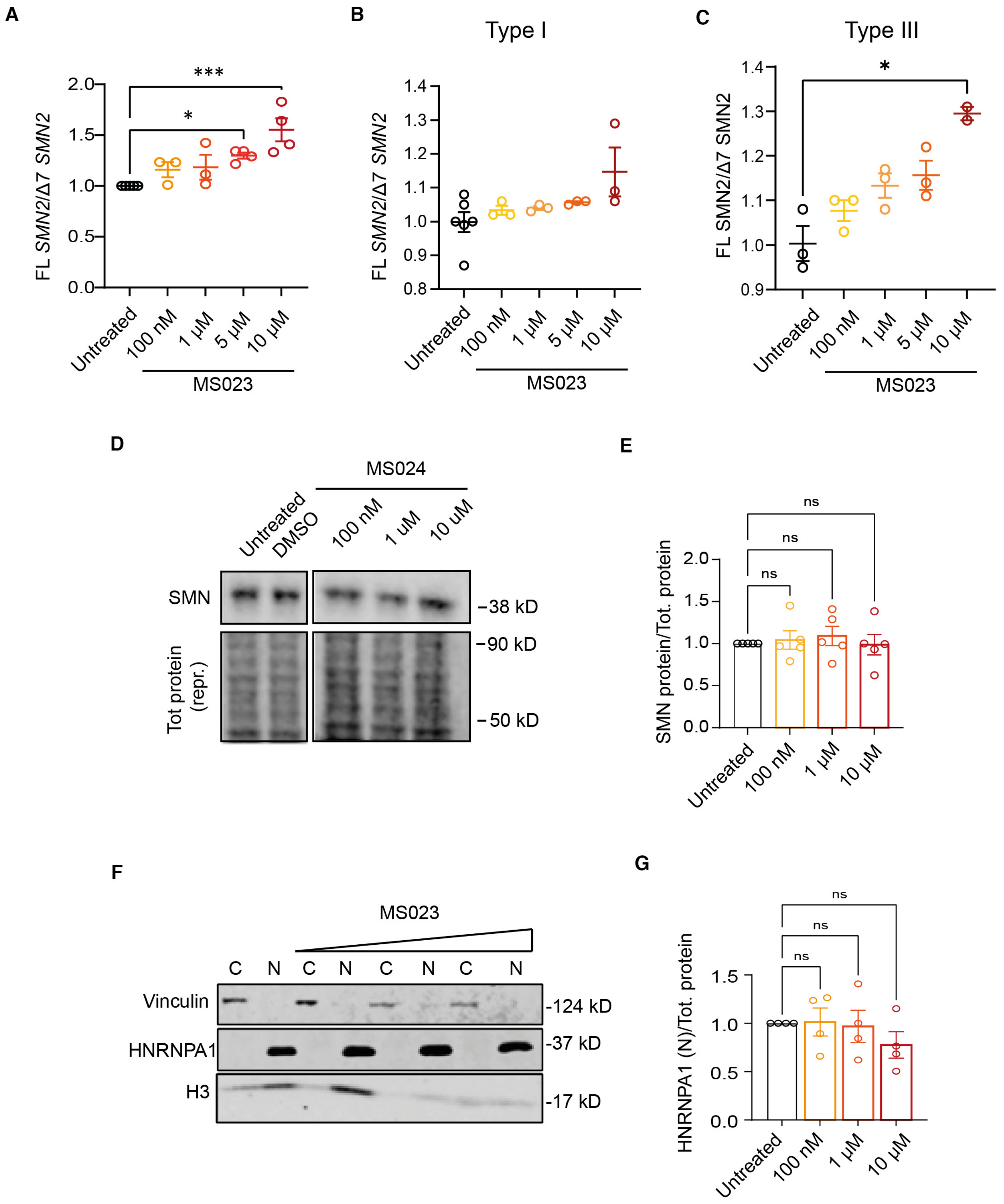
A
B
Type I
C
Type III
FL SMN2/Δ7 SMN2
Untreated
100 nM
1 µM
5 µM
10 µM
MS023
D
MS024
Untreated
DMSO
100 nM
1 uM
10 uM
SMN
Tot protein (repr.)
-38 kD
-90 kD
-50 kD
E
SMN protein/Tot. protein
ns
F
MS023
C
N
Vinculin
HNRNPA1
H3
-124 kD
-37 kD
-17 kD
G
HNRNPA1 (N)/Tot. protein

Figure EV2.

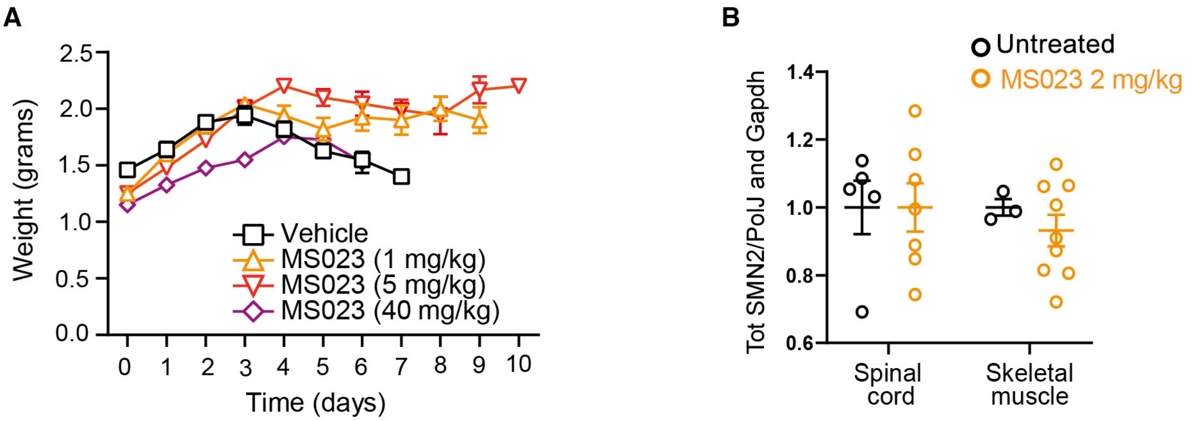

Figure EV3. Oral administration of MS023 improves the phenotype of SMA mice.

A Body weights of untreated (n = 12), MS023- (1 mg/kg: n = 10; 5 mg/kg: n = 9; 40 mg/kg: n = 4), or vehicle-treated (n = 5) SMA mice from postnatal day 0 are shown.

B Tot *SMN2* transcript levels relative to *PolJ* and *Gapdh* in spinal cord and skeletal muscle of treated SMA mice compared to vehicle-treated SMA mice, normalised to one. Each dot represents a biological replicate (n = 10–12).

Data information: (A, B) Data are represented as mean ± s.e.m. and compared with a one-way ANOVA test with multiple comparisons.

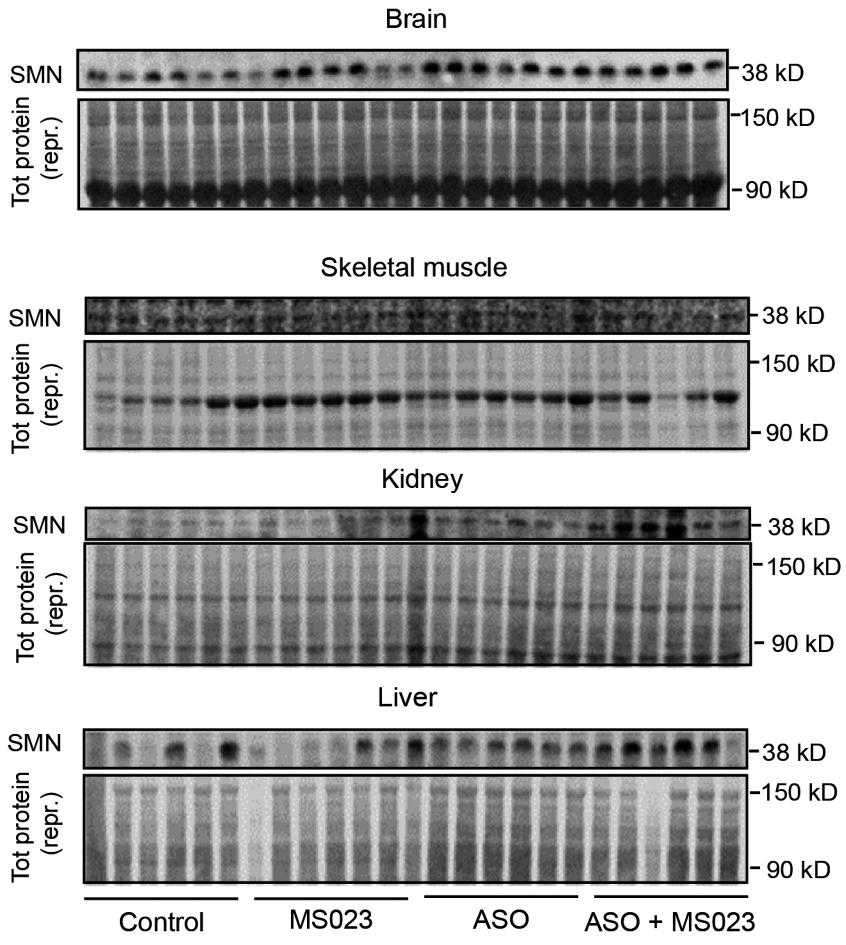

Figure EV4. Combinatorial treatment with MS023 and ASO exerts synergistic effects in SMA mice.

Western blot showing SMN protein levels following the indicated treatments in the brain, skeletal muscle, kidney, and liver of SMA mice (top). A representative section of total protein stain, used for protein normalisation, is also shown (bottom). The size in kilodalton is indicated on the right.

 © 2023 The Authors

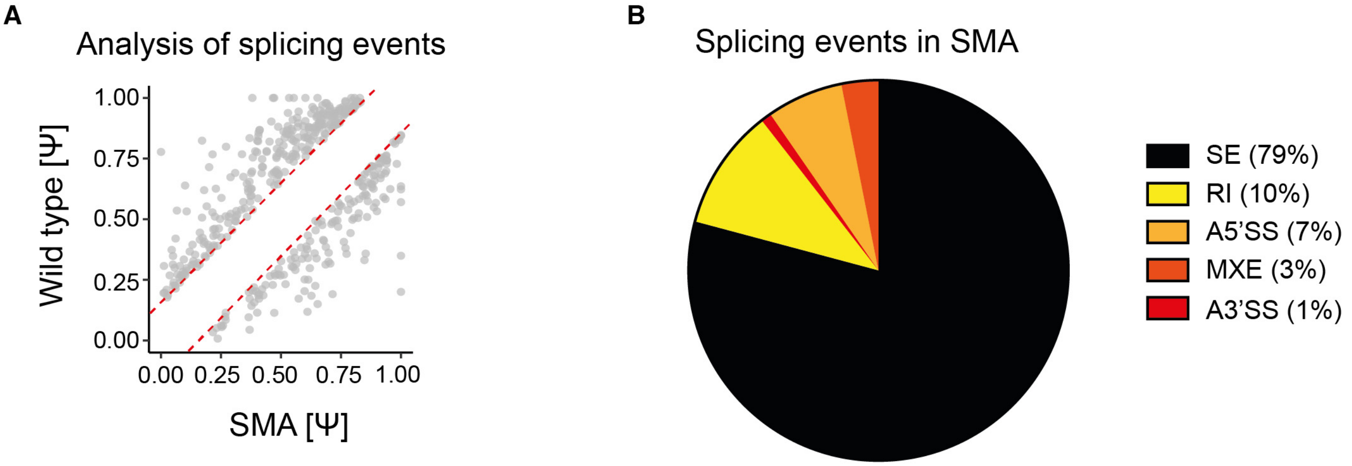

Figure EV5. Altered splicing events in SMA.

A Plot charts show the distribution of 446 aberrant splicing events (Ψ) in SMA mice relative to wild-type littermates. The red dotted lines mark the ±15% normalisation range.

B Pie chart showing the proportion of altered splicing events in the spinal cord of SMA mice compared to wild type (A3′SS, alternative 3′ splice site; A5′SS, alternative 5′ splice site; MXE, mutually exclusive exon; RI, intron retention; SE, skipped exon).

© 2023 The Authors
