# Supplementary material for: PRMT inhibitor promotes SMN2 exon 7 inclusion and synergizes with nusinersen to rescue SMA mice
Source: EMBO Mol Med. 2023 Sep 19;15(11):e17683. doi: 10.15252/emmm.202317683 (PMC10630883; doi:10.15252/emmm.202317683)
Supplement: Supplementary file 9 — PDF+ [file EMMM-15-e17683-s001.pdf]

SOURCE DATA TRANSPARENT PROCESS OPEN ACCESS

EMBO Molecular Medicine

Article

PRMT inhibitor promotes *SMN2* exon 7 inclusion and synergizes with nusinersen to rescue SMA mice

Anna J Kordala[1,2,3], Jessica Stoodley[2,3], Nina Ahlskog[2,3], Muhammad Hanifi[2], Antonio Garcia Guerra[2,3], Amarjit Bhomra[2,3], Wooi Fang Lim[2,3], Lyndsay M Murray[4,5], Kevin Talbot[6,7], Suzan M Hammond[2], Matthew JA Wood[2,3,8] & Carlo Rinaldi[2,3,8,*]

Abstract

Spinal muscular atrophy (SMA) is a leading genetic cause of infant mortality. The advent of approved treatments for this devastating condition has significantly changed SMA patients' life expectancy and quality of life. Nevertheless, these are not without limitations, and research efforts are underway to develop new approaches for improved and long-lasting benefits for patients. Protein arginine methyltransferases (PRMTs) are emerging as druggable epigenetic targets, with several small-molecule PRMT inhibitors already in clinical trials. From a screen of epigenetic molecules, we have identified MS023, a potent and selective type I PRMT inhibitor able to promote *SMN2* exon 7 inclusion in preclinical SMA models. Treatment of SMA mice with MS023 results in amelioration of the disease phenotype, with strong synergistic amplification of the positive effect when delivered in combination with the antisense oligonucleotide nusinersen. Moreover, transcriptomic analysis revealed that MS023 treatment has minimal off-target effects, and the added benefit is mainly due to targeting neuroinflammation. Our study warrants further clinical investigation of PRMT inhibition both as a stand-alone and add-on therapy for SMA.

Keywords nusinersen; PRMT inhibitor; small molecule; spinal muscular atrophy
Subject Categories Musculoskeletal System; Neuroscience; Pharmacology & Drug Discovery
DOI 10.15252/emmm.202317683 | Received 7 March 2023 | Revised 31 August 2023 | Accepted 1 September 2023 | Published online 19 September 2023
EMBO Mol Med (2023) 15: e17683

Introduction

Spinal muscular atrophy (SMA) is a genetic neuromuscular condition affecting 1:8,000–10,000 live births (Pearn, 1978) and, to this date, a leading inherited cause of infant mortality worldwide (Crawford & Pardo, 1996). SMA is caused by inactivating mutations, mainly homozygous deletions, in the survival motor neuron 1 (*SMN1*) gene on chromosome 5 (Lefebvre *et al*, 1995). The encoded SMN protein is ubiquitously expressed, localising both to the cytoplasmic and nuclear compartments within the cell, where it exerts numerous essential functions, including biogenesis of small nuclear ribonucleoproteins (snRNPs), its most widely studied function (Friesen *et al*, 2001a; Pellizzoni *et al*, 2002; Zhang *et al*, 2011), 3′ processing of histone mRNAs (Tisdale *et al*, 2013), control of transcription (Strasswimmer *et al*, 1999;Suraweera *et al*, 2009; Yanling Zhao *et al*, 2016), R-loop resolution (Suraweera *et al*, 2009; Yanling Zhao *et al*, 2016), RNA trafficking (Rossoll *et al*, 2003; Piazzon *et al*, 2008; Tadesse *et al*, 2008; Akten *et al*, 2011; Fallini *et al*, 2011, 2014, 2016; Hubers *et al*, 2011; Rage *et al*, 2013) and pre-mRNA splicing (Pellizzoni *et al*, 1998; Charroux *et al*, 1999; Shafey *et al*, 2010; Makarov *et al*, 2012). The *SMN2* gene is a centromeric copy of telomeric *SMN1*, with a critical C to T substitution in position 6 of exon 7, which creates an exonic splicing silencer (ESS) and is recognised by a splicing factor, HNRNPA1 (Kashima & Manley, 2003; Kashima *et al*, 2007a). As a consequence, the *SMN2* gene mainly encodes a shorter and rapidly degraded SMN isoform lacking exon 7 (Δ7 SMN), with only 10–15% of *SMN2* transcripts still capable of generating a full-length SMN protein (Lorson *et al*, 1999; Monani *et al*, 1999). The number of *SMN2* copies varies in the general population and is the main modifier of disease severity identified so far, with a higher number of copies being associated with a milder SMA phenotype (McAndrew *et al*, 1997; Feldkötter *et al*, 2002). Depending on the age of onset and motor milestones achieved, SMA has been divided into four clinical types (I–IV) (Munsat & Davies, 1992), with type I SMA infants showing symptoms before 6 months of age and never gaining the ability to sit unaided.

Loss of SMN leads to degeneration of lower α-motor neurons by molecular mechanisms, which are not fully understood. Other

1 Department of Physiology Anatomy and Genetics, University of Oxford, Oxford, UK
2 Department of Paediatrics, University of Oxford, Oxford, UK
3 Institute of Developmental and Regenerative Medicine (IDRM), Oxford, UK
4 Centre for Discovery Brain Sciences, College of Medicine and Veterinary Medicine, University of Edinburgh, Edinburgh, UK
5 Euan McDonald Centre for Motor Neuron Disease Research, University of Edinburgh, Edinburgh, UK
6 Nuffield Department of Clinical Neurosciences, John Radcliffe Hospital, University of Oxford, Oxford, UK
7 Kavli Institute for Nanoscience Discovery, University of Oxford, Oxford, UK
8 MDUK Oxford Neuromuscular Centre, Oxford, UK
*Corresponding author. Tel: +441865272148; E-mail: carlo.rinaldi@idrm.ox.c.uk

© 2023 The Authors. Published under the terms of the CC BY 4.0 license

neuronal and non-neuronal cell populations are affected in SMA patients and include sensory neurons (Rudnik-Schöneborn *et al*, 2003; Jablonka *et al*, 2006; Mentis *et al*, 2011; Gogliotti *et al*, 2012; Martinez *et al*, 2012), skeletal muscle (Arnold *et al*, 2004; Martínez-Hernández *et al*, 2009; Kim *et al*, 2020) heart and vasculature (Araujo *et al*, 2009; Bevan *et al*, 2010; Heier *et al*, 2010; Rudnik-Schöneborn *et al*, 2010; Somers *et al*, 2016; Lipnick *et al*, 2019), liver (Crawford *et al*, 1999; Deguise *et al*, 2019), and pancreas (Bowerman *et al*, 2012), supporting the notion that SMA is a multi-systemic condition.

In the last decade, the advent of successful therapeutic approaches, combined with improvements in standards of care, has effectively changed the course of this disease, significantly slowing down the progression of all SMA types (Harding *et al*, 2015; Mercuri *et al*, 2020, 2022). One of such approved strategies entails intrathecal injections of nusinersen, an antisense oligonucleotide (ASO) targeting intronic splicing silencer N1 (ISS-N1), promoting *SMN2* exon 7 inclusion and increasing levels of full-length SMN protein. Concomitantly, these treatment opportunities pose new challenges, including their yet-to-be determined long-term effects, the rise of new phenotypes in treated patients, which is particularly relevant for approaches such as nusinersen, solely targeting the central nervous system (CNS), the need for repeated invasive administrations, and high costs. Altogether, these considerations highlight the urgency for development of therapeutic combinations to address these important limitations and to provide additional benefit to patients.

Epigenetic regulation of gene expression, which involves covalent and sequence-specific modifications of histone and non-histone proteins, is a dynamic and reversible process that establishes normal cellular phenotypes and, when dysregulated, contributes to a wide range of human diseases, including SMA (Allis *et al*, 2007; Hauke *et al*, 2009; Portela & Esteller, 2010; Zheleznyakova *et al*, 2013; Murray *et al*, 2015; Cao *et al*, 2016). In recent years, key protein families that mediate epigenetic signalling through the acetylation and methylation of histones and non-histone proteins, including histone deacetylases (HDACs), protein methyltransferases (PRMTs), histone lysine methyltransferases (KMTs) and demethylases (KDMs), and bromodomain-containing proteins (BRD) have emerged as attractive druggable targets using small molecules, due to the dynamic nature of disease-associated epigenetic states (Arrowsmith *et al*, 2012). Several small-molecule inhibitors of histone deacetylases have been tested in SMA models (Mohseni *et al*, 2013), but their lack of specificity, low potency, and poor understanding of their mechanisms of action have significantly limited their translation into the clinic (Mercuri *et al*, 2007; Kissel *et al*, 2014; Krosschell *et al*, 2018). A recent study has shown that nusinersen, while promoting exon 7 inclusion, also induces a silencing histone mark H3K9me2 on *SMN2* gene, creating a roadblock to RNA polymerase II elongation. Histone deacetylase inhibitor—valproic acid, counteracts chromatin effects of the ASO, resulting in higher exon 7 inclusion upon combined treatment compared to nusinersen alone (Marasco *et al*, 2022). The primary aim of this project is to identify next generation small molecules targeting epigenetic proteins able to increase SMN protein and evaluate their therapeutic potential in SMA animal models alone and as an add-on treatment.

Results

Epigenetic screening of *SMN2* modulators

In order to identify small molecules that selectively modulate *SMN2* pre-mRNA splicing to include exon 7, we performed a cell-based screen in SMA type II-patient derived fibroblasts carrying three copies of the *SMN2* gene, using a collection of 54 chemical probes from the Structural Genomics Consortium (SGC) collection (https://www.thesgc.org/chemical-probes) (Scheer *et al*, 2019; Wu *et al*, 2019) (Table EV1). This unique library includes compounds targeting key epigenetic regulatory proteins with a high degree of potency and selectivity, and a favourable therapeutic index (Ackloo *et al*, 2017). The maximum non-toxic concentrations for each compound, established by a viability assay in these cells, were used in the screen (Fig EV1). Of the 54 molecules, only one molecule, selective type I PRMT inhibitor MS023, was able to promote exon 7 inclusion in *SMN2* pre-mRNA, without affecting total *SMN2* mRNA levels (Fig 1A and B). MS023 treatment in these fibroblasts also increased SMN protein levels up to 1.6-fold, as determined by Western blot analysis (Fig 1C). Notably, treatment with PRMT5 inhibitors: LLY-283 and GSK591 resulted in reduction of exon 7 inclusion in *SMN2* pre-mRNA and in SMN protein, respectively (Fig 1A–C), overall suggesting that protein arginine asymmetric and symmetric dimethylation by different families of PRMTs exert opposite effects on SMN regulation. Other compounds, including bromodomain inhibitors (BAY-299, BI-9564, JQ1) and lysine demethylase inhibitors (GSK-J1, GSK-LSD), also elicited a ≥ 1.5-fold increase in SMN protein without affecting mRNA levels, hinting at a direct or indirect effect on SMN protein regulation (Fig 1A–C). PRMTs are involved in several critical biological functions (Blanc & Richard, 2017), and represent a promising therapeutic target for many human diseases from cancer to neurodegeneration, with at least eight PRMT inhibitors attaining clinical trial testing in human cancers (Yang & Bedford, 2013; Guccione & Richard, 2019; Hwang *et al*, 2021). Altogether, the direct effect of PRMT type I inhibition on *SMN2* exon 7 inclusion and the potential for clinical impact of this class of molecules, prompted us to further investigate MS023 as a therapeutic agent for SMA.

Type I PRMT inhibition promotes exon 7 inclusion in SMN2 pre-mRNA by decreasing HNRNPA1 binding

MS023 is a recently identified potent and selective inhibitor of type I PRMTs harbouring an ethylenediamine group, a critical moiety for its activity (Fig 2A; Eram *et al*, 2016). Treatment of type II SMA fibroblasts with MS023 led to a dose-dependent increase in both *SMN2* exon 7 inclusion and protein levels (Figs 2B–D and EV2A and Appendix Fig S1), and this effect was confirmed also in fibroblasts from severe (type I) and mild (type III) SMA patients (Fig EV2B and C). No change in SMN levels was observed upon treatment with MS094 (Fig EV2D and E), an inactive MS023 analogue where the terminal primary amino group in the ethylenediamine group is replaced with a hydroxyl group (Eram *et al*, 2016), further confirming its dependency on PRMT activity. In order to identify the PRMT substrate mediating the effect of MS023 on *SMN2*, we interrogated a recently published dataset of the arginine methyl proteome

© 2023 The Authors

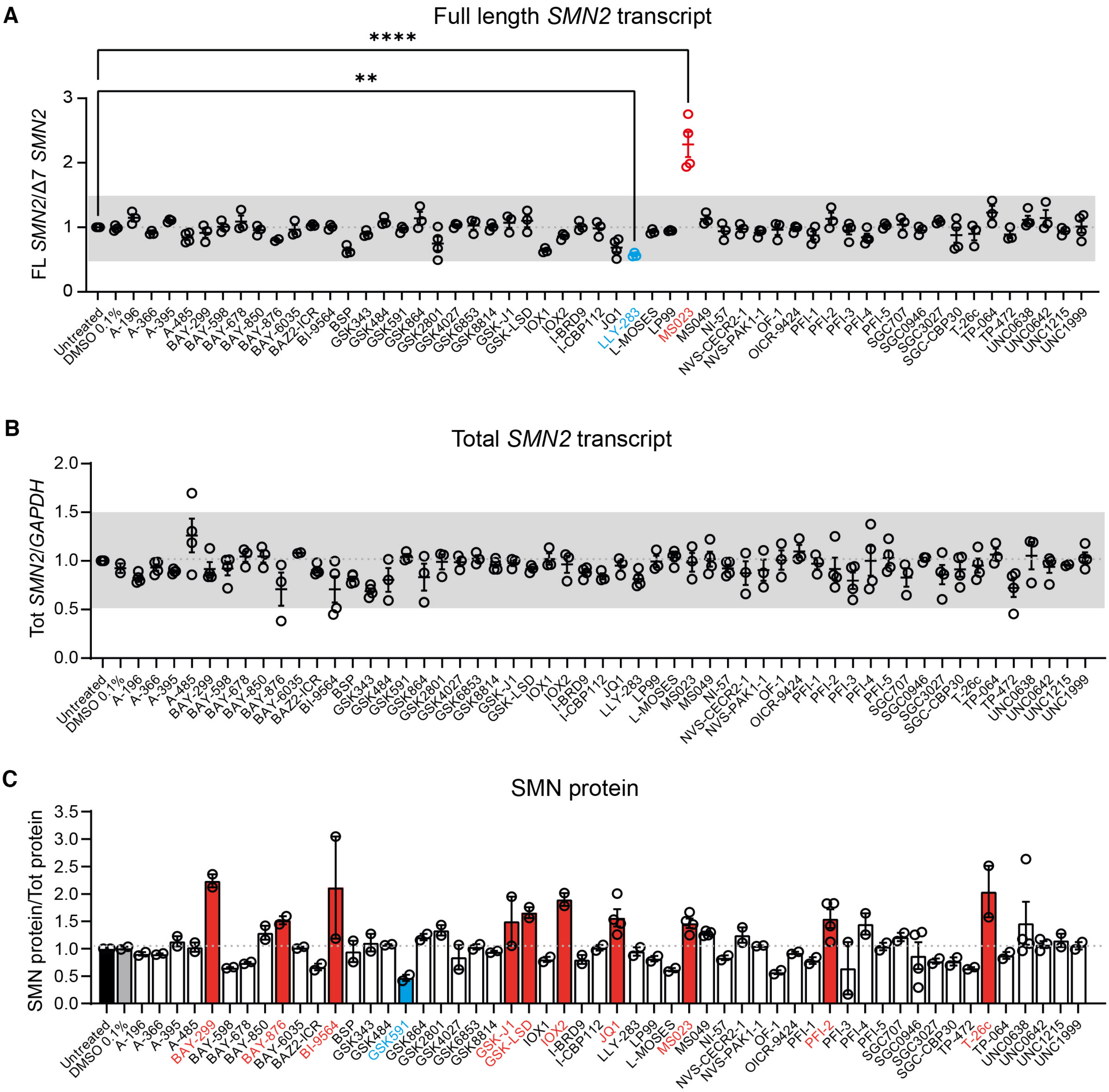

Figure 1. Screening of epigenetic small molecules in SMA fibroblasts.

A–C SMA type II patient-derived fibroblasts were treated with the indicated small molecule at the appropriate maximum tolerated dose (range: 1–10 μM) (n = 3–4). Cells were harvested for RNA (A, B) and protein (C) quantification 48 and 72 h post-treatment, respectively. (A) Full-length (FL) *SMN2* transcript levels relative to Δ7 *SMN2* are expressed as fold change compared to untreated SMA fibroblasts, normalised to one (dashed line). FL *SMN2*/Δ7 *SMN2* ratios were significantly increased by MS023 and decreased by LLY-283 treatment. (B) Tot *SMN2* transcript levels relative to *GAPDH* are expressed as fold change compared to untreated SMA fibroblasts, normalised to one (dashed line). (A, B) Each dot represents a biological replicate (n = 3–4). The grey bar indicates values within the 0.5–1.5 range. (C) SMN protein levels relative to total protein are expressed as fold change compared to untreated SMA fibroblasts, normalised to one (dashed line). Each dot represents a biological replicate (n = 2–4). Values ≥ 1.5 and ≤ 0.5 are depicted in red and blue, respectively. (A–C) Data are represented as mean ± standard error of the mean (s.e.m.) and compared with a one-way ANOVA test with multiple comparisons (**$P \le 0.01$; ****$P \le 0.0001$).

Source data are available online for this figure.

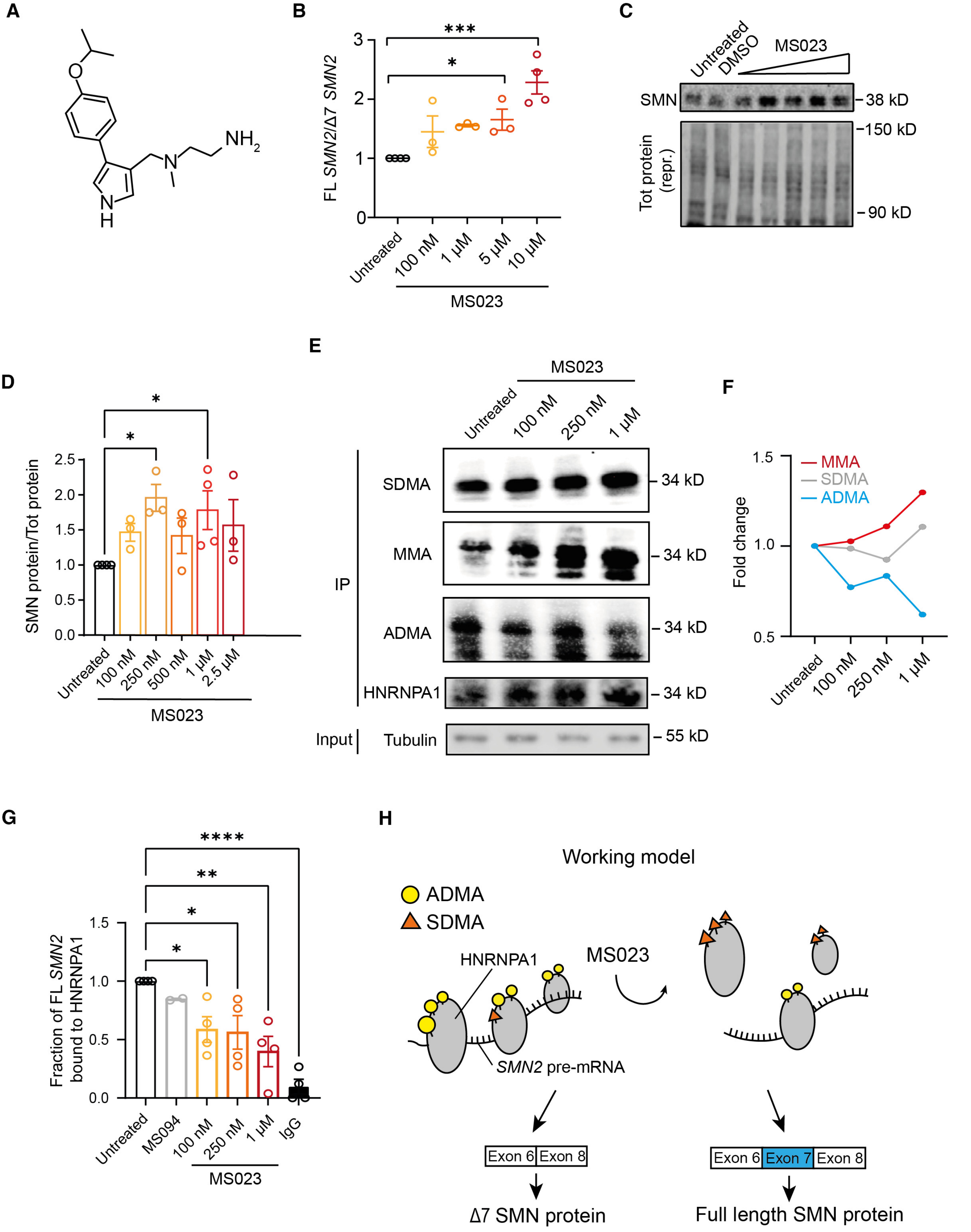

Figure 2.

© 2023 The Authors

◀ **Figure 2. MS023 promotes exon 7 inclusion into *SMN2* transcript via HNRNPA1 binding inhibition.**

A Chemical structure depiction of MS023 (PubChem CID: 92136227).

B–D SMA type II patient-derived fibroblasts were treated with the indicated concentration of MS023 (range: 100 nM–10 μM), (n = 3–4). Cells were harvested for RNA (B) and protein (C, D) quantification 48 and 72 h post treatment, respectively (B). Full-length (FL) *SMN2* transcript levels relative to Δ7 *SMN2* are expressed as fold change compared to untreated SMA fibroblasts, normalised to one. Each dot represents a biological replicate (n = 3–4). (C) Western blot showing SMN protein levels upon treatment with increasing MS023 concentrations (top). A representative section of total protein stain, used for protein normalisation, is shown (bottom). The size in kilodalton is indicated on the right. (D) Quantification of SMN protein levels relative to total protein is shown. Each dot represents a biological replicate (n = 3–4).

E Arginine monomethylation (MMA) and symmetric dimethylation (SDMA) of HNRNPA1 are increased and asymmetric dimethylation (ADMA) of HNRNPA1 is decreased upon MS023 treatment, as shown by HNRNPA1 immunoprecipitation, followed by a Western blot. SMA type II patient-derived fibroblasts were treated with the indicated concentration of MS023 (range: 100 nM–1 μM). HNRNPA1 was immunoprecipitated and Western blots were performed using anti-MMA, anti-SDMA, anti-ADMA, anti-HNRNPA1, and anti-tubulin antibodies.

F Quantification of arginine methylation changes normalised to total HNRNPA1 is shown.

G Ratio of FL *SMN2* transcripts bound to HNRNPA1 protein and relative to *GAPDH* was assayed using crosslinking immunoprecipitation (CLIP) in SMA patient fibroblasts treated with increasing concentrations of MS023. IgG antibody and MS094 were used as controls, MS094 is a negative control for MS023 lacking PRMT inhibition property. Each dot represents a biological replicate (n = 2–4).

H Representation of the mechanism of exon 7 inclusion into the *SMN2* transcript by MS023. Blue circles, purple squares, and red triangles represent asymmetric dimethylarginine (ADMA), symmetric dimethylarginine (SDMA), monomethylarginine (MMA), respectively.

Data information: (B), (D), and (G) Data are represented as mean ± s.e.m. and compared with a one-way ANOVA test with multiple comparisons (*$P \le 0.05$; **$P \le 0.01$; ***$P \le 0.001$; ****$P \le 0.0001$).
Source data are available online for this figure.

in human NB4 cells upon treatment with MS023 (Fong *et al*, 2019). Out of 72 responsive targets, we noted that MS023 determined largely a downregulation of asymmetric dimethylarginine (ADMA) and an increase in monomethylarginine (MMA) sites in the heterogeneous nuclear ribonucleoprotein A1 (HNRNPA1), alongside changing the methylation pattern of other splicing factors implicated in *SMN2* splicing regulation (Fong *et al*, 2019). HNRNPA1 directly binds *SMN2* pre-mRNA across multiple sites and is a well-established negative regulator of exon 7 splicing (Cartegni *et al*, 2006; Kashima *et al*, 2007b; Bose *et al*, 2008; Chen *et al*, 2008; Hua *et al*, 2008; Koed Doktor *et al*, 2011; Xiao *et al*, 2012; Singh *et al*, 2013). Given that PRMT-regulated methylation regulates binding of RNA-binding proteins to RNAs (Blanc & Richard, 2017), we hypothesised that MS023-induced ADMA-to-MMA switch in HNRNPA1 methylation affects its binding to *SMN2* pre-mRNA, resulting in exon 7 inclusion. In order to test this hypothesis, we treated SMA fibroblasts with increasing concentrations of MS023 and confirmed a dose-responsive increase in both symmetric dimethylarginine (SDMA) and MMA methylation and a concomitant reduction in ADMA levels of HNRNPA1 protein (Fig 2E and F; Appendix Fig S2). Concomitantly, we observed a dose-responsive reduction of the fraction of FL *SMN2* transcripts bound to HNRNPA1, as determined by a crosslinking and immunoprecipitation (CLIP) assay (Fig 2G), which validated our working model (Fig 2H). Treatment with MS023 did not change HNRNPA1 levels nor its subcellular localisation, further suggesting that the treatment-induced change in arginine methylation specifically affects HNRNPA1 binding affinity with its RNA target (Fig EV2F and G). Taken together, these findings indicate that MS023 promotes *SMN2* exon 7 inclusion via decreased binding of HNRNPA1 to *SMN2* pre-mRNA.

Oral administration of MS023 improves the phenotype of SMA mice alone and in synergy with nusinersen

We next evaluated the efficacy and tolerability of MS023 treatment *in vivo* in a severe preclinical mouse model of SMA (Hsieh-Li *et al*, 2000). These mice, which lack the mouse *Smn* gene and only carry a single copy of the human *SMN2* gene ($Smn^{-/-}$; $SMN2^{+/-}$), display a phenotype with weight loss and reduced motor activity starting at postnatal day 5 (P5) and typically reach a humane end point by P9. Daily oral administration of MS023 or vehicle (0.5% DMSO in 0.9% saline solution) was performed in SMA mice from P0 until reaching a humane end point (Fig 3A). We tested a range of doses (1, 2, 5, and 40 mg/kg) and found that treatment with both 2 and 5 mg/kg resulted in a significant increase in survival, with the 2 mg/kg dose achieving the best effect (median: 10 days) compared to vehicle-treated mice (median: 6 days; $P < 0.0001$) (Fig 3B). Mice treated with this regimen also showed an improvement in disease-associated weight loss (Fig 3C). No further amelioration was observed with 40 mg/kg MS023, suggesting that with this dose the therapeutic window has been surpassed (Figs 3B and EV3A). Notably, we detected an increase in full-length (FL) *SMN2* transcript in skeletal muscle and SMN protein levels in the both spinal cord and skeletal muscle, the tissues mostly affected in the disease (Mercuri *et al*, 2022), of SMA mice treated with 2 mg/kg MS023 (Figs 3D–F and EV3B). Since the mechanism of action of the 2′-O-methoxyethyl phosphorothioate-modified drug nusinersen, a currently approved ASO therapy for SMA patients, consists of promoting exon 7 inclusion into *SMN2* pre-mRNA by blocking the recruitment of HNRNP splicing repressors at the ISS-N1 site (Chiriboga *et al*, 2016; Finkel *et al*, 2016, 2017; Haché *et al*, 2016; Mercuri *et al*, 2018), we postulated that a combinatorial treatment with MS023 would synergistically lead to improved therapeutic benefit and allow for a cost-effective ASO dosing regimen in SMA. In order to test this hypothesis, at P0 SMA mice were treated with a single subcutaneous administration of 30 mg/kg nusinersen, a suboptimal dose sufficient to slightly extend the SMA mice life span (Hammond *et al*, 2016), alone or in combination with daily oral administration of 2 mg/kg MS023 from P1 to P6 (Fig 4A). Analysis of tissues collected at P7 showed that the combinatorial treatment was able to further enhance exon 7 inclusion in *SMN2* pre-mRNA (Fig 4B) and increase SMN protein levels (Figs 4C and D and EV4), both in the CNS and peripheral tissues. A further follow-up study designed to assess the effect on survival (Fig 4E) revealed that the combinatorial treatment of nusinersen and MS023 dramatically prolonged the lifespan (median: 97.5 days) compared to nusinersen

© 2023 The Authors

alone (median: 19.5; $P = 0.02$; Fig 4F) and improved the body weight of SMA mice (Fig 4G). Overall, these results suggest that oral administration of MS023 synergises with nusinersen to provide a therapeutic benefit in SMA.

Molecular signature of the combinatorial treatment

In order to understand the molecular underpinnings of the added benefit provided by the combinatorial treatment, we performed a

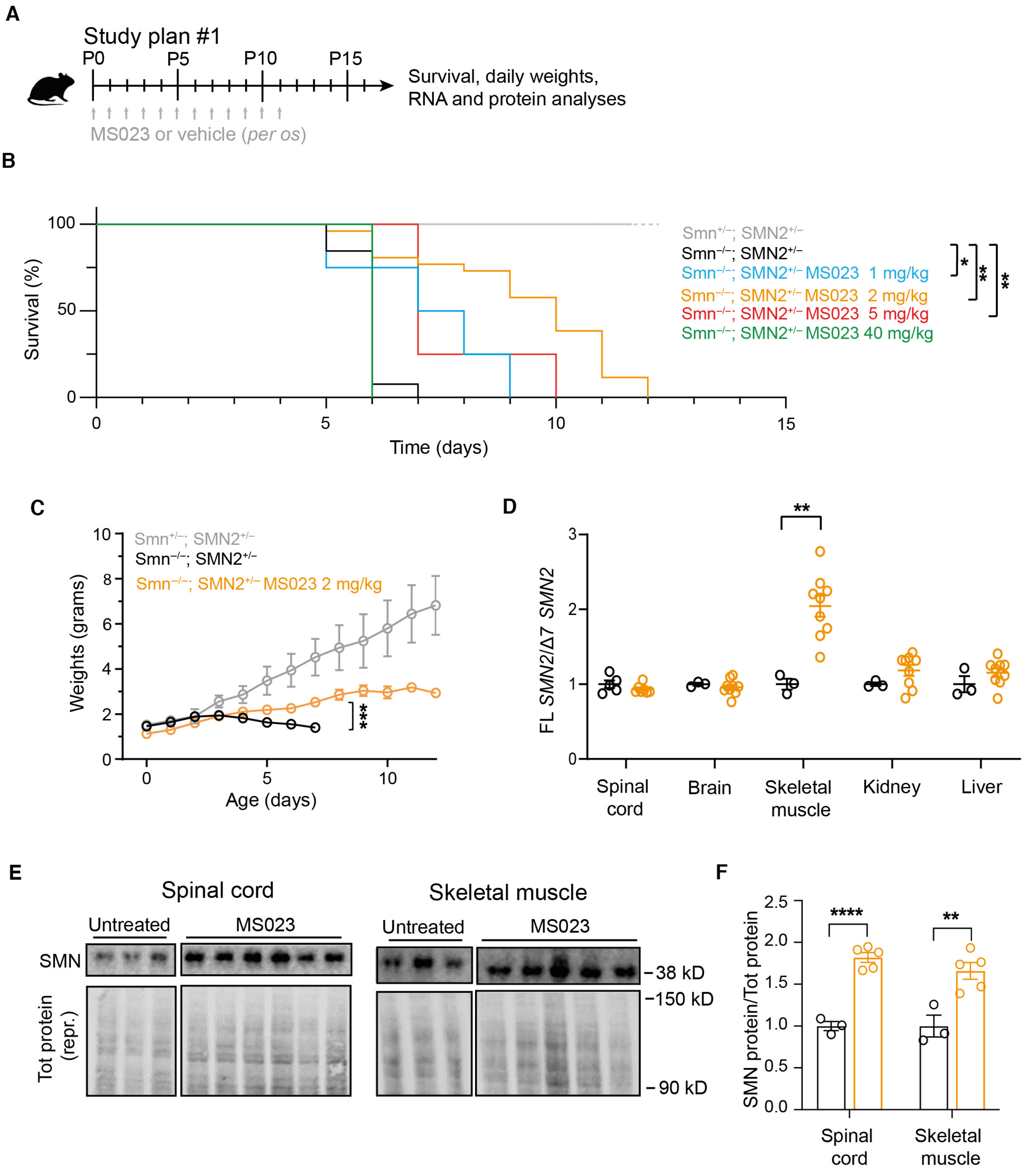

Figure 3.

© 2023 The Authors

Figure 3. Oral administration of MS023 improves the phenotype of SMA mice.

A Diagram of the study design: SMA mice were treated daily with oral administrations of MS023 or vehicle (0.5% DMSO in saline) from postnatal day 0 (P0) using a Hamilton syringe.

B Kaplan-Meier survival estimation of unaffected mice (Smn$^{+/-}$; SMN2$^{+/-}$) and SMA affected mice (Smn$^{-/-}$; SMN2$^{+/-}$) treated with vehicle (n = 14), 1 mg/kg MS023 (n = 4), 2 mg/kg MS023 (n = 26), 5 mg/kg MS023 (n = 4), or 40 mg/kg MS023 (n = 4).

C Body weights of unaffected (n = 12), MS023-treated (n = 26), and vehicle-treated (n = 12) SMA mice from postnatal day 0 are shown.

D FL *SMN2* transcript levels relative to Δ7 *SMN2* in the spinal cord and skeletal muscle of treated mice compared to vehicle-treated mice, normalised to one. Each dot represents a biological replicate (n = 3–9).

E Western blot showing SMN protein levels upon MS023 treatment in spinal cords and skeletal muscles of treated mice (top). A representative section of total protein stain, used for protein normalisation, is also shown (bottom). The size in kilodalton is indicated on the right.

F Quantification of SMN protein levels in the spinal cord and skeletal muscle relative to total protein is shown. Each dot represents a biological replicate (n = 3–8).

Data information: (B–D), and (F), Data are compared with Mantel-Cox test (*$P \leq 0.05$; **$P \leq 0.01$) (B) or represented as mean ± s.e.m. and compared with a one-way ANOVA test with multiple comparisons (**$P \leq 0.01$; ***$P \leq 0.001$, ****$P \leq 0.0001$) (C, D, F).
Source data are available online for this figure.

bulk transcriptomic analysis in the spinal cords of symptomatic (P7) SMA (Smn$^{-/-}$; SMN2$^{+/-}$) mice treated with MS023 only, nusinersen only, and MS023 in combination with nusinersen, compared to untreated SMA and unaffected (Smn$^{+/-}$; SMN2$^{+/-}$) mice, following the treatment paradigm described above (Fig 4A). Out of a total of 5,509 significantly dysregulated transcripts in SMA ($P < 0.05$, false discovery rate < 0.05), treatment with MS023 only, nusinersen only, and MS023 in combination with nusinersen resulted in the correction of 4,270 (77.5%), 4,747 (86.2%), and 4,901 (88.9%) targets, respectively (Dataset EV1). Combinatorial treatment was able to exclusively rescue 192/2874 (6.7%) of the upregulated genes and 167/2635 (6.3%) of the downregulated genes that were not corrected upon treatment with nusinersen alone (Fig 5A; Dataset EV2). We generated a heatmap of the top significant genes ($P < 0.01$) of this category (Fig 5B) and performed a hallmark analysis to depict and identify the changes that could explain the beneficial effects of the combinatorial treatment (Fig 5C and D). TNF-α signalling, together with several other immune-related pathways, including interferon response and complement activation, were highly enriched, overall suggesting that targeting neuroinflammation for therapy is key to achieving a beneficial effect in SMA. Interestingly, astrocyte dysfunction and chronic microglia activation have been observed early in SMA and other neurological conditions (Eikelenboom *et al*, 2002; Sargsyan *et al*, 2005; Heneka *et al*, 2014; Vukojicic *et al*, 2019), and their contribution to neuronal dysfunction and death is abundantly reported (Mcgivern *et al*, 2013; Rindt *et al*, 2015; Zhou *et al*, 2016; Martin *et al*, 2017). Notably, the combinatorial treatment showed a good safety profile, changing the expression of only 160 off-target genes (i.e. unrelated to SMA) (Dataset EV3). Given the role of SMN in RNA biogenesis and spliceosomal protein assembly (Pellizzoni *et al*, 2001) and the observation of increased mis-splicing events upon SMN depletion (Zhang *et al*, 2008, 2013; Bäumer *et al*, 2009; Huo *et al*, 2014; Custer *et al*, 2016; Doktor *et al*, 2017), we wondered whether the beneficial effect of the combinatorial treatment was due to restoration in the splicing profile. We identified 446 mis-splicing events in the spinal cord of SMA mice, with the vast majority (n = 353, 79%) being skipped exons, in accordance with previous reports (Zhang *et al*, 2008, 2013; Bäumer *et al*, 2009; Huo *et al*, 2014; Custer *et al*, 2016; Doktor *et al*, 2017; Fig EV5A and B). Individual treatments with MS023 or nusinersen were both able to restore the splicing profile almost fully (347/446 and 368/446, respectively), with only 14 unique splicing events corrected exclusively upon the combinatorial treatment (Fig 5E; Table EV2), suggesting that mis-splicing has a low threshold for normalisation in SMA and is a poor predictor of treatment response.

Discussion

Thousands of SMA patients are treated each year with nusinersen, the first SMA treatment to receive FDA and EMA approval in 2016 and 2017, respectively. Despite having enormously changed the life expectancy and quality of life of patients, this approach is not without limitations: it fails to address the peripheral manifestations of the disease, is costly, and the intrathecal administrations require patients' hospitalisation and well-trained clinicians, becoming more troublesome with time as the disease progresses (Mercuri *et al*, 2020). Research efforts are underway to develop both SMN-dependent and SMN-independent therapies for improved and sustained benefit and to clinically evaluate the safety and efficacy of combinatorial approaches in SMA patients (NCT04488133, NCT03032172).

From a screen of potent and highly selective epigenetic probes, we have identified MS023, a potent and selective next-generation type I PRMT inhibitor (Eram *et al*, 2016), able to elevate SMN protein in SMA models at levels comparable to recently clinically approved small molecule risdiplam (Naryshkin *et al*, 2014) and to synergistically amplify the effects of nusinersen. Interestingly, targeting type II PRMTs by selective PRMT5 inhibitors GSK591 and LLY-283 reduced FL *SMN2*, supporting a mechanism of regulation of *SMN2* by this class of enzymes. When assessing the *in vivo* effects of MS023 treatment, we observed a significant increase in SMN protein in the spinal cords of SMA mice, while transcript levels remained unchanged (Fig 3D–F), possibly suggesting that the dose of 2 mg/kg used in this study is at the threshold for correction. Additionally, *SMN2* mRNA levels have been shown to rapidly return to baseline within 32 h after both single and repeated dosing of splicing-inducing small molecules such as risdiplam, while the SMN protein continues to increase (Naryshkin *et al*, 2014), and *SMN2* mRNA expression poorly correlates with protein expression in spinal cord samples from human SMA subjects (Ramos *et al*, 2019). Overall, these data hint at mechanisms other than transcriptional activity or splicing, such as translational efficiency and/or protein stability, that may contribute to SMN protein expression in spinal cord (Ramos *et al*, 2019).

Protein arginine methylation is a prevalent posttranscriptional modification occurring at the nitrogen atoms of the guanidinium

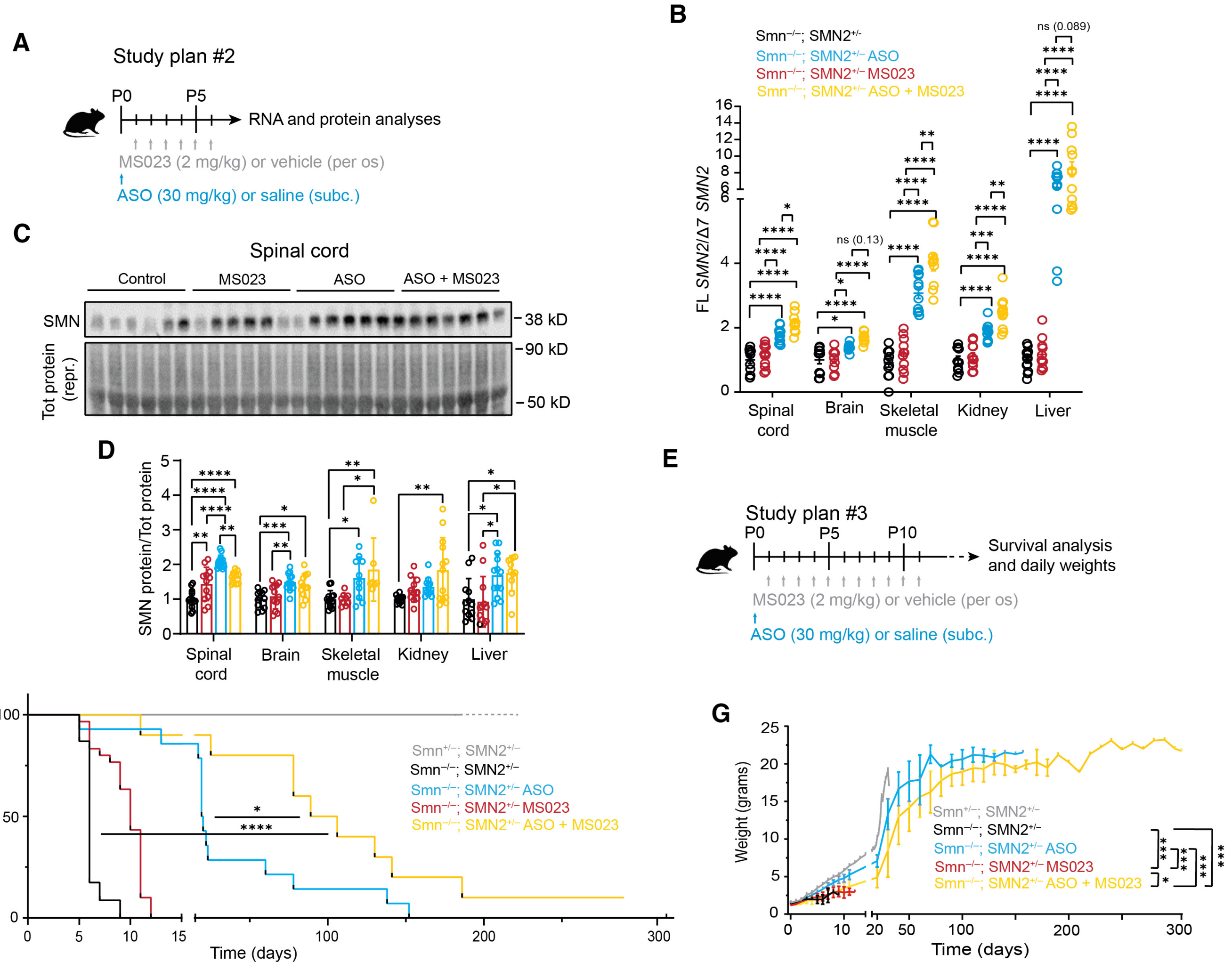

Figure 4. Combinatorial treatment with MS023 and ASO exerts synergistic effects in SMA mice.

A Diagrams of the study design. Study plan #2: At P0 SMA mice were injected subcutaneously with *SMN2*-targeting ASO (30 mg/kg) or saline; from P1 mice were treated daily with oral administrations of MS023 (2 m/kg) or vehicle (0.5% DMSO in saline) using a Hamilton syringe until P6. At P7, mice were sacrificed and tissues collected for analysis.

B FL *SMN2* transcript levels relative to Δ7 *SMN2* in spinal cord, brain, skeletal muscle, kidney and liver of treated SMA mice compared to vehicle-treated SMA mice were normalised to one. Each dot represents a biological replicate (n = 10–12).

C Western blot showing SMN protein levels upon the indicated treatments in spinal cords of SMA mice (top). A representative section of total protein stain, used for protein normalisation, is also shown (bottom). Size in kilodalton is indicated on the right.

D Quantification of SMN protein levels relative to total protein is shown. Each dot represents a biological replicate (n = 7–13).

E Study design #3: At P0 mice were injected subcutaneously with *SMN2*-targeting ASO (30 mg/kg) or saline. From P1 mice were treated daily with oral administrations of MS023 (2 m/kg) or vehicle (0.5% DMSO in saline) using a Hamilton syringe until P12. Mice were weighed daily until they reached their humane end point.

F Kaplan–Meier survival estimation of unaffected mice (Smn$^{+/-}$; SMN2$^{+/-}$) and SMA affected mice (Smn$^{-/-}$; SMN2$^{+/-}$) treated with *SMN2*-targeting ASO (n = 15), MS023 (n = 14), *SMN2*-targeting ASO and MS023 (n = 10), and vehicle (n = 23).

G Body weights of unaffected (n = 12) and SMA mice (vehicle-treated: n = 7; ASO: n = 13; MS023; n = 22; ASO + MS023: n = 10) from postnatal day 0 are shown. Unaffected mice were followed until 25 weeks of age.

Data information: (B), (D), (F), and (G), Data are represented as mean ± s.e.m. and compared with a one-way ANOVA test with multiple comparisons (*$P \le 0.05$; **$P \le 0.01$; ***$P \le 0.001$, ****$P \le 0.0001$) (B, D, G) or with a Mantel-Cox test (*$P \le 0.05$; ****$P \le 0.0001$) (F).
Source data are available online for this figure.

group of over 4,000 proteins, many of which are RNA-binding proteins, influencing their stability, function, and interaction with other macromolecules (Hornbeck *et al*, 2015). In mammals, arginine methylation of histone and non-histone proteins is performed by nine PRMT enzymes, divided into three families: Type I PRMTs (PRMT1, PRMT2, PRMT3, PRMT4, PRMT6, and PRMT8) perform monomethylation followed by an asymmetric dimethylation (ADMA) of arginine, with both methyl groups on a single guanidino nitrogen atom; type II PRMTs (PRMT5 and PRMT9) catalyse monomethylation, followed by a symmetric dimethylation (SDMA) where

© 2023 The Authors

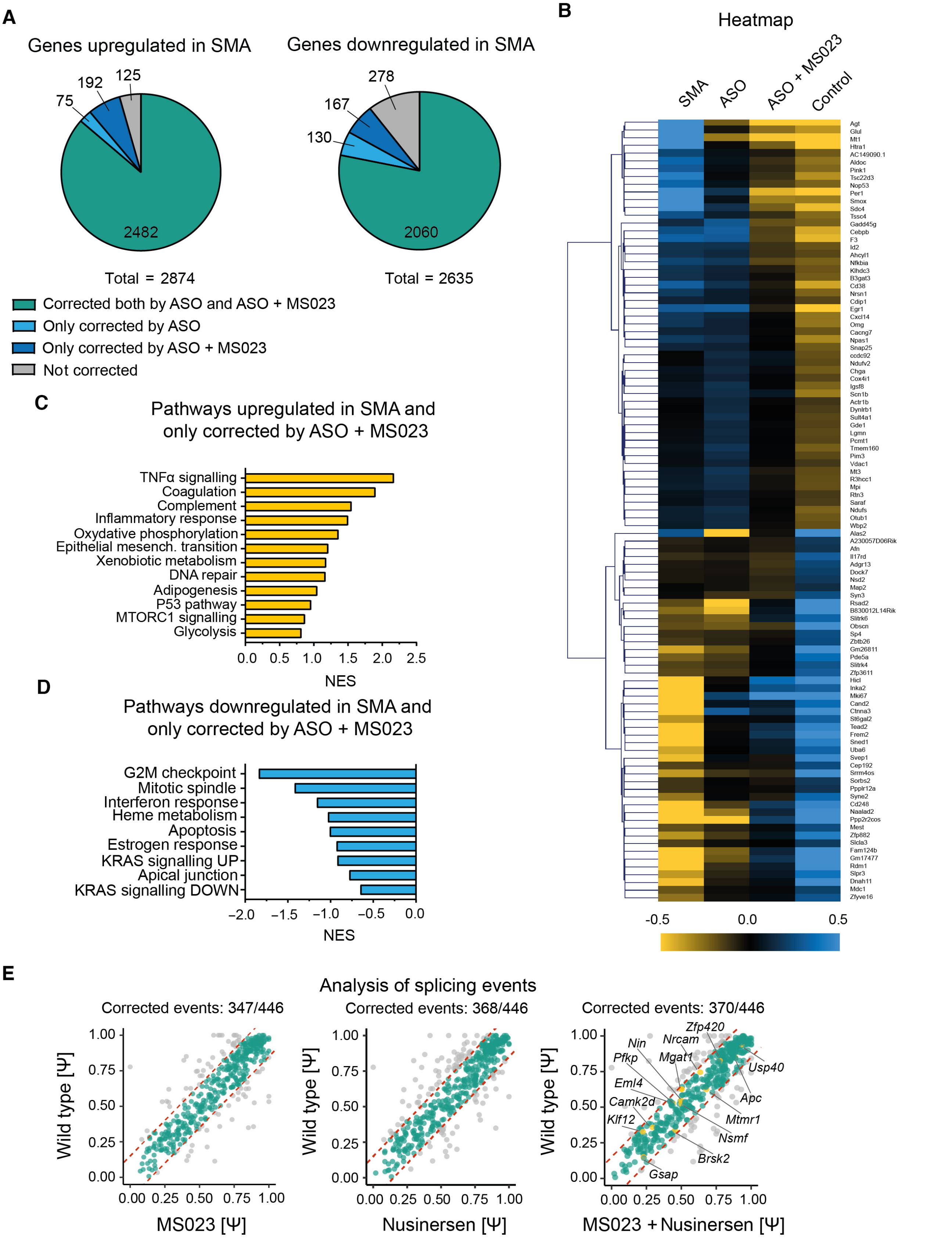
A
Genes upregulated in SMA
75
192
125
2482
Total = 2874
Genes downregulated in SMA
278
167
130
2060
Total = 2635
Corrected both by ASO and ASO + MS023
Only corrected by ASO
Only corrected by ASO + MS023
Not corrected
B
Heatmap
SMA
ASO
ASO + MS023
Control
-0.5
0.0
0.5
C
Pathways upregulated in SMA and only corrected by ASO + MS023
TNFα signalling
Coagulation
Complement
Inflammatory response
Oxydative phosphorylation
Epithelial mesench. transition
Xenobiotic metabolism
DNA repair
Adipogenesis
P53 pathway
MTORC1 signalling
Glycolysis
0.0 0.5 1.0 1.5 2.0 2.5
NES
D
Pathways downregulated in SMA and only corrected by ASO + MS023
G2M checkpoint
Mitotic spindle
Interferon response
Heme metabolism
Apoptosis
Estrogen response
KRAS signalling UP
Apical junction
KRAS signalling DOWN
−2.0 −1.5 −1.0 −0.5 0.0
NES
E
Analysis of splicing events
Corrected events: 347/446
Corrected events: 368/446
Corrected events: 370/446
Wild type [Ψ]
MS023 [Ψ]
Nusinersen [Ψ]
MS023 + Nusinersen [Ψ]
0.00 0.25 0.50 0.75 1.00
Zfp420
Nin
Nrcam
Pfkp
Mgat1
Usp40
Eml4
Camk2d
Apc
Klf12
Mtmr1
Nsmf
Brsk2
Gsap

Figure 5.

Figure 5. Combinatorial treatment with MS023 and ASO results in improved correction of the SMA transcriptomic signature compared to ASO alone.

A Pie charts showing the proportion of transcripts normalised upon the treatments among the genes that are upregulated (left) and downregulated (right) in SMA mice.

B Normalised counts were used to generate the hierarchical clustering heatmap. Upregulated and downregulated genes are displayed in yellow and blue, respectively. Control refers to unaffected ($Smn^{+/-}$; $SMN2^{+/-}$) mice.

C, D (C) Hallmark pathway analysis of genes upregulated and (D) downregulated in SMA corrected by the combinatorial treatment only.

E Plot charts show the distribution of splicing events (Ψ) in SMA mice upon treatment relative to wild-type littermates. The red dotted lines mark the ± 15% normalisation range, corrected values are indicated in green.

one methyl group is transferred to each of the nitrogen atoms; and type III PRMT (PRMT7) only performs monomethylation (MMA) (Guccione & Richard, 2019). PRMTs have been shown to compete over the same substrates, frequently with opposite functional effects (Dhar *et al*, 2013).

Several studies have identified a link between PRMTs and SMN: to ensure fidelity of loading onto the correct snRNAs, three of the core spliceosomal Sm proteins (SmB, SmD1, and SmD3) undergo symmetric dimethylation by PRMT5, a modification recognised by the Tudor domain of SMN (Meister *et al*, 2001; Friesen *et al*, 2001b). SMN interaction with senataxin, a DNA-RNA helicase, depends on the dimethylated arginine and is reduced upon PRMT5 knockdown (Yanling Zhao *et al*, 2016). Lastly, levels of PRMT4 (CARM1) are upregulated in SMA mice spinal cord and patients' cells (Sanchez *et al*, 2015). PRMTs represent a promising therapeutic target for many human diseases, from cancer (Wang *et al*, 2016; Drew *et al*, 2017; Wu *et al*, 2022) to neurodegeneration (Dormann *et al*, 2012; Scaramuzzino *et al*, 2015; Suárez-Calvet *et al*, 2016), with eight PRMT inhibitors attaining clinical trial testing in human cancers (Yang & Bedford, 2013; Blanc & Richard, 2017; Guccione & Richard, 2019; Hwang *et al*, 2021). Consistent with previous methylome analyses in cells treated with type I PRMT inhibitors, GSK3368712 (Noto *et al*, 2020) or MS023 (Karuppagounder *et al*, 2016), we propose that the MS023 mechanism of action entails a switch in the arginine methylation profile from ADMA to SDMA/MMA of HNRNPA1, a major negative regulator of *SMN2* exon 7 inclusion (Cartegni *et al*, 2006; Kashima *et al*, 2007b; Bose *et al*, 2008; Chen *et al*, 2008; Hua *et al*, 2008; Koed Doktor *et al*, 2011; Xiao *et al*, 2012; Singh *et al*, 2013), resulting in reduced binding affinity to *SMN2* pre-mRNA. This mechanism of action is different from the one recently proposed for risdiplam, which entails stabilisation of a yet unidentified RNP complex by binding to distinct sites of *SMN2* pre-mRNA (Sivaramakrishnan *et al*, 2017). Notably, HNRNPA1 has been shown to modulate exon 7 splicing by directly binding *SMN2* pre-mRNA across multiple sites, which include enhancer regions SE1 and SE2, as well as the ISS-N1 region (Lejman *et al*, 2021). Therefore, here we postulate that MS023 enhances the effect of nusinersen by preventing HNRNPA1 multisite binding and concomitantly increasing the overall accessibility of the oligonucleotide to the ISS-N1 region. Which are the treatment-responsive HNRNPA1 methylation sites and how HNRNPA1 binding to *SMN2* pre-mRNA changes upon MS023 remain open questions. They could be addressed with techniques such as heavy methyl SILAC (Ong *et al*, 2004) and label-free surface plasmon resonance technology to assess MS023-induced changes in methylation profiles and target binding affinity to surface-immobilised *SMN2* exon 7, respectively. Out of the 72 targets identified by arginine methyl proteome profiling of human cells (Fong *et al*, 2019), MS023 changed the methylation pattern of other splicing factors implicated in the regulation of *SMN2* splicing (i.e. HNRNPU, HNRNPA2B1, ELAVL1, RBM10, KHDRBS1, SRSF9, TRA2B, and U2AF1; Singh & Singh, 2018), and the individual contribution of such proteins to the overall effect of this small molecule in SMA models remains to be established.

Hallmark analysis of the 359 genes whose expression levels in spinal cords were only corrected by the combinatorial treatment revealed enrichment of immune-related pathways, such as TNF-α signalling, interferon response, and complement activation, overall suggesting that therapeutic targeting of neuroinflammation is key to achieving optimal and long-lasting effects in SMA. Interestingly, astrocyte dysfunction and chronic microglia activation have been observed early in SMA and other neurological conditions (Eikelenboom *et al*, 2002; Sargsyan *et al*, 2005; Heneka *et al*, 2014; Vukojicic *et al*, 2019) and play a determinative role in the disease pathogenesis (Mcgivern *et al*, 2013; Rindt *et al*, 2015; Zhou *et al*, 2016; Martin *et al*, 2017). Transcriptomic data in the preclinical model indicates that MS023 shows a favourable safety profile, with minimal off-target effects both on gene expression and splicing alterations, probably due to the low dose required to achieve *SMN* modulation compared to cancer applications (i.e. 80 mg/kg/day; Fong *et al*, 2019) and in stark contrast to the pleiotropic effects of other epigenetic modulators such as valproic acid.

Altogether, these promising preclinical results warrant further clinical investigations of MS023 or other selective type I PRMTs inhibitors both as a stand-alone and as an add-on treatment with nusinersen in SMA patients.

Materials and Methods

Small molecules screening

The library of small molecules was obtained from the Structural Genomic Consortium (SGC) (Williamson, 2000). Upon reception the molecules were diluted in DMSO to 10 mM, aliquoted and stored at −20°C for the duration of the study. All the compounds, their targets and doses used in the study are listed in Table EV1.

Cell lines and culture

Cells were grown in a humidified incubator at 37°C with 5% CO_2. SMA type I and II patient fibroblasts were obtained from Coriell Institute (GM00232, GM03813). SMA type III patient fibroblasts were a kind gift of the Talbot's lab (University of Oxford). The cells were maintained in Dulbecco's Modified Eagle Medium GlutaMAX (Gibco) supplemented with 10% foetal bovine serum (Gibco) and

© 2023 The Authors

1× Antibiotic-Antimycotic (Gibco). SMA patient fibroblasts were plated in triplicates, in 12-well plates at 50,000 cells per well in 500 μl of medium for RNA or in duplicates in 6-well plates at 100,000 cells per well in 1 ml of medium for protein. After 6 h, compounds of interest were diluted in medium to 2× final concentration and added to the cells. RNA was isolated after 48 h incubation, protein after 72 h.

Cell viability assay

Cells were routinely tested for mycoplasma. The MTS Cell Proliferation Assay Kit (Abcam) was used to determine the highest non-toxic concentration of small molecules in SMA patient fibroblasts. Briefly, 3,000 cells/wells were plated in triplicate in a 96-well plate in half of the final volume of medium (100 μl). After 6 h, compounds of interest were diluted to 2× final concentration in 100 μl of medium and added in a range of concentrations (1–10 μM). 1 μM of staurosporine (Sigma) was used as a positive control for cell death. After 48 h, 20 μl of MTS reagent was added to each well. Four hours later, the absorbance was measured at 490 nm on CLARIOstar plate reader (BMG Labtech). The concentration was deemed non-toxic if the absorbance was not significantly different from the control and the cells looked viable after visual inspection. In subsequent experiments, only the highest non-toxic concentration of each compound was used, unless stated otherwise.

RNA/cDNA preparation and RT-qPCR

RNA extraction was performed using the Maxwell® RSC simply RNA Kit (Promega) following the manufacturer's instructions. The concentration was measured using a Nanodrop 1000 spectrophotometer (Thermo Fisher), and cDNA was generated using an ABI High Capacity cDNA Reverse Transcription Kit (Invitrogen) following the manufacturer's instructions. A qPCR reaction using Power SYBR Green Master Mix (Life Technologies) was performed and analysed on an Applied Biosystems StepOnePlus™ real-time PCR system (Life Technologies). FL *SMN2*, Δ7 *SMN2*, Tot *SMN2*, *PolJ*, *Gapdh*, and *GAPDH* transcripts were amplified using gene- and species-specific primers (Table EV3).

Protein extraction and Western blot

Proteins were harvested from ~ 30 mg of tissue (*in vivo*) or two 6-well plates (*in vitro*) and homogenised in RIPA buffer with complete mini-proteinase inhibitors (Roche). The preparation of nuclear and cytoplasmic extracts from human fibroblasts was performed using NE-PER Nuclear and Cytoplasmic Extraction Reagents (Thermo Fisher), as per manufacturer's instructions. Proteins (10–15 μg from cells and 20–30 μg from tissue) were probed for human SMN protein using anti-SMN, clone SMN-KH monoclonal IgG1 (Sigma, MABE230), Histone 3 (Cell Signalling, 9715), Vinculin (Sigma, 062M4762), HNRNPA1 (Santa Cruz, 4B10), MMA (Cell Signalling, 8015), ADMA (Sigma Aldrich, 07-414), SDMA (Cell Signalling, 13222), β-tubulin (Abcam, 108342), and FAST green total protein stain and secondary antibody IRDye 800CW goat anti-mouse IgG (LI-COR Biosciences). Membranes were imaged on a LI- COR Odyssey FC imager and analysed with Image Studio™ software (LI-COR Biosciences).

Crosslinking and immunoprecipitation assay (CLIP)

Briefly, for each condition, four 150 cm^2 cell dishes of human patient fibroblasts were treated with 100 nM, 250 nM or 1 μM of MS023 for 48 h. After the incubation, the medium was aspirated, and the cells were subjected to 150 mJ/cm^2 254 nm UV light in a Stratalinker UV Crosslinker and pelleted. The cells were then lysed in NP-40 lysis buffer and HNRNPA1 immunoprecipitation was performed with Dynabeads Protein G (Thermo Fisher) and the hnRNAP1 antibody (Santa Cruz). Following DNA and protein digestion, RNA was isolated and cDNA was generated as previously outlined. A qPCR reaction using Power SYBR Green Master Mix (Life Technologies) was performed and analysed on an Applied Biosystems StepOnePlus™ real-time PCR system (Life Technologies). FL *SMN2* and *GAPDH* transcripts were amplified using gene- and species-specific primers.

Protein immunoprecipitation

To detect changes in HNRNPA1 arginine methylation, SMA patient fibroblasts were either left untreated or treated with increasing concentrations of MS023 (100 nM, 250 nM or 1 μM) for 48 h. Cell pellets were lysed in NP-40 buffer, and HNRNPA1 immunoprecipitation was performed with Dynabeads Protein G (Thermo Fisher, 10003D) and hnRNAP1 antibody (Santa Cruz), following the manufacturer's protocol. Subsequently, Western blots were run according to the protocol described above.

Mice

Mice were housed and all the procedures were carried out at the Biomedical Services Building, University of Oxford, and authorised by the UK Home Office in accordance with the Animals (Scientific Procedures) Act 1986 and by the University of Oxford ethics committee (PPL no: PDFEDC6F0). Mice were housed in biosecurity level 2, with a 12 h night/day cycle, in transparent plastic cages with daily water and food exchange. The pups were housed with their mother only (no father) until day 21, when they were weaned off into separate cages for males and females, with a maximum of six adult mice in one cage. Both male and female staff attended to the mice and performed experiments. ARRIVE guidelines were followed. All experiments were performed on the SMA mouse strain FVB.Cg-Smn1^{tm1Hung}Tg(SMN2)2Hung/J—the "Taiwanese" model (*Smn*$^{-/-}$; *SMN2*$^{+/-}$), generated and maintained as previously described (Hsieh-Li *et al*, 2000; Gogliotti *et al*, 2010). MS023 was administered daily orally from P0 or P1 using a Hamilton syringe (Hamilton). Doses of 0, 1, 2, 5, or 40 mg/kg of MS023 were diluted in 0.5% DMSO and 0.9% saline and administered at a volume of 5 μl/g of body weight. Nusinersen (sequence: U*sC*sA*sC*sU*sU*sU*sC*sA*sU*sA*sA*sU*sG*sC*sU*sG*sG*s, where "S" is phosphorothioate backbone and "*" is a 2′-O-(2-Methoxyethyl)-oligoribonucleotides chemistry) was diluted in 0.9% saline and given once at 20 μl/g body weight, via subcutaneous injection at P0, in a dose of 30 mg/kg. Weights were recorded, and overall health was assessed daily. Since the pups were treated daily from P0 and identifying and marking individual pups between P0 and P7 poses a high risk of misidentifying an animal, in this study a litter constitutes an experimental unit, and all mice in the litter were subjected to the

same treatment. Only litters between 7 and 11 pups were used to correct for average weight (mice in smaller litters tend to be bigger and live longer, and the opposite is true for litters of 12 and above), and treatment was allocated randomly to a litter before it was born. All the experimental units treated were included in the analysis. Personnel performing daily weights and welfare checks for combinatorial therapy were blinded; researcher performing oral administration, injections, and data analysis was not blinded. For survival analysis, the humane end point were reached upon 15% weight loss from a maximum weight or when the mouse was not able to right itself for 30 s. Mice were culled by decapitation (if younger than postnatal day 10) or cervical dislocation (if 10 days old or older). Tissues were harvested on the indicated postnatal day.

RNA sequencing

Transcriptomic analysis was performed by Novogene (UK) Company Limited (https://en.novogene.com/) on P7 spinal cords of mice in the following treatment groups: untreated SMA mice, SMA mice treated with nusinersen, SMA mice treated with MS023, SMA mice treated with nusinersen and MS023, and untreated controls (four biological replicates in each group). RNA quantification and integrity were assessed using the RNA Nano 6000 Assay Kit of the Bioanalyzer 2100 system (Agilent Technologies, CA, USA). mRNA was purified using poly-T oligo-attached magnetic beads. Fragmentation was carried out using divalent cations under elevated temperatures in First Strand Synthesis Reaction Buffer. The first strand cDNA was synthesised using random hexamer primers and M-MuLV Reverse Transcriptase. Second-strand cDNA synthesis was subsequently performed using DNA Polymerase I and RNase H. The remaining overhangs were converted into blunt ends via exonuclease/polymerase activities. After adenylation of 3′ ends of DNA fragments, adaptor with hairpin loop structure were ligated to prepare for hybridisation. In order to select cDNA fragments of preferentially 370–420 bp in length, the library fragments were purified with AMPure XP system (Beckman Coulter, Beverly, USA). PCR products were purified (AMPure XP system) and library quality was assessed on the Agilent Bioanalyzer 2100 system. The clustering of the index-coded samples was performed on a cBot Cluster Generation System using TruSeq PE Cluster Kit v3-cBot-HS (Illumina) according to the manufacturer's instructions. After cluster generation, the library preparations were sequenced on an Illumina Novaseq platform with a coverage of 25 million reads and 150 bp paired-end reads were generated. Raw data (raw reads) of fastq format were firstly processed through in-house perl scripts. In this step, clean data (clean reads) were obtained by removing reads containing adapter, reads 1 containing ploy-N and low-quality reads from raw data. Mus Musculus (GRCm38/mm10) reference genome was used, index of the reference genome was built using Hisat2 v2.0.5 and paired-end clean reads were aligned to the reference genome using Hisat2 v2.0.5. The mapped reads of each sample were assembled by StringTie (v1.3.3b; Pertea *et al*, 2015) in a reference-based approach. Quantification of gene expression level Feature Counts v1.5.0-p3 was used to count the reads numbers mapped to each gene. Differential expression analysis was performed using the DESeq2 R package (1.20.0), Benjamini-Hochberg-adjusted *P*-values reported. A corrected *P*-value of 0.05 and absolute fold change of 2 were set as the threshold for significantly differential expression. Alternative splicing analysis rMATS (4.1.0) software was used to analysis the splicing event. We used the *psiPerEvent* operation of SUPPA to calculate the Ψ values from the transcript quantifications obtained for all the alternative splicing events generated as described above with the *generateEvents* module of SUPPA (Alamancos*et al*, 2014). The data were visualised with R (www.r-project.org/).

> **The paper explained**
>
> **Problem**
> Spinal muscular atrophy (SMA) is a neuromuscular disorder resulting from inactivating mutations in the survival motor neuron 1 (*SMN1*) gene, making it a prominent genetic contributor to infant mortality on a global scale. Over the past 10 years, the emergence of effective treatments has notably decelerated the progression of the disease and enhanced well-being of individuals affected by SMA. However, these treatments have several disadvantages, including substantial costs and side effects associated with long-term use. Consequently, there is a pressing need for novel therapeutic approaches, whether used independently or in conjunction with existing ones.
>
> **Results**
> From a screen of potent and highly selective small molecules, a protein methyltransferases (PRMTs) inhibitor, MS023, was identified, capable of elevating SMN protein levels in SMA models and to synergistically amplify the effects of nusinersen, a clinically approved antisense oligonucleotide (ASO) for SMA patients.
>
> **Impact**
> These results highlight a link between protein arginine methylation and SMN regulation. Furthermore, these data provide proof of concept evidence for the use of MS023, a potent and selective PRMTs inhibitor, both as a stand-alone and an add-on treatment for SMA patients.

Study approval

All animal procedures were authorised by the UK Home Office in accordance with the Animals (Scientific Procedures) Act 1986 and by the University of Oxford ethics committee (PPL no: PDFEDC6F0).

Statistics

ANOVA tests were used to compare the means between two or more groups, respectively. Statistics of survival times of SMA mice were determined by Kaplan-Meier estimation, and comparisons were made with the log-rank test. A two-way ANOVA was conducted to compare the effect of the treatment on the weights of the animals using treatment as a between-subjects factor and time as a within-subjects factor. Power analysis was performed using G*Power 3.1.9.2 software (Erdfelder *et al*, 2009). GraphPad Prism version 8 was used to perform the statistical analyses (GraphPad, La Jolla, CA). A *P*-value < 0.05 was set as statistically significant.

Data availability

The RNA-seq datasets produced in this study (Fig 5) are available in the following database: Gene Expression Omnibus (GEO), accession

© 2023 The Authors

number GSE206400 (https://www.ncbi.nlm.nih.gov/geo/query/acc.cgi?acc=GSE206400).

Expanded View for this article is available online.

Acknowledgements

This study was supported by a Career Development Fellowship grant from the Wellcome Trust to CR (205162/Z/16/Z). AJK was supported by a Medical Research Council programme grant (BRT00040). WFL was supported by a Medical Research Council programme grant to MJAW (MR/N024850/1). The authors would like to thank the Structural Genomics Consortium for providing the library of small molecules and the Animal Facility staff for their assistance with the animals.

Author contributions

Anna J Kordala: Conceptualization; data curation; formal analysis; methodology; writing – original draft; writing – review and editing. **Jessica Stoodley:** Conceptualization; data curation; formal analysis; writing – review and editing. **Nina Ahlskog:** Data curation; formal analysis; writing – review and editing. **Muhammad Hanifi:** Data curation; formal analysis; writing – review and editing. **Antonio Garcia Guerra:** Data curation; formal analysis. **Amarjit Bhomra:** Data curation; formal analysis; writing – review and editing. **Wooi Fang Lim:** Data curation; writing – review and editing. **Lyndsay M Murray:** Writing – review and editing. **Kevin Talbot:** Resources; writing – review and editing. **Suzan M Hammond:** Conceptualization; writing – review and editing. **Matthew JA Wood:** Conceptualization; resources; funding acquisition; writing – review and editing. **Carlo Rinaldi:** Conceptualization; resources; data curation; formal analysis; funding acquisition; methodology; writing – original draft; project administration; writing – review and editing.

Disclosure and competing interests statement

The authors declare that they have no conflict of interest.

References

Ackloo S, Brown PJ, Müller S (2017) Chemical probes targeting epigenetic proteins: applications beyond oncology. *Epigenetics* 12: 378–400

Akten B, Kye MJ, Hao LT, Wertz MH, Singh S, Nie D, Huang J, Merianda TT, Twiss JL, Beattie CE *et al* (2011) Interaction of survival of motor neuron (SMN) and HuD proteins with mRNA cpg15 rescues motor neuron axonal deficits. *Proc Natl Acad Sci USA* 108: 10337–10342

Alamancos GP, Agirre E, Eyras E (2014) Methods to study splicing from high-throughput RNA sequencing data. *Methods Mol Biol* 1126: 357–397

Allis CD, Berger SL, Cote J, Dent S, Jenuwien T, Kouzarides T, Pillus L, Reinberg D, Shi Y, Shiekhattar R *et al* (2007) New nomenclature for chromatin-modifying enzymes. *Cell* 131: 633–636

Araujo A, Araujo M, Swoboda KJ (2009) Vascular perfusion abnormalities in infants with spinal muscular atrophy. *J Pediatr* 155: 292–294

Arnold AS, Gueye M, Guettier-Sigrist S, Courdier-Fruh I, Coupin G, Poindron P, Gies JP (2004) Reduced expression of nicotinic AChRs in myotubes from spinal muscular atrophy I patients. *Lab Invest* 84: 1271–1278

Arrowsmith CH, Bountra C, Fish PV, Lee K, Schapira M (2012) Epigenetic protein families: a new frontier for drug discovery. *Nat Rev Drug Discov* 11: 384–400

Bäumer D, Lee S, Nicholson G, Davies JL, Parkinson NJ, Murray LM, Gillingwater TH, Ansorge O, Davies KE, Talbot K (2009) Alternative splicing events are a late feature of pathology in a mouse model of spinal muscular atrophy. *PLoS Genet* 5: e1000773

Bevan AK, Hutchinson KR, Foust KD, Braun L, McGovern VL, Schmelzer L, Ward JG, Petruska JC, Lucchesi PA, Burghes AHM *et al* (2010) Early heart failure in the SMNDelta7 model of spinal muscular atrophy and correction by postnatal scAAV9-SMN delivery. *Hum Mol Genet* 19: 3895–3905

Blanc RS, Richard S (2017) Arginine methylation: the coming of age. *Mol Cell* 65: 8–24

Bose JK, Wang IF, Hung L, Tarn WY, Shen CKJ (2008) TDP-43 overexpression enhances exon 7 inclusion during the survival of motor neuron pre-mRNA splicing. *J Biol Chem* 283: 28852–28859

Bowerman M, Swoboda KJ, Michalski JP, Wang GS, Reeks C, Beauvais A, Murphy K, Woulfe J, Screaton RA, Scott FW *et al* (2012) Glucose metabolism and pancreatic defects in spinal muscular atrophy. *Ann Neurol* 72: 256–268

Cao YY, Qu YJ, He SX, Li Y, Bai JL, Jin YW, Wang H, Song F (2016) Association between SMN2 methylation and disease severity in Chinese children with spinal muscular atrophy. *J Zhejiang Univ Sci B* 17: 76–82

Cartegni L, Hastings ML, Calarco JA, De Stanchina E, Krainer AR (2006) Determinants of exon 7 splicing in the spinal muscular atrophy genes, SMN1 and SMN2. *Am J Hum Genet* 78: 63–77

Charroux B, Pellizzoni L, Perkinson RA, Shevchenko A, Mann M, Dreyfuss G (1999) Gemin3: a novel DEAD box protein that interacts with SMN, the spinal muscular atrophy gene product, and is a component of gems. *J Cell Biol* 147: 1181–1193

Chen H-H, Chang J-G, Lu R-M, Peng T-Y, Tarn W-Y (2008) The RNA binding protein hnRNP Q modulates the utilization of exon 7 in the survival motor neuron 2 (SMN2) gene. *Mol Cell Biol* 28: 6929–6938

Chiriboga CA, Swoboda KJ, Darras BT, Iannaccone ST, Montes J, De Vivo DC, Norris DA, Bennett CF, Bishop KM (2016) Results from a phase 1 study of nusinersen (ISIS-SMN(Rx)) in children with spinal muscular atrophy. *Neurology* 86: 890–897

Crawford TO, Pardo CA (1996) The neurobiology of childhood spinal muscular atrophy. *Neurobiol Dis* 3: 97–110

Crawford TO, Sladky JT, Hurko O, Besner-Johnston A, Kelley RI (1999) Abnormal fatty acid metabolism in childhood spinal muscular atrophy. *Ann Neurol* 45: 337–343

Custer SK, Gilson TD, Li H, Todd AG, Astroski JW, Lin H, Liu Y, Androphy EJ (2016) Altered mRNA splicing in SMN-depleted motor neuron-like cells. *PloS One* 11: e0163954

Deguise MO, Baranello G, Mastella C, Beauvais A, Michaud J, Leone A, De Amicis R, Battezzati A, Dunham C, Selby K *et al* (2019) Abnormal fatty acid metabolism is a core component of spinal muscular atrophy. *Ann Clin Transl Neurol* 6: 1519–1532

Dhar S, Vemulapalli V, Patananan AN, Huang GL, Di Lorenzo A, Richard S, Comb MJ, Guo A, Clarke SG, Bedford MT (2013) Loss of the major Type I arginine methyltransferase PRMT1 causes substrate scavenging by other PRMTs. *Sci Rep* 3: 1311

Doktor TK, Hua Y, Andersen HS, Brøner S, Liu YH, Wieckowska A, Dembic M, Bruun GH, Krainer AR, Andresen BS (2017) RNA-sequencing of a mouse-model of spinal muscular atrophy reveals tissue-wide changes in splicing of U12-dependent introns. *Nucleic Acids Res* 45: 395–416

Dormann D, Madl T, Valori CF, Bentmann E, Tahirovic S, Abou-Ajram C, Kremmer E, Ansorge O, MacKenzie IRA, Neumann M *et al* (2012) Arginine methylation next to the PY-NLS modulates Transportin binding and nuclear import of FUS. *EMBO J* 31: 4258–4275

Drew AE, Moradei O, Jacques SL, Rioux N, Boriack-Sjodin AP, Allain C, Scott MP, Jin L, Raimondi A, Handler JL *et al* (2017) Identification of a CARM1

inhibitor with potent *in vitro* and *in vivo* activity in preclinical models of multiple myeloma. *Sci Rep* 7: 17993

Eikelenboom P, Bate C, Van Gool WA, Hoozemans JJM, Rozemuller JM, Veerhuis R, Williams A (2002) Neuroinflammation in Alzheimer's disease and prion disease. *Glia* 40: 232 – 239

Eram MS, Shen Y, Szewczyk MM, Wu H, Senisterra G, Li F, Butler KV, Kaniskan HÜ, Speed BA, Dela Seña C *et al* (2016) A potent, selective, and cell-active inhibitor of human type I protein arginine methyltransferases. *ACS Chem Biol* 11: 772 – 781

Erdfelder E, FAul F, Buchner A, Lang AG (2009) Statistical power analyses using G*Power 3.1: tests for correlation and regression analyses. *Behav Res Methods* 41: 1149 – 1160

Fallini C, Zhang H, Su Y, Silani V, Singer RH, Rossoll W, Bassell GJ (2011) The survival of motor neuron (SMN) protein interacts with the mRNA-binding protein HuD and regulates localization of poly(A) mRNA in primary motor neuron axons. *J Neurosci* 31: 3914 – 3925

Fallini C, Rouanet JP, Donlin-Asp PG, Guo P, Zhang H, Singer RH, Rossoll W, Bassell GJ (2014) Dynamics of survival of motor neuron (SMN) protein interaction with the mRNA-binding protein IMP1 facilitates its trafficking into motor neuron axons. *Dev Neurobiol* 74: 319 – 332

Fallini C, Donlin-Asp PG, Rouanet JP, Bassell GJ, Rossoll W (2016) Deficiency of the survival of motor neuron protein impairs mRNA localization and local translation in the growth cone of motor neurons. *J Neurosci* 36: 3811 – 3820

Feldkötter M, Schwarzer V, Wirth R, Wienker TF, Wirth B (2002) Quantitative analyses of SMN1 and SMN2 based on real-time lightCycler PCR: fast and highly reliable carrier testing and prediction of severity of spinal muscular atrophy. *Am J Hum Genet* 70: 358 – 368

Finkel RS, Chiriboga CA, Vajsar J, Day JW, Montes J, De Vivo DC, Yamashita M, Rigo F, Hung G, Schneider E *et al* (2016) Treatment of infantile-onset spinal muscular atrophy with nusinersen: a phase 2, open-label, dose-escalation study. *Lancet* 388: 3017 – 3026

Finkel RS, Mercuri E, Darras BT, Connolly AM, Kuntz NL, Kirschner J, Chiriboga CA, Saito K, Servais L, Tizzano E *et al* (2017) Nusinersen versus sham control in infantile-onset spinal muscular atrophy. *N Engl J Med* 377: 1723 – 1732

Fong JY, Pignata L, Goy PA, Kawabata KC, Lee SCW, Koh CM, Musiani D, Massignani E, Kotini AG, Penson A *et al* (2019) Therapeutic targeting of RNA splicing catalysis through inhibition of protein arginine methylation. *Cancer Cell* 36: 194 – 209.e9

Friesen WJ, Massenet S, Paushkin S, Wyce A, Dreyfuss G (2001a) SMN, the product of the spinal muscular atrophy gene, binds preferentially to dimethylarginine-containing protein targets sufficient for SMN binding. *Mol Cell* 7: 1111 – 1117

Friesen WJ, Paushkin S, Wyce A, Massenet S, Pesiridis GS, Van Duyne G, Rappsilber J, Mann M, Dreyfuss G (2001b) The methylosome, a 20S complex containing JBP1 and pICln, produces dimethylarginine-modified Sm proteins. *Mol Cell Biol* 21: 8289 – 8300

Gogliotti RG, Hammond SM, Lutz C, Didonato CJ (2010) Molecular and phenotypic reassessment of an infrequently used mouse model for spinal muscular atrophy. *Biochem Biophys Res Commun* 391: 517 – 522

Gogliotti RG, Quinlan KA, Barlow CB, Heier CR, Heckman CJ, DiDonato CJ (2012) Motor neuron rescue in spinal muscular atrophy mice demonstrates that sensory-motor defects are a consequence, not a cause, of motor neuron dysfunction. *J Neurosci* 32: 3818 – 3829

Guccione E, Richard S (2019) The regulation, functions and clinical relevance of arginine methylation. *Nat Rev Mol Cell Biol* 20: 642 – 657

Haché M, Swoboda KJ, Sethna N, Farrow-Gillespie A, Khandji A, Xia S, Bishop KM (2016) Intrathecal injections in children with spinal muscular atrophy: nusinersen clinical trial experience. *J Child Neurol* 31: 899 – 906

Hammond SM, Hazell G, Shabanpoor F, Saleh AF, Bowerman M, Sleigh JN (2016) Systemic peptide-mediated oligonucleotide therapy improves long-term survival in spinal muscular atrophy. *Proc Natl Acad Sci USA* 113: 10962 – 10967

Harding BN, Kariya S, Monani UR, Chung WK, Benton M, Yum SW, Tennekoon G, Finkel RS (2015) Spectrum of neuropathophysiology in spinal muscular atrophy type I. *J Neuropathol Exp Neurol* 74: 15 – 24

Hauke J, Riessland M, Lunke S, Eyüpoglu IY, Blümcke I, El-osta A, Wirth B, Hahnen E (2009) Survival motor neuron gene 2 silencing by DNA methylation correlates with spinal muscular atrophy disease severity and can be bypassed by histone deacetylase inhibition. *Hum Mol Genet* 18: 304 – 317

Heier CR, Satta R, Lutz C, Didonato CJ (2010) Arrhythmia and cardiac defects are a feature of spinal muscular atrophy model mice. *Hum Mol Genet* 19: 3906 – 3918

Heneka MT, Kummer MP, Latz E (2014) Innate immune activation in neurodegenerative disease. *Nat Rev Immunol* 14: 463 – 477

Hornbeck PV, Zhang B, Murray B, Kornhauser JM, Latham V, Skrzypek E (2015) PhosphoSitePlus, 2014: mutations, PTMs and recalibrations. *Nucleic Acids Res* 43: D512 – D520

Hsieh-Li HM, Chang JG, Jong YJ, Wu MH, Wang NM, Tsai CH, Li H (2000) A mouse model for spinal muscular atrophy. *Nat Genet* 24: 66 – 70

Hua Y, Vickers TA, Okunola HL, Bennett CF, Krainer AR (2008) Antisense masking of an hnRNP A1/A2 intronic splicing silencer corrects SMN2 splicing in transgenic mice. *Am J Hum Genet* 82: 834 – 848

Hubers L, Valderrama-Carvajal H, Laframboise J, Timbers J, Sanchez G, Côté J (2011) HuD interacts with survival motor neuron protein and can rescue spinal muscular atrophy-like neuronal defects. *Hum Mol Genet* 20: 553 – 579

Huo Q, Kayikci M, Odermatt P, Meyer K, Michels O, Saxena S (2014) Splicing changes in SMA mouse motoneurons and SMN-depleted neuroblastoma cells: evidence for involvement of splicing regulatory proteins. *RNA Biol* 11: 1430 – 1446

Hwang JW, Cho Y, Bae GU, Kim SN, Kim YK (2021) Protein arginine methyltransferases: promising targets for cancer therapy. *Exp Mol Med* 53: 788 – 808

Jablonka S, Karle K, Sandner B, Andreassi C, von Au K, Sendtner M (2006) Distinct and overlapping alterations in motor and sensory neurons in a mouse model of spinal muscular atrophy. *Hum Mol Genet* 15: 511 – 518

Karuppagounder SS, Alim I, Khim SJ, Bourassa MW, Sleiman SF, John R, Thinnes CC, Yeh TL, Demetriades M, Neitemeier S *et al* (2016) Therapeutic targeting of oxygen-sensing prolyl hydroxylases abrogates ATF4-dependent neuronal death and improves outcomes after brain hemorrhage in several rodent models. *Sci Transl Med* 8: 328ra29

Kashima T, Manley JL (2003) A negative element in SMN2 exon 7 inhibits splicing in spinal muscular atrophy. *Nat Genet* 34: 460 – 463

Kashima T, Rao N, David CJ, Manley JI (2007a) hnRNP A1 functions with specificity in repression of SMN2 exon 7 splicing. *Hum Mol Genet* 16: 3149 – 3159

Kashima T, Rao N, Manley JL (2007b) An intronic element contributes to splicing repression in spinal muscular atrophy. *Proc Natl Acad Sci USA* 104: 3426 – 3431

Kim JK, Jha NN, Feng Z, Faleiro MR, Chiriboga CA, Wei-Lapierre L, Dirksen RT, Ko CP, Monani UR (2020) Muscle-specific SMN reduction reveals motor neuron-independent disease in spinal muscular atrophy models. *J Clin Invest* 130: 1271 – 1287

Kissel JT, Elsheikh B, King WM, Freimer M, Scott CB, Kolb SJ, Reyna SP, Crawford TO, Simard LR, Krosschell KJ *et al* (2014) SMA valiant trial: a

 © 2023 The Authors

prospective, double-blind, placebo-controlled trial of valproic acid in ambulatory adults with spinal muscular atrophy. *Muscle Nerve* 49: 187–192

Koed Doktor T, Schroeder LD, Vested A, Palmfeldt J, Andersen HS, Gregersen N, Andresen BS (2011) SMN2 exon 7 splicing is inhibited by binding of hnRNP A1 to a common ESS motif that spans the 3′ splice site. *Hum Mutat* 32: 220–230

Krosschell KJ, Kissel JT, Townsend EL, Simeone SD, Zhang RZ, Reyna SP, Crawford TO, Schroth MK, Acsadi G, Kishnani PS *et al* (2018) Clinical trial of L-Carnitine and valproic acid in spinal muscular atrophy type I. *Muscle Nerve* 57: 193–199

Lefebvre S, Burglen L, Reboullet S, Clermont O, Burlet P, Viollet L, Benichou B, Cruaud C, Millasseau P, Zeviani M *et al* (1995) Identification and characterization of a spinal muscular atrophy-determining gene. *Cell* 80: 155–165

Lejman J, Zieliński G, Gawda P, Lejman M (2021) Alternative splicing role in new therapies of spinal muscular atrophy. *Genes (Basel)* 12: 1346

Lipnick SL, Agniel DM, Aggarwal R, Makhortova NR, Finlayson SG, Brocato A, Palmer N, Darras BT, Kohane I, Rubin LL (2019) Systemic nature of spinal muscular atrophy revealed by studying insurance claims. *PLoS One* 14: e0213680

Lorson C, Ahnen E, Androphy EJ, Wirth B (1999) A single nucleotide in the SMN gene regulates splicing and is responsible for spinal muscular atrophy. *Proc Natl Acad Sci USA* 96: 6307–6311

Makarov EM, Owen N, Bottrill A, Makarova OV (2012) Functional mammalian spliceosomal complex E contains SMN complex proteins in addition to U1 and U2 snRNPs. *Nucleic Acids Res* 40: 2639–2652

Marasco LE, Dujardin G, Sousa-Luís R, Liu YH, Stigliano JN, Nomakuchi T, Proudfoot NJ, Krainer AR, Kornblihtt AR (2022) Counteracting chromatin effects of a splicing-correcting antisense oligonucleotide improves its therapeutic efficacy in spinal muscular atrophy. *Cell* 185: 2057–2070.e15

Martin JE, Nguyen TKT, Grunseich C, Nofziger JH, Lee PR, Fields D, Fischbeck KH, Foran E (2017) Decreased motor neuron support by SMA astrocytes due to diminished MCP1 secretion. *J Neurosci* 37: 5309–5318

Martinez TL, Kong L, Wang X, Osborne MA, Crowder ME, Van MJP, Xu X, Davis C, Wooley J, Goldhamer DJ *et al* (2012) Survival motor neuron protein in motor neurons determines synaptic integrity in spinal muscular atrophy. *J Neurosci* 32: 8703–8715

Martínez-Hernández R, Soler-Botija C, Also E, Alias L, Caselles L, Gich I, Bernal S, Tizzano EF (2009) The developmental pattern of myotubes in spinal muscular atrophy indicates prenatal delay of muscle maturation. *J Neuropathol Exp Neurol* 68: 474–481

McAndrew PE, Parsons DW, Simard LR, Rochette C, Ray PN, Mendell JR, Prior TW, Burghes AHM (1997) Identification of proximal spinal muscular atrophy carriers and patients by analysis of SMNT and SMNC gene copy number. *Am J Hum Genet* 60: 1411–1422

Mcgivern JV, Patitucci TN, Nord JA, Barabas MEA, Stucky CL, Ebert AD (2013) Spinal muscular atrophy astrocytes exhibit abnormal calcium regulation and reduced growth factor production. *Glia* 61: 1418–1428

Meister G, Eggert C, Bühler D, Brahms H, Kambach C, Fischer U (2001) Methylation of Sm proteins by a complex containing PRMT5 and the putative U snRNP assembly factor pICln. *Curr Biol* 11: 1990–1994

Mentis GZ, Blivis D, Liu W, Drobac E, Crowder ME, Kong L, Alvarez FJ, Sumner CJ, O'Donovan MJ (2011) Early functional impairment of sensory-motor connectivity in a mouse model of spinal muscular atrophy. *Neuron* 69: 453–467

Mercuri E, Bertini E, Messina S, Solari A, D'Amico A, Angelozzi C, Battini R, Berardinelli A, Boffi P, Bruno C *et al* (2007) Randomized, double-blind, placebo-controlled trial of phenylbutyrate in spinal muscular atrophy. *Neurology* 68: 51–55

Mercuri E, Darras BT, Chiriboga CA, Day JW, Campbell C, Connolly AM, Iannaccone ST, Kirschner J, Kuntz NL, Saito K *et al* (2018) Nusinersen versus Sham control in later-onset spinal muscular atrophy. *N Engl J Med* 378: 625–635

Mercuri E, Pera MC, Scoto M, Finkel R, Muntoni F (2020) Spinal muscular atrophy — insights and challenges in the treatment era. *Nat Rev Neurol* 16: 706–715

Mercuri E, Sumner CJ, Muntoni F, Darras BT, Finkel RS (2022) Spinal muscular atrophy. *Nat Rev Dis Primers* 8: 1–16

Mohseni J, Zabidi-Hussin ZAMH, Sasongko TH (2013) Histone deacetylase inhibitors as potential treatment for spinal muscular atrophy. *Genet Mol Biol* 36: 299–307

Monani UR, Lorson CL, Parsons DW, Prior TW, Androphy EJ, Burghes AHM, McPherson JD (1999) A single nucleotide difference that alters splicing patterns distinguishes the SMA gene SMN1 from the copy gene SMN2. *Hum Mol Genet* 8: 1177–1183

Munsat TL, Davies KE (1992) International SMA Consortium Meeting (26–28 June 1992, Bonn, Germany). *Neuromuscul Disord* 2: 423–428

Murray LM, Beauvais A, Gibeault S, Courtney NL, Kothary R (2015) Transcriptional profiling of differentially vulnerable motor neurons at pre-symptomatic stage in the Smn 2b/—mouse model of spinal muscular atrophy. *Acta Neuropathol Commun* 3: 55

Naryshkin NA, Weetall M, Dakka A, Narasimhan J, Zhao X, Feng Z, Ling KKY, Karp GM, Qi H, Woll MG *et al* (2014) SMN2 splicing modifiers improve motor function and longevity in mice with spinal muscular atrophy. *Science* 345: 688–693

Noto PB, Sikorski TW, Zappacosta F, Wagner CD, Montes de Oca R, Szapacs ME, Annan RS, Liu Y, McHugh CF, Mohammad HP *et al* (2020) Identification of hnRNP-A1 as a pharmacodynamic biomarker of type I PRMT inhibition in blood and tumor tissues. *Sci Rep* 10: 1–19

Ong SE, Mittler G, Mann M (2004) Identifying and quantifying *in vivo* methylation sites by heavy methyl SILAC. *Nat Methods* 1: 119–126

Pearn J (1978) Incidence, prevalence and gene frequency studies of chronic childhood spinal muscular atrophy. *J Med Genet* 15: 409–413

Pellizzoni L, Kataoka N, Charroux B, Dreyfuss G (1998) A novel function for SMN, the spinal muscular atrophy disease gene product, in pre-mRNA splicing. *Cell* 95: 615–624

Pellizzoni L, Baccon J, Charroux B, Dreyfuss G (2001) The survival of motor neurons (SMN) protein interacts with the snoRNP proteins fibrillarin and GAR1. *Curr Biol* 11: 1079–1088

Pellizzoni L, Yong J, Dreyfuss G (2002) Essential role for the SMN complex in the specificity of snRNP assembly. *Science* 298: 1775–1779

Pertea M, Pertea GM, Antonescu CM, Chang TC, Mendell JT, Salzberg SL (2015) StringTie enables improved reconstruction of a transcriptome from RNA-seq reads. *Nat Biotechnol* 33: 290–295

Piazzon N, Rage F, Schlotter F, Moine H, Branlant C, Massenet S (2008) *In vitro* and in cellulo evidences for association of the survival of motor neuron complex with the Fragile X mental retardation protein. *J Biol Chem* 283: 5598–5610

Portela A, Esteller M (2010) Epigenetic modifications and human disease. *Nat Biotechnol* 28: 1057–1068

Rage F, Boulisfane N, Rihan K, Neel H, Gostan T, Bertrand E, Bordonné R, Soret A (2013) Genome-wide identification of mRNAs associated with the protein SMN whose depletion decreases their axonal localization. *RNA* 19: 1755–1766

Ramos DM, D'Ydewalle C, Gabbeta V, Dakka A, Klein SK, Norris DA, Matson J, Taylor SJ, Zaworski PG, Prior TW *et al* (2019) Age-dependent SMN

© 2023 The Authors

expression in disease-relevant tissue and implications for SMA treatment. *J Clin Invest* 129: 4817–4831

Rindt H, Feng Z, Mazzasette C, Glascock JJ, Valdivia D, Pyles N, Crawford TO, Swoboda KJ, Patitucci TN, Ebert AD *et al* (2015) Astrocytes influence the severity of spinal muscular atrophy. *Hum Mol Genet* 24: 4094–4102

Rossoll W, Jablonka S, Andreassi C, Kröning AK, Karle K, Monani UR, Sendtner M (2003) Smn, the spinal muscular atrophy-determining gene product, modulates axon growth and localization of β-Actin mRNA in growth cones of motoneurons. *J Cell Biol* 163: 801–812

Rudnik-Schöneborn S, Goebel HH, Schlote W, Molaian S, Omran H, Ketelsen U, Korinthenberg R, Wenzel D, Lauffer H, Kreiß-Nachtsheim M *et al* (2003) Classical infantile spinal muscular atrophy with SMN deficiency causes sensory neuronopathy. *Neurology* 60: 983–987

Rudnik-Schöneborn S, Vogelgesang S, Armbrust S, Graul-Neumann L, Fusch C, Zerres K (2010) Digital necroses and vascular thrombosis in severe spinal muscular atrophy. *Muscle Nerve* 42: 144–147

Sanchez G, Bondy-Chorney E, Laframboise J, Paris G, Didillon A, Jasmin BJ, Côté J (2015) A novel role for CARM1 in promoting nonsense-mediated mRNA decay: potential implications for spinal muscular atrophy. *Nucleic Acids Res* 44: 2661–2676

Sargsyan SA, Monk PN, Shaw PJ (2005) Microglia as potential contributors to motor neuron injury in amyotrophic lateral sclerosis. *Glia* 51: 241–253

Scaramuzzino C, Casci I, Parodi S, Lievens PMJ, Polanco MJ, Milioto C, Chivet M, Monaghan J, Mishra A, Badders N *et al* (2015) Protein arginine methyltransferase 6 enhances polyglutamine-expanded androgen receptor function and toxicity in spinal and bulbar muscular atrophy. *Neuron* 85: 88–100

Scheer S, Ackloo S, Medina TS, Schapira M, Li F, Ward JA, Lewis AM, Northrop JP, Richardson PL, Kaniskan HÜ *et al* (2019) A chemical biology toolbox to study protein methyltransferases and epigenetic signaling. *Nat Commun* 10: 19

Shafey D, Boyer JG, Bhanot K, Kothary R (2010) Identification of novel interacting protein partners of SMN using tandem affinity purification. *J Proteome Res* 9: 1659–1669

Singh RN, Singh NN (2018) *Mechanism of splicing regulation of spinal muscular atrophy genes*. Cham: Springer

Singh NN, Lawler MN, Ottesen EW, Upreti D, Kaczynski JR, Singh RN (2013) An intronic structure enabled by a long-distance interaction serves as a novel target for splicing correction in spinal muscular atrophy. *Nucleic Acids Res* 41: 8144–8165

Sivaramakrishnan M, McCarthy KD, Campagne S, Huber S, Meier S, Augustin A, Heckel T, Meistermann H, Hug MN, Birrer P *et al* (2017) Binding to SMN2 pre-mRNA-protein complex elicits specificity for small molecule splicing modifiers. *Nat Commun* 8: 1476

Somers E, Lees RD, Hoban K, Sleigh JN, Zhou H, Muntoni F, Talbot K, Gillingwater TH, Parson SH (2016) Vascular defects and spinal cord hypoxia in spinal muscular atrophy. *Ann Neurol* 79: 217–230

Strasswimmer J, Lorson CL, Breiding DE, Chen JJ, Le T, Burghes AHM, Androphy EJ (1999) Identification of survival motor neuron as a transcriptional activator-binding protein. *Hum Mol Genet* 8: 1219–1226

Suárez-Calvet M, Neumann M, Arzberger T, Abou-Ajram C, Funk E, Hartmann H, Edbauer D, Kremmer E, Göbl C, Resch M *et al* (2016) Monomethylated and unmethylated FUS exhibit increased binding to Transportin and distinguish FTLD-FUS from ALS-FUS. *Acta Neuropathol* 131: 587–604

Suraweera A, Lim YC, Woods R, Birrell GW, Nasim T, Becherel OJ, Lavin MF (2009) Functional role for senataxin, defective in ataxia oculomotor apraxia type 2, in transcriptional regulation. *Hum Mol Genet* 18: 3384–3396

Tadesse H, Deschênes-Furry J, Boisvenue S, Côté J (2008) KH-type splicing regulatory protein interacts with survival motor neuron protein and is misregulated in spinal muscular atrophy. *Hum Mol Genet* 17: 506–524

Tisdale S, Lotti F, Saieva L, VanMeerbeke JP, Crawford TO, Sumner CJ, Mentis GZ, Pellizzoni L (2013) SMN is essential for the biogenesis of U7 Small nuclear ribonucleoprotein and 3′-end formation of Histone mRNAs. *Cell Rep* 5: 1187–1195

Vukojicic A, Delestrée N, Fletcher EV, Pagiazitis JG, Sankaranarayanan S, Yednock TA, Barres BA, Mentis GZ (2019) The classical complement pathway mediates microglia-dependent remodeling of spinal motor circuits during development and in SMA. *Cell Rep* 29: 3087–3100.e7

Wang L, Zhao Z, Meyer MB, Saha S, Yu M, Guo A, Wisinski KB, Huang W, Cai W, Pike JW *et al* (2016) CARM1 methylates chromatin remodeling factor BAF155 to enhance tumor progression and metastasis. *Cancer Cell* 30: 179–180

Williamson AR (2000) Creating a structural genomics consortium. *Nat Struct Biol* 7: 953

Wu Q, Heidenreich D, Zhou S, Ackloo S, Krämer A, Nakka K, Lima-Fernandes E, Deblois G, Duan S, Vellanki RN *et al* (2019) A chemical toolbox for the study of bromodomains and epigenetic signaling. *Nat Commun* 10: 1915

Wu Q, Nie DY, Ba-alawi W, Ji Y, Zhang Z, Cruickshank J, Haight J, Ciamponi FE, Chen J, Duan S *et al* (2022) PRMT inhibition induces a viral mimicry response in triple-negative breast cancer. *Nat Chem Biol* 18: 821–830

Xiao R, Tang P, Yang B, Huang J, Zhou Y, Shao C, Li H, Sun H, Zhang Y, Fu XD (2012) Nuclear matrix factor hnRNP U/SAF-A exerts a global control of alternative splicing by regulating U2 snRNP maturation. *Mol Cell* 45: 656–668

Yang Y, Bedford MT (2013) Protein arginine methyltransferases and cancer. *Nat Rev Cancer* 13: 37–50

Yanling Zhao D, Gish G, Braunschweig U, Li Y, Ni Z, Schmitges FW, Zhong G, Liu K, Li W, Moffat J *et al* (2016) SMN and symmetric arginine dimethylation of RNA polymerase II C-terminal domain control termination. *Nature* 529: 48–53

Zhang Z, Lotti F, Dittmar K, Younis I, Wan L, Kasim M, Dreyfuss G (2008) SMN deficiency causes tissue-specific perturbations in the repertoire of snRNAs and widespread defects in splicing. *Cell* 133: 585–600

Zhang R, So BR, Li P, Yong J, Glisovic T, Wan L, Dreyfuss G (2011) Structure of a key intermediate of the SMN complex reveals Gemin2's crucial function in snRNP assembly. *Cell* 146: 384–395

Zhang Z, Pinto AM, Wan L, Wang W, Berg MG, Oliva I, Singh LN, Dengler C, Wei Z, Dreyfuss G (2013) Dysregulation of synaptogenesis genes antecedes motor neuron pathology in spinal muscular atrophy. *Proc Natl Acad Sci USA* 110: 19348–19353

Zheleznyakova GY, Voisin S, Kiselev AV, Sällman Almén M, Xavier MJ, Maretina MA, Tishchenko LI, Fredriksson R, Baranov VS, Schiöth HB (2013) Genome-wide analysis shows association of epigenetic changes in regulators of Rab and Rho GTPases with spinal muscular atrophy severity. *Eur J Hum Genet* 21: 988–993

Zhou C, Feng Z, Ko CP (2016) Defects in motoneuron-astrocyte interactions in spinal muscular atrophy. *J Neurosci* 36: 2543–2553

License: This is an open access article under the terms of the Creative Commons Attribution License, which permits use, distribution and reproduction in any medium, provided the original work is properly cited.

Expanded View Figures

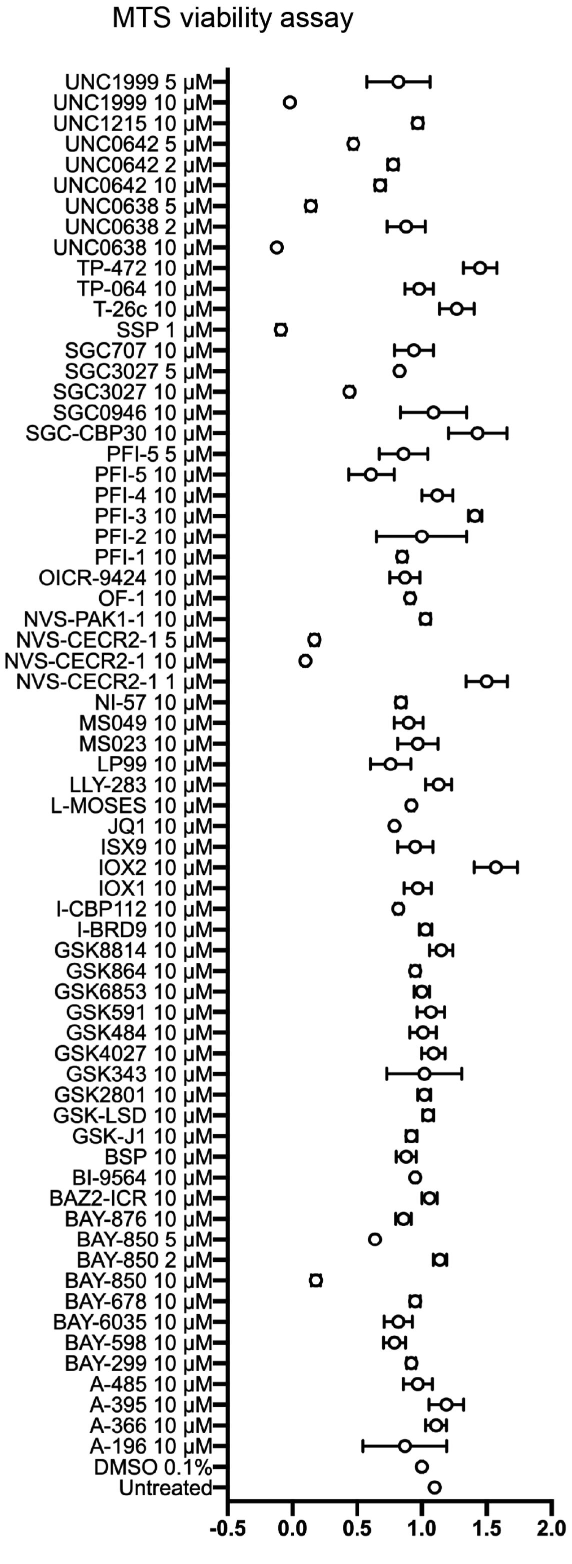

Figure EV1. Effect of epigenetic small molecules on SMA type II patient-derived fibroblast viability.

Viability of cells, assayed by MTS assay, treated with epigenetic small molecules, relative to vehicle-treated cells (0.1% DMSO), normalised to one (n = 2–3). Data are represented as mean ± s.e.m.

© 2023 The Authors

Figure EV2. The increase in *SMN2* exon 7 inclusion by MS023 is specific and does not depend on altered HNRNPA1 levels.

A SMA type II patient-derived fibroblasts were treated with the indicated concentration of MS023 (range: 100 nM–10 μM), (n = 3–4). Cells were harvested for RNA after 48 h incubation. Tot *SMN2* transcript levels relative to *GAPDH* are expressed as fold change compared to untreated SMA fibroblasts, normalised to one. Each dot represents a biological replicate (n = 3–4).

B, C Full-length (FL) SMN2 transcript levels relative to Δ7 SMN2 are expressed as fold change compared to untreated SMA type I and type III fibroblasts, normalised to one. Each dot represents a biological replicate (n = 2–6).

D Western blot showing SMN protein levels upon treatment with increasing MS094 (MS023 negative control) concentrations (top). A representative section of total protein stain, used for protein normalisation, is shown (bottom). The size in kilodalton is indicated on the right.

E Quantification of SMN protein levels relative to total protein is shown. Each dot represents a biological replicate (n = 5).

F Western blot showing vinculin protein (top), HNRNPA1 (middle), and histone 3 (bottom) levels upon treatment with increasing MS023 concentrations and in the cytoplasm (C) and nucleus (N). The size in kilodalton is indicated on the right.

G Quantification of HNRNPA1 protein levels relative to total protein is shown. Each dot represents a biological replicate (n = 4).

Data information: (A), (B), (C), (E), and (G), Data are represented as mean ± s.e.m. and compared with a one-way ANOVA test with multiple comparisons (*$P \leq 0.05$; **$P \leq 0.001$).

 © 2023 The Authors

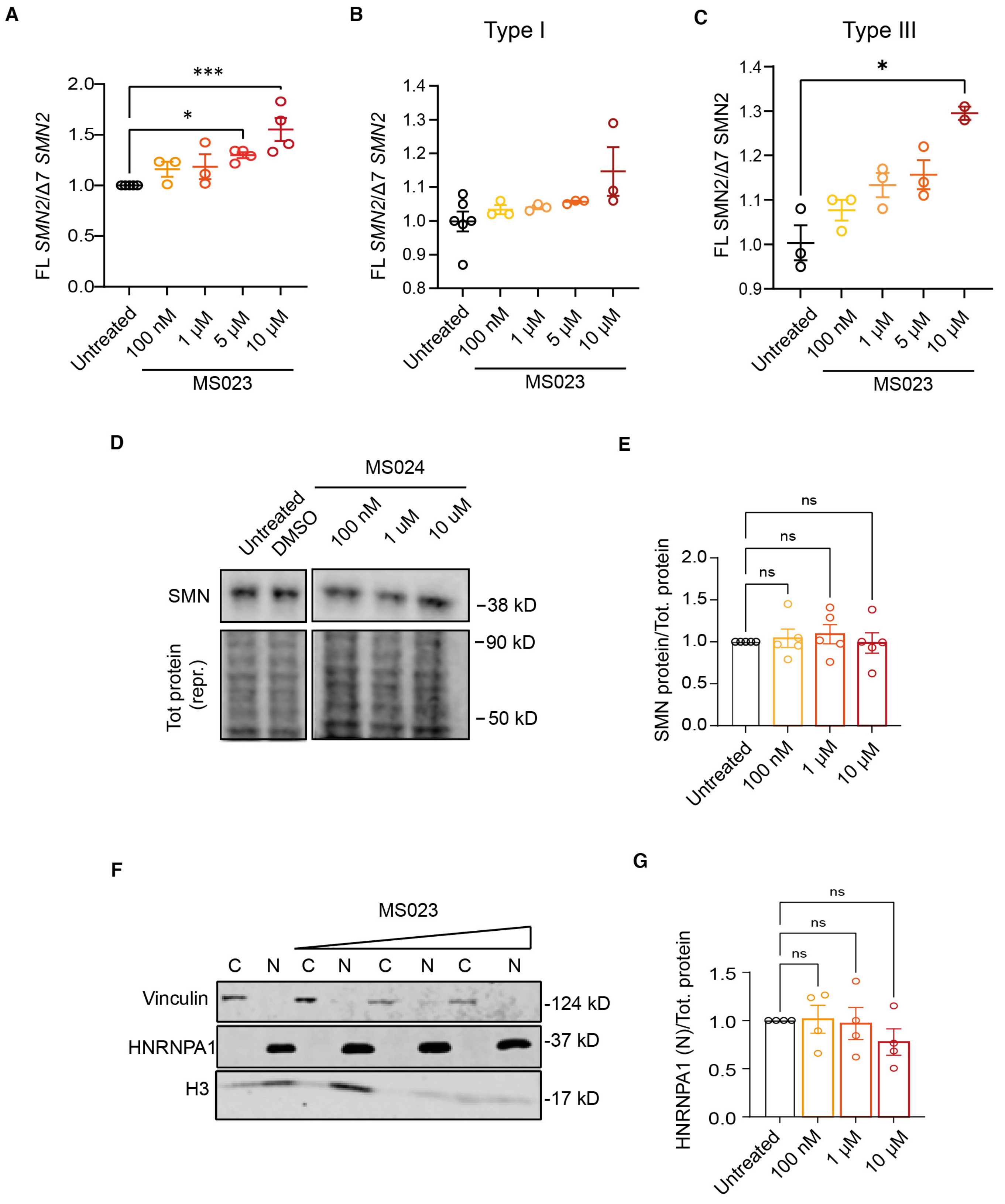
A
***
*
FL SMN2/Δ7 SMN2
2.0
1.5
1.0
0.5
0.0
Untreated
100 nM
1 µM
5 µM
10 µM
MS023
B
Type I
FL SMN2/Δ7 SMN2
1.4
1.3
1.2
1.1
1.0
0.9
0.8
Untreated
100 nM
1 µM
5 µM
10 µM
MS023
C
Type III
*
FL SMN2/Δ7 SMN2
1.4
1.3
1.2
1.1
1.0
0.9
Untreated
100 nM
1 µM
5 µM
10 µM
MS023
D
MS024
Untreated
DMSO
100 nM
1 uM
10 uM
SMN
-38 kD
Tot protein (repr.)
-90 kD
-50 kD
E
ns
ns
ns
SMN protein/Tot. protein
2.0
1.5
1.0
0.5
0.0
Untreated
100 nM
1 µM
10 µM
F
MS023
C N C N C N C N
Vinculin
-124 kD
HNRNPA1
-37 kD
H3
-17 kD
G
ns
ns
ns
HNRNPA1 (N)/Tot. protein
1.5
1.0
0.5
0.0
Untreated
100 nM
1 µM
10 µM

Figure EV2.

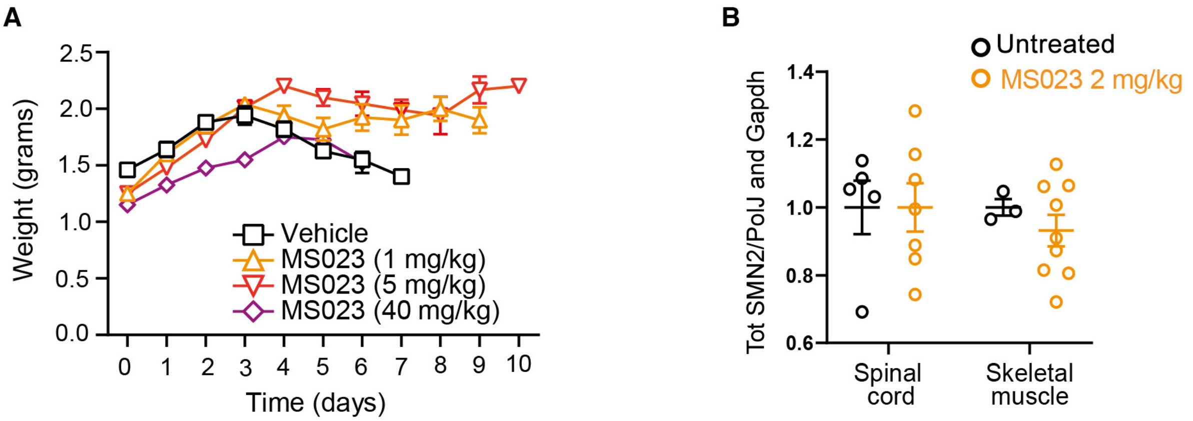

Figure EV3. Oral administration of MS023 improves the phenotype of SMA mice.

A Body weights of untreated (n = 12), MS023- (1 mg/kg: n = 10; 5 mg/kg: n = 9; 40 mg/kg: n = 4), or vehicle-treated (n = 5) SMA mice from postnatal day 0 are shown.
B Tot *SMN2* transcript levels relative to *PolJ* and *Gapdh* in spinal cord and skeletal muscle of treated SMA mice compared to vehicle-treated SMA mice, normalised to one. Each dot represents a biological replicate (n = 10–12).

Data information: (A, B) Data are represented as mean ± s.e.m. and compared with a one-way ANOVA test with multiple comparisons.

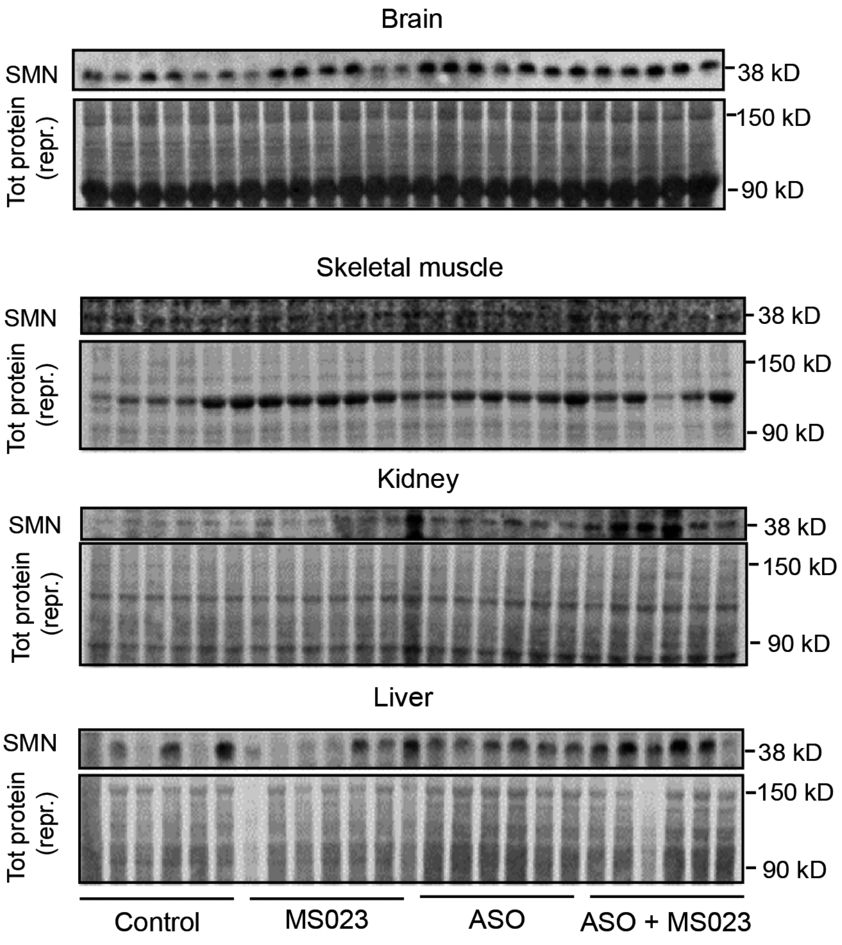

Figure EV4. Combinatorial treatment with MS023 and ASO exerts synergistic effects in SMA mice.

Western blot showing SMN protein levels following the indicated treatments in the brain, skeletal muscle, kidney, and liver of SMA mice (top). A representative section of total protein stain, used for protein normalisation, is also shown (bottom). The size in kilodalton is indicated on the right.

 © 2023 The Authors

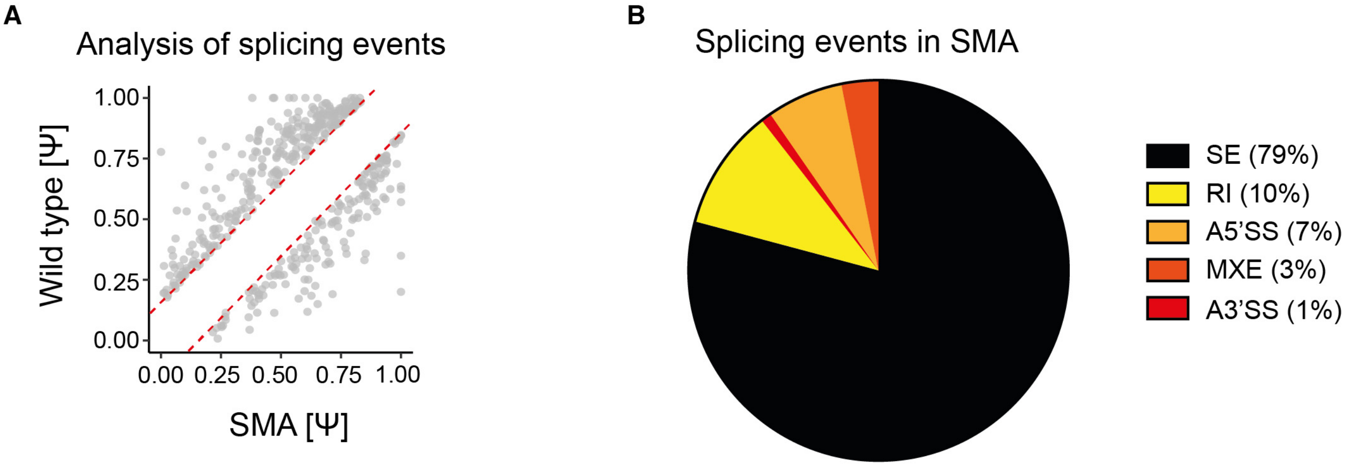

Figure EV5. Altered splicing events in SMA.

A Plot charts show the distribution of 446 aberrant splicing events (Ψ) in SMA mice relative to wild-type littermates. The red dotted lines mark the ±15% normalisation range.

B Pie chart showing the proportion of altered splicing events in the spinal cord of SMA mice compared to wild type (A3′SS, alternative 3′ splice site; A5′SS, alternative 5′ splice site; MXE, mutually exclusive exon; RI, intron retention; SE, skipped exon).

© 2023 The Authors

APPENDIX FIGURES

Table of content:

Appendix Figure S1. Uncropped western blots from Figure 2C.
Appendix Figure S2. Uncropped western blots from Figure 2E.

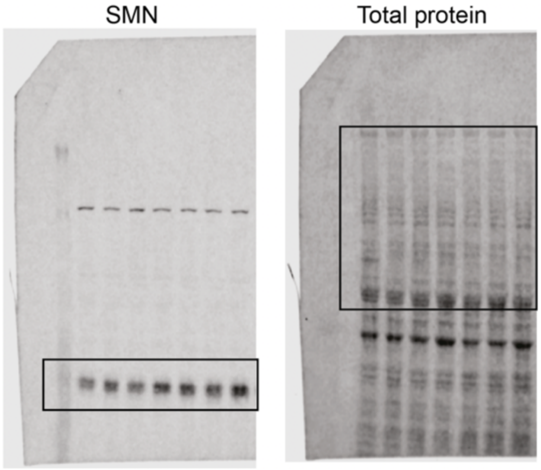

Appendix Figure S1. Uncropped western blots from Figure 2C. The black box indicates the signal corresponding to SMN antibody (left) and total protein area used for quantification (left).

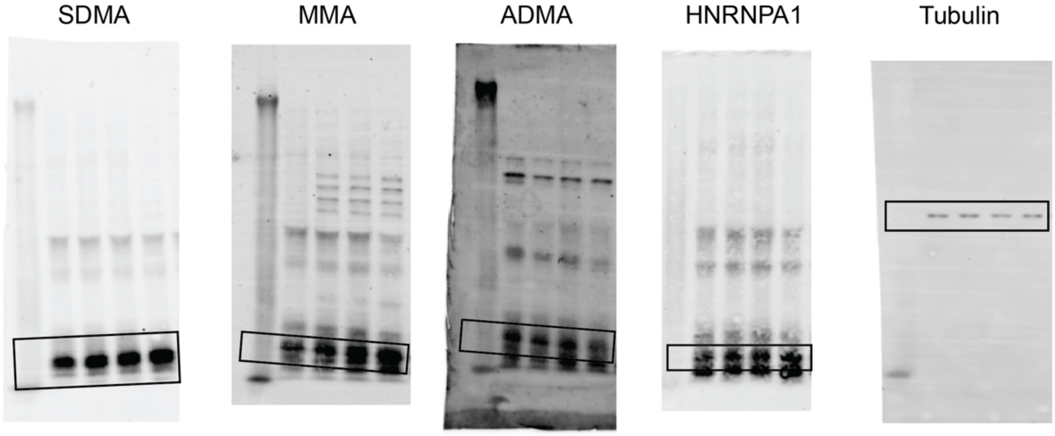

Appendix Figure S2. Uncropped western blots from Figure 2E. The black box indicates the signal corresponding to each antibody.
