# Supplementary figures and images for: PRMT inhibitor promotes SMN2 exon 7 inclusion and synergizes with nusinersen to rescue SMA mice
Source: EMBO Mol Med. 2023 Sep 19;15(11):e17683. doi: 10.15252/emmm.202317683 (PMC10630883; doi:10.15252/emmm.202317683)

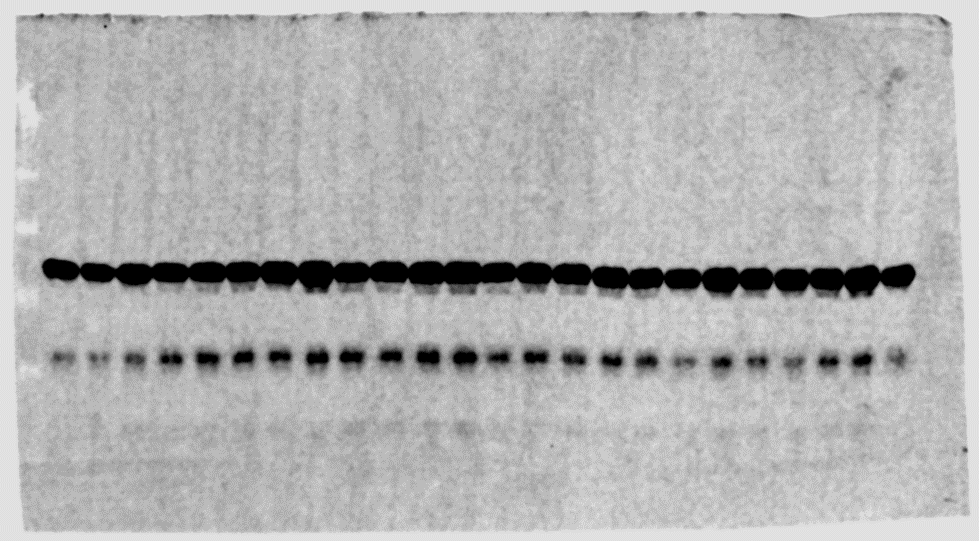

Supplement: Supplementary file 12 — Source Data for Figure 3 [file EMMM-15-e17683-s006.zip › Fig 3 source data/3E SC SMN.png]

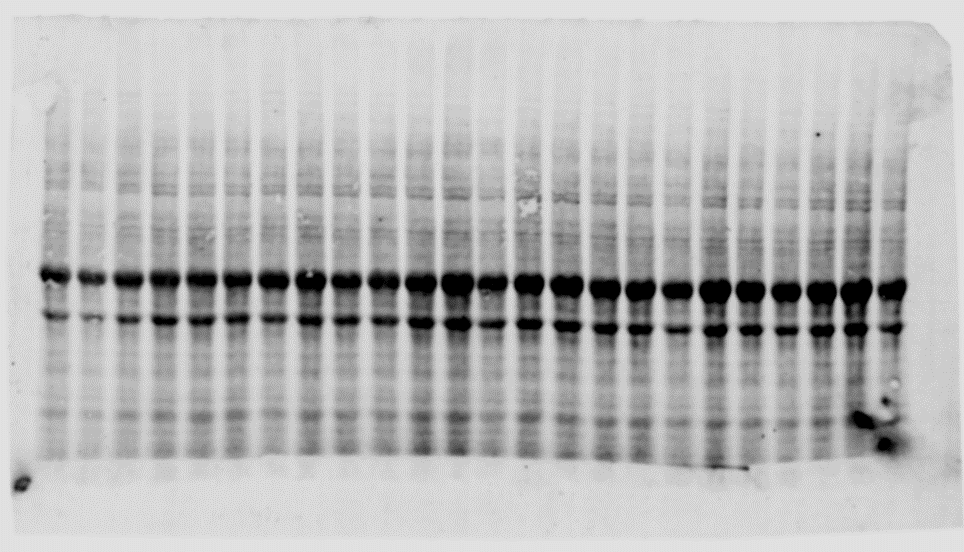

Supplement: Supplementary file 12 — Source Data for Figure 3 [file EMMM-15-e17683-s006.zip › Fig 3 source data/3E SC TOTAL.png]

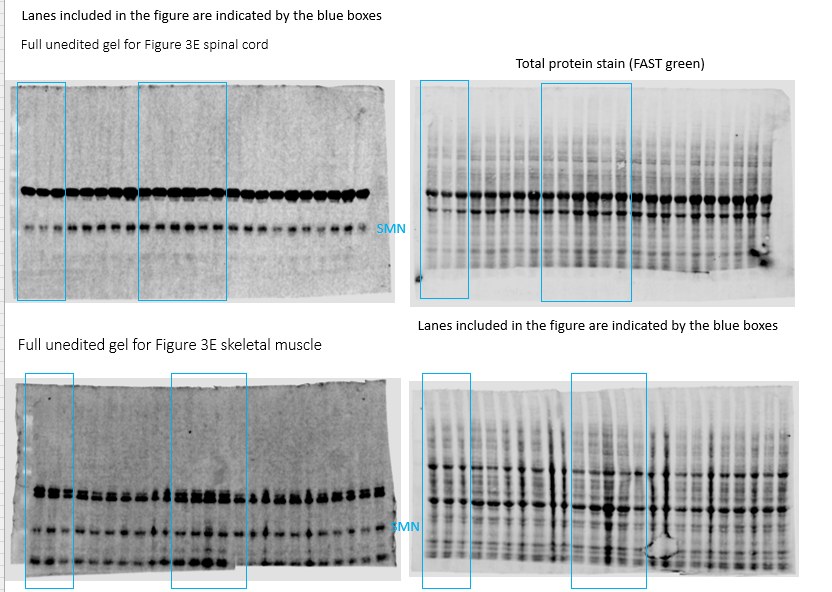

Supplement: Supplementary file 12 — Source Data for Figure 3 [file EMMM-15-e17683-s006.zip › Fig 3 source data/3E with indicated lanes.png]

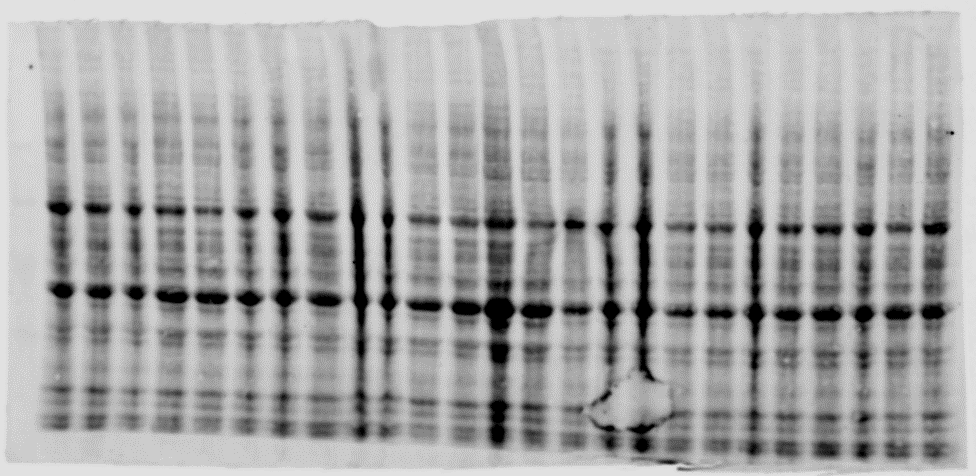

Supplement: Supplementary file 12 — Source Data for Figure 3 [file EMMM-15-e17683-s006.zip › Fig 3 source data/3E SK MUSCLE TOTAL.png]

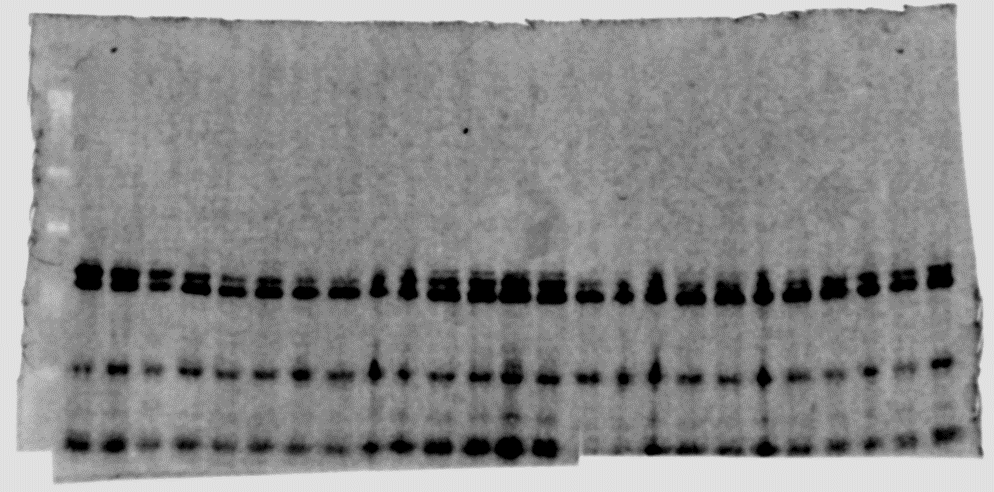

Supplement: Supplementary file 12 — Source Data for Figure 3 [file EMMM-15-e17683-s006.zip › Fig 3 source data/3E SK MUSCLE SMN.png]

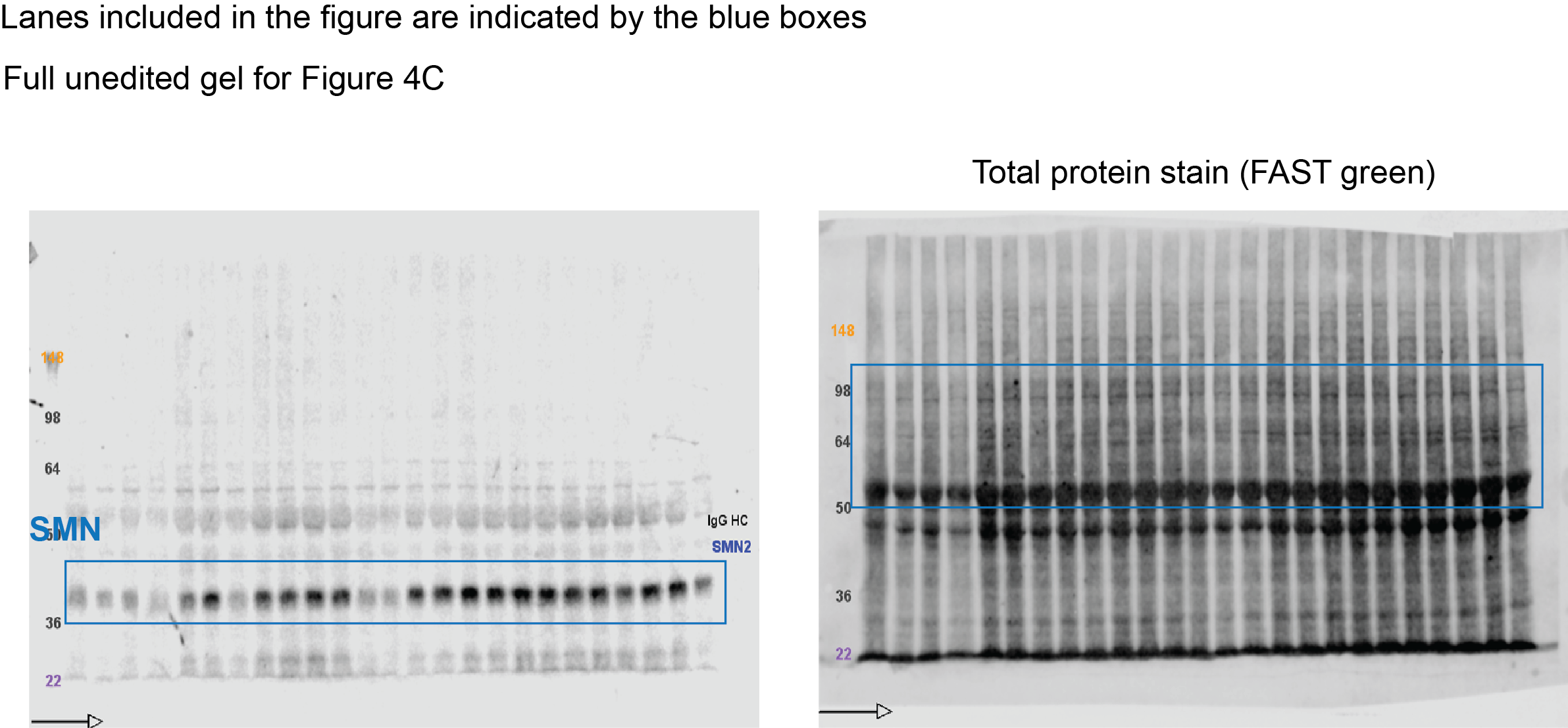

Supplement: Supplementary file 13 — Source Data for Figure 4 [file EMMM-15-e17683-s007.zip › Fig 4 source data/4C with indicated lanes.png]

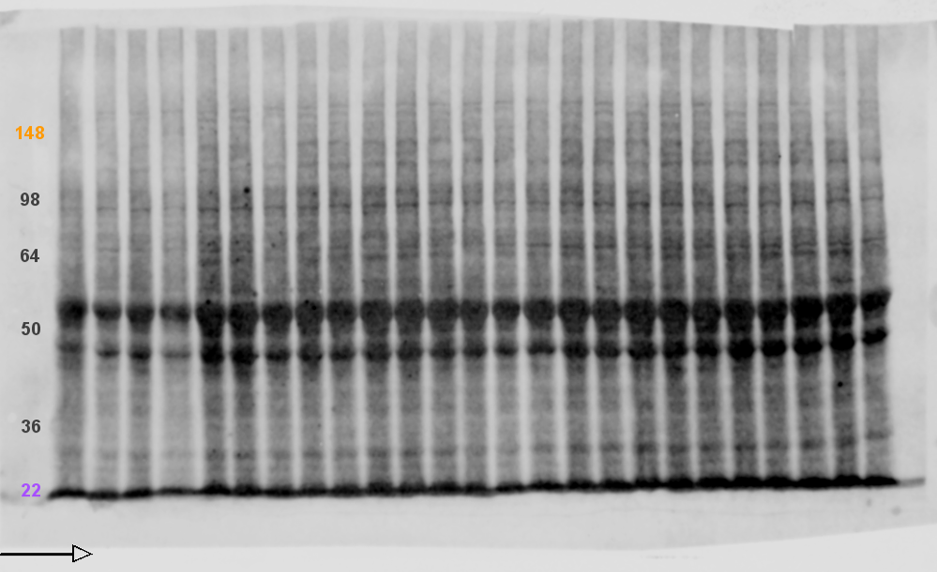

Supplement: Supplementary file 13 — Source Data for Figure 4 [file EMMM-15-e17683-s007.zip › Fig 4 source data/4C TOTAL.png]

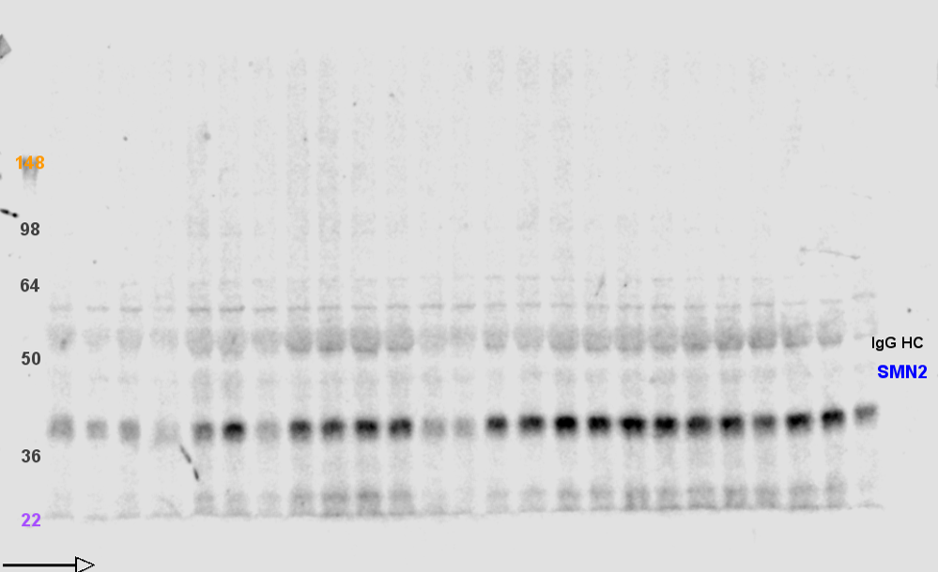

Supplement: Supplementary file 13 — Source Data for Figure 4 [file EMMM-15-e17683-s007.zip › Fig 4 source data/4C SMN.png]
