# Supplementary material for: PRMT inhibitor promotes SMN2 exon 7 inclusion and synergizes with nusinersen to rescue SMA mice
Source: EMBO Mol Med. 2023 Sep 19;15(11):e17683. doi: 10.15252/emmm.202317683 (PMC10630883; doi:10.15252/emmm.202317683)
Supplement: Supplementary file 14 [file EMMM-15-e17683-s003.pdf]

EMBO Molecular Medicine

PRMT inhibitor promotes SMN2 exon 7 inclusion and synergizes with nusinersen to rescue SMA mice

Anna Kordala, Jessica Stoodley, Nina Ahlskog, Muhammad Hanifi, Antonio Garcia Guerra, Amarjit Bhomra, Wooi Fang Lim, Lyndsay Murray, Kevin Talbot, Suzan Hammond, Matthew Wood, and Carlo Rinaldi
DOI: 10.15252/emmm.202317683

Corresponding author: Carlo Rinaldi (carlo.rinaldi@idrm.ox.ac.uk)

Review Timeline:

Transfer from Review Commons:	7th Mar 23
Editorial Decision:	9th Mar 23
Revision Received:	7th Jul 23
Editorial Decision:	9th Aug 23
Revision Received:	31st Aug 23
Accepted:	1st Sep 23

Editor: Zeljko Durdevic

Transaction Report:

This manuscript was transferred to EMBO Molecular Medicine following peer review at Review Commons.

(Note: With the exception of the correction of typographical or spelling errors that could be a source of ambiguity, letters and reports are not edited. Depending on transfer agreements, referee reports obtained elsewhere may or may not be included in this compilation. Referee reports are anonymous unless the Referee chooses to sign their reports.)

Review #1

1. Evidence, reproducibility and clarity:

Evidence, reproducibility and clarity (Required)

Summary

SMA is a severe, monogenetic, progressive neuromuscular disease that mostly affects young children. SMA is cause by homozygous loss of function of SMN1. The main modifier of disease severity - that ranges widely, from neonatal to adult onset - is the presence of a varying number of SMN2 copies in the human genome. Recently, several gene-targeting therapies for SMA have been approved. This has changed the outcome of SMA drastically for many patients, but, surprisingly, the effect of these treatments varies strongly between patients. This leads to significant uncertainty for patients, families and clinicians and poses a challenge to reliable prognosis.

One of the drugs that has been approved for treating SMA is the antisense oligonucleotide nusinersen (Spinraza). Nusinersen improves SMA outcomes by enhancing the splicing of SMN2 -transcribed pre-mRNA, leading to an increase in the inclusion of exon 7 leading to an increase in the availability of full-length, functional SMN protein. In the current manuscript, Kordala and colleagues investigate the effect of a library of 54 compounds (focused on modulating epigenetic regulators) on SMN2 splicing and SMN protein in an SMA type II patient fibroblast cell line. They found the Type I PMRT inhibitor, MS023 to dose-dependently increase full length SMN2 splicing SMN protein levels, by decreasing hnRNPA1 binding to SMN2 pre-mRNA. Next, they show that MS023 monotreatment of the severe 'Taiwanese' (+/- SMA type I) mouse model of SMA leads to improved survival and weight gain. Moreover, they show that combinatorial treatment of the same mouse model with MS023 and nusinersen, significantly further improves survival compared to both nusinersen and MS023 monotreatment. Finally, transcriptome analysis suggests that the majority of misregulated transcripts in SMA is rescued by both nusinersen an combinatorial treatment but, importantly, the rescue observed in the combinatorial group seems more complete.

Suggestions

The authors state in the abstract that "transcriptomic analysis revealed that MS023 treatment has very minimal off-target effects". However, their transcriptomic analyses do not contain a condition that investigates the effects of MS023 on the transcriptome in WT and SMA animals on its own. I belief this would have been an essential addition to support the conclusion on off-target effects of MS023, especially considering the benefits that the authors list in the discussion when compared to e.g. VPA. I agree with their comments about the unspecificity of such drugs; however, I don't belief their current transcriptomics analysis on MS023 fully support this conclusion either. It may not be feasible to include such an experiment in a revised version of the manuscript, but in this case the authors should reflect on the wording of their conclusions.

I agree with the authors that the effect of MS023 on SMN-FL RNA appears to be dose-dependent but I don't think the data fully supports that conclusion for SMN protein levels (compare e.g. 250 nM and 2.5 µM quantification). In fact, there are many inconsistencies between SMN RNA and SMN protein levels: in figure 3 (MS023 monotreatment), the authors observe in spinal cord no change in SMN RNA but a significant increase in SMN protein. In contrast, in the same figure, in muscle, both SMN RNA and protein increase significantly. This is a bit confusing and to me mostly means that the regulation of SMN RNA and protein expression in complex and likely depends on many more factors than PMRT activity and hnRNPA1 arginine methylation status. Indeed, the authors pick hnRNPA1 as a promising target from a list of proteins that contains 72 in total. Are there no other promising candidates in this list that would be able to explain the unclear and inconsistent correlation between SMN RNA splicing and SMN protein levels?

The in vitro work was based on the use of one primary fibroblast cell line. It would be relatively straightforward to characterized the effect of MS023 on e.g. type 1 and type 3 patient-derived lines, thus providing a clearer overview of the use of this type of drug in SMA patients of different types. Both through the Corriell repository (as used in the current paper) and surely also through biobanks at Oxford it should be relatively straightforward to obtain such cell lines and for the authors to extend their analyses to include patients of different types (and with varying SMN2 copy number).

The mechanism that the authors suggest in e.g. Fig. 2D about the interaction of hnRNPA1 with the SMN2-ISSN1 in relation to PMRT inhibition is very similar to how nusinersen prevents SMN2-ISSN1 binding of hnRNPA1 (as the authors mention in the discussion). How do the authors suggest this would work? Do they have suggestions for further experiments to investigate this interaction (e.g. using hnRNPA1 and nusinersen molecules with point mutations?)

Minor comments

Do I understand correctly that none of the screened molecules in figure 1 lead to significantly unregulated SMN protein levels (including MS023)? What causes the difference between figure 1 (no signficicant upregulation of SMN protein) vs figure 2 (a dose dependent increase of SMN protein)? Do the authors have an explanation for this difference? In relation to this point, I am somewhat surprised at the variability in protein quantifications in especially figures 1 and 2. In these figures, biological replicates are obtained from one cell line. Although I understand that there is not necessarily much benefit to including all western blots used for quantification in for example the supplementary files with the paper, it would be useful to see some examples for e.g. the western blots for the quantifications in fig. 1C.
Similarly, I appreciate the complexity of the IPs and arginine-methylation specific blotting in fig 2E, but the current tightly cropped blots are not super convincing and the uncropped blots are not included in the supplementary data. Also how was this quantified; fig 2F lacks some indication of standard deviation or other indicator of reproducibility between measurements.

There are some what appear to be reference formatting errors (e.g. lines 17 and 20 on page 15 of the manuscript PDF amongst others).

The PDF version of Supplementary table 2 in its current format is not really usable or readable; an Excel version would be preferable.

2. Significance:

Significance (Required)

The paper addresses an interesting question: it aims to improve the efficacy of existing drugs for SMA by identifying novel molecules that may improve the working mechanism of, in this case, nusinersen. Others have tried this before by using VPA, but the current molecule appears to be more specific. However, it would have been interesting to get more details on the effect of this novel compound: a wider range of cell lines, further mouse experiments (a control group in figs. 3 and 5) and analysis (e.g. pathological analysis of the neuromuscular system). It would in fact have been interesting to combine some of the analyses in the current work also with the other available SMN2 splicing modifier risdiplam: as risdiplam also modulates SMN2 splicing, MS023 might also have been suitable to improve risdiplam efficacy. Especially in the cell line the authors have used this would have been a relatively straightforward addition. I believe the paper may provide an interesting start, but without further analysis remains at that stage.

The audience to likely be most interest are mostly colleagues from the SMA field, as the mechanisms in the current manuscript focus very much on ISS-N1-regulated SMN2 splicing which is highly specific for SMA.

3. How much time do you estimate the authors will need to complete the suggested revisions:

Estimated time to Complete Revisions (Required)

(Decision Recommendation)

Between 1 and 3 months

4. *Review Commons* values the work of reviewers and encourages them to get credit for their work. Select 'Yes' below to register your reviewing activity at Web of Science Reviewer Recognition Service (formerly Publons); note that the content of your review will not be visible on Web of Science.

Web of Science Reviewer Recognition

Yes

Review #2

1. Evidence, reproducibility and clarity:

Evidence, reproducibility and clarity (Required)

This study by Kordala et al reports the identification of new therapeutics to further improve the clinical outcomes of spinal muscular atrophy (SMA) patients. SMA is childhood neurological disease that is caused by insufficient levels of the survival motor neuron (SMN) protein. There are currently three approved therapies for SMA, yet the disease is not cured and many patients remain severely disabled. The authors conducted a screen of known epigenetic regulators to identify molecules that increase SMN protein. They identified MS023, which is a selectively PRMT inhibitor, that promotes SMN exon 7 inclusion and thus full length SMN protein. Importantly, MS023 improves the SMA phenotype alone or in combination with the SMN2 antisense oligonucleotide suggesting it can potentially be used by itself or in combinatorial approaches. While this is a generally well written paper with relatively straight forward experimental design there remains some concerns that should be addressed.

Major Concerns:

1. The mechanism of action needs further clarification. Does MS023 work similarly to Risdaplam? Also, if Nusinersen is already interfering with hnRNPA1 how does MS023 augment the splicing.
2. MS023 alone did not increase improve exon 7 inclusion in the spinal cord of treated SMA mice yet the protein levels were increased (Fig. 3D,F). Are there alternative mechanisms through which MS023 is acting?
3. Further explanation of why MS023 did not improve exon 7 inclusion in the spinal cord but enhanced the effect of the ASO (Fig. 4B) is needed.
4. Nusinersen has been shown to almost completely rescue the SMA phenotype in mice. Was the dose used here chosen to be suboptimal?

Minor Concern:

Many neurological diseases are now moving to a multimodal approach. The manuscript could be improved with further discussion of why MS023 would be an attractive option compared to other synergistic strategies being employed for SMA, including the most obvious of combining some of the already approved therapies.

2. Significance:

Significance (Required)

This is a generally well done study that works through a screening methodology to identify a molecule that increase the levels of SMN. Mechanistic studies suggest that the compound works through inhibiting the recruitment of hnRNPA1 to the SMN2 gene, thus promoting inclusion of exon 7 and the production of full-length SMN protein.

The study does not provide definitive data that methyl transferase activity of PRMT promotes exon 7 exclusion or that the inhibitor changes the methylation state of any of the proteins involved. However, knockdown experiments does not exclude this possibility.

This study would be of general interest to wider audience if more detail was included regarding the current SMA landscape and how MS023 fits in with what is currently available. The transcriptome data was potentially very interesting since it provided clues on how MS023 is exerting its synergistic effect(neuroinflammation angle is relatively unique), but that data was only briefly discussed.

3. How much time do you estimate the authors will need to complete the suggested revisions:

Estimated time to Complete Revisions (Required)

(Decision Recommendation)

Between 1 and 3 months

4. *Review Commons* values the work of reviewers and encourages them to get credit for their work. Select 'Yes' below to register your reviewing activity at Web of Science Reviewer Recognition Service (formerly Publons); note that the content of your review will not be visible on Web of Science.

Web of Science Reviewer Recognition

Yes

Revision Plan

Manuscript number: RC-2022-01797
Corresponding author: Carlo, Rinaldi

1. General Statements

We thank the reviewers for the time and effort dedicated to carefully assess our manuscript: 'Type I PRMT inhibitor MS023 promotes SMN2 exon 7 inclusion and synergizes with nusinersen to rescue the phenotype of SMA mice' and we are grateful for the positive comments and suggestions provided. As first-time users of the Review Commons platform we have been positively impressed by the high quality and fairness of the assessment.

As correctly pointed out by Reviewer #2, this work would be of general interest to a wide audience for the following reasons: 1) MS023 is a next generation small molecule inhibitor of protein arginine methyltransferases, which are recently being recognized as promising therapeutic targets for a number of human diseases, from cancer to neurodegeneration (Jee Won Hwang, *Experimental & Molecular Medicine*, 2021); and 2) SMA, being at the very forefront of therapeutic development for neurological diseases, is a beacon for the development of combinatorial drug regimens, which is most likely how in the near future most complex diseases would need to be tackled. This study, by assessing the efficacy of MS023 alone and in combination with a clinically approved medicine (nusinersen), demonstrates an added therapeutic benefit of the combination of these two drugs compared to nusinersen alone in SMA models.

2. Description of the planned revisions

Insert here a point-by-point reply that explains what revisions, additional experimentations and analyses are planned to address the points raised by the referees.

Reviewer #1:

A) Their transcriptomic analyses do not contain a condition that investigates the effects of MS023 on the transcriptome in WT and SMA animals on its own. I belief this would have been an essential addition to support the conclusion on off-target effects of MS023, especially considering the benefits that the authors list in the discussion when compared to e.g. VPA.

We fully agree with the Reviewer. This dataset (MS023 alone and comparison with wild type and SMA) is already available, showing correction of a several SMA-related dysregulated pathways and, importantly, a high degree of specificity, which highlights the competitive advantage of this molecule compared to other epigenetic small molecules, such as VPA.

See principal component (PC) analysis below, showing clustering of the following group of samples: untreated, MS023, nusinersen, MS023+nusinersen (MS_nus), and control. This information, together with the full list of differentially expressed gene in SMA mice treated with

MS023 only versus SMA untreated and control littermates, will be included in the revised manuscript.

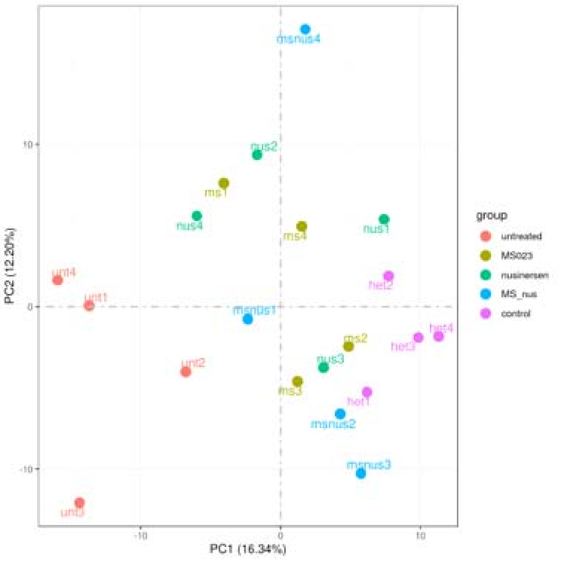

B) There are many inconsistencies between SMN RNA and SMN protein levels

We thank the Reviewer for point this out. We observed no inconsistency in the in vitro data using patient-derived fibroblasts, where treatment with MS023 invariably led to increase exon 7 inclusion and SMN protein expression. This was recapitulated in skeletal muscle of SMA mice, but not in spinal cords, where we only observed an increase in SMN protein expression but not exon 7 inclusion (Fig 3D-F and Fig. 4B-E). We believe that the reason for this discrepancy can be attributed to the fact that: 1) the kinetics of full length and Δ7 *SMN2* mRNA have been shown to increase within 4 hours of both risdiplam and SMN-C2, two splicing inducing small molecules, and return to baseline within 32 hours after both single and repeated dosing, while the SMN protein levels have shown to accumulate following repeated dosing (Naryshkin N, *Science*, 2014; and that 2) spinal cord SMN mRNA expression has been shown to correlate only moderately with protein expression (Ramos DM, *JCI* 2019), overall suggesting that mechanisms other than transcriptional activity or splicing, such as translational efficiency and/or protein stability, may contribute to SMN protein expression in spinal cord and that variations in these other molecular regulators could also account for variations of therapeutic response observed in patients treated with nusinersen (Ramos DM, JCI 2019). We will include these considerations in the discussion of the revised manuscript.

C) The authors pick hnRNPA1 as a promising target from a list of proteins that contains 72 in total. Are there no other promising candidates in this list that would be able to explain the unclear and inconsistent correlation between SMN RNA splicing and SMN protein levels?

A recent paper reported the list of methyl-arginine peptides identified by stable isotope labeling using amino acids in cell culture (SILAC) methodology in human cells upon treatment with

MS023 (Fong JY, *Cancer Cell*, 2019). Out of 72 responsive targets, MS023 changed the methylation pattern of 9 splicing factors reported to affect *SMN2* exon 7 inclusion (*hnRNPA1, hnRNPU, hnRNPA2/B1, HuR, RBM10, Sam68, SRp30c, Tra2-beta1, U2AF35*). We decided to focus on hnRNPA1 because its established role in SMN biology. Nevertheless, our studies do not exclude other mechanisms nor the involvement of other RNA binding proteins (RBP). In order to reinforce the leading role of hnRNPA1 in MS023 mechanism of action, we plan to knock down these candidate RBPs and verify whether the effect of MS023 is preserved. We will discuss these limitations in the discussion in the revised version of the manuscript.

D) It would be relatively straightforward to characterized the effect of MS023 on e.g. type 1 and type 3 patient-derived lines, thus providing a clearer overview of the use of this type of drug in SMA patients of different types…It would have been interesting to get more details on the effect of this novel compound: further mouse experiments (a control group in figs. 3 and 5) and analysis (e.g. pathological analysis of the neuromuscular system).

We have acquired type 1 and type 3 fibroblasts from Prof Talbot (University of Oxford) and we will characterize the effect of MS023 in these patient-derived models. We have agreed to include Prof Talbot in the list of authors as a reflection of this collaboration.

All the functional experiments shown in Fig 3 have been performed using both vehicle-treated and untreated SMA mice as controls. As no differences between the two groups were observed, we opted to only show the vehicle treated arm. We will include in the revised version both controls, as requested by the Reviewer.

E) The mechanism that the authors suggest in e.g. Fig. 2D about the interaction of hnRNPA1 with the SMN2-ISSN1 in relation to PMRT inhibition is very similar to how nusinersen prevents SMN2-ISSN1 binding of hnRNPA1 (as the authors mention in the discussion). How do the authors suggest this would work? Do they have suggestions for further experiments to investigate this interaction (e.g. using hnRNPA1 and nusinersen molecules with point mutations?)

Nusinersen is an antisense oligonucleotide that targets specifically the intronic splicing silencer N1 (ISS-N1) sequence in *SMN2* pre-mRNA, by a proposed mechanism of interference with the binding of negative splicing modulator hnRNP A1/A2 (Hya Y, *Am J Hum Genet.* 2008). hnRNPA1 directly binds *SMN2* pre-mRNA across multiple sites, as you can see from the figure below (extracted from Lejman et al., *Genes* 2021), promoting exon 7 exclusion not only by binding to the ISS-N1 sequence, but also the splicing enhancer (SE) regions 1 and 2 (Pedrotti S, *EMBO J.* 2010).

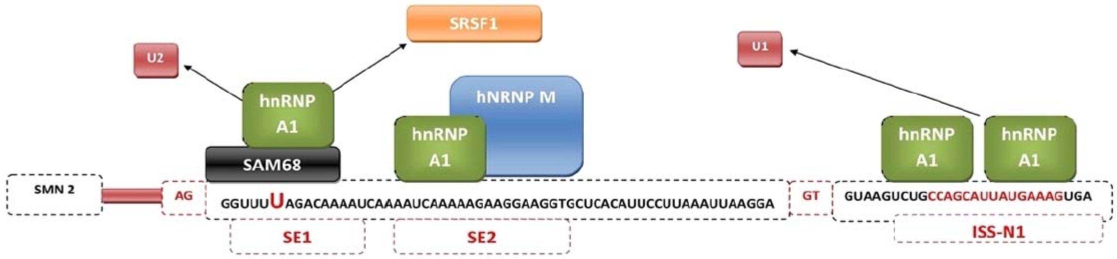

By altering the methylation pattern of hnRNPA1, MS023 affects its *overall* ability to bind *SMN2* mRNA, as shown in Figure 2E-G in the manuscript, therefore resulting in the synergistic effect observed upon co-treatment with nusinersen, which only interferes with binding to the ISS-N1 region. We will include this explanation in the discussion of the manuscript.

In the revised manuscript, we will discuss future experiments to confirm this mechanism of action in the discussion section of the revised paper; those would include: 1) to identify the sites of protein methylation changes in motor neurons by stable isotope labeling using amino acids in cell culture (SILAC) methodology, and 2) to introduce mutations to either abrogate hnRNPA1 activity and/or prevent binding to pre-mRNA *SMN* and verify that the effect of MS023 on splicing is lost.

F) Do I understand correctly that none of the screened molecules in figure 1 lead to significantly unregulated SMN protein levels (including MS023)? … It would be useful to see some examples for e.g. the western blots for the quantifications in fig. 1C. Similarly, I appreciate the complexity of the IPs and arginine-methylation specific blotting in fig 2E, but the current tightly cropped blots are not super convincing and the uncropped blots are not included in the supplementary data.

In the experiment shown in Fig 1C, in red are depicted all the molecules that led to an increase in SMN protein levels. No statistical analysis is shown, as for most targets the experiment was performed in duplicate, given the large number of targets. We decided to follow up with MS023 as it was the only molecule promoting *SMN2* exon 7 inclusion (Fig 1A, B), therefore pointing towards a specific mechanism of action.

We will include the uncropped blots for all the Western blots shown in the paper in the Supplementary data, including the IP from Fig 2E.

G) There are some what appear to be reference formatting errors (e.g. lines 17 and 20 on page 15 of the manuscript PDF amongst others).

These will be corrected in the final version.

G) The PDF version of Supplementary table 2 in its current format is not really usable or readable; an Excel version would be preferable.

Supplementary Table 2 will be provided as an Excel file.

Reviewer #2:

A) The mechanism of action needs further clarification. Does MS023 work similarly to Risdaplam? Also, if Nusinersen is already interfering with hnRNPA1 how does MS023 augment the splicing.

For explanation of the mechanism of action, please see response to question E) of Reviewer #1. Risdiplam's proposed mechanism of action, which has not been entirely elucidated, entails binding on two sites within the exon 7 of the SMN2 transcript, namely exonic splicing enhancer 2 (ESE2) and 5′ splice site (5'ss), leading to dislocation of the hnRNP G allowing the binding of the U1 snRNP complex. As the Reviewer correctly points out, it is expected that MS023 treatment may also enhancer the effect of risdiplam.

B) MS023 alone did not increase improve exon 7 inclusion in the spinal cord of treated SMA mice yet the protein levels were increased (Fig. 3D,F). Are there alternative mechanisms through which MS023 is acting? Further explanation of why MS023 did not improve exon 7 inclusion in the spinal cord but enhanced the effect of the ASO (Fig. 4B) is needed.

We agree with the Reviewer. Please see answer to question B) of Reviewer #1.

C) Nusinersen has been shown to almost completely rescue the SMA phenotype in mice. Was the dose used here chosen to be suboptimal?

Yes: in order to appreciate a synergistic effect we have chosen intentionally a suboptimal dose, based on work previously performed in the Wood lab (Hammond S, *PNAS*, 2016).

3. Description of the revisions that have already been incorporated in the transferred manuscript

Revisions have not yet been incorporated in the transferred manuscript.

4. Description of analyses that authors prefer not to carry out

- The direct comparison between MS023 and risdiplam or testing the combination of MS023 with risdiplam is beyond the scope of the current work. This is based on the consideration that a combinatorial approach between two complementary strategies (nusinersen, delivered intrathecally and specifically targeting motor neurons) and a small molecule (MS023, orally active and with potential to reduce nusinersen dose and frequency of administration while acting on peripheral tissues) is of higher translational value than two overlapping approaches (i.e. two small molecules). The authors recognize the importance of

comparing the combination of MS023 and nusinersen versus risdiplam and nusinersen, and this should be addressed in future studies.

9th Mar 2023

Dear Dr. Rinaldi,

Thank you for the submission of your research manuscript to our editorial offices. I have now had the opportunity to read the manuscript, the referee reports and your point-by-point response and to discuss it with the other members of our editorial team. We all agreed that the manuscript fits the scope of EMBO Molecular Medicine but also that the referees raised important concerns that should be addressed in a major revision. In our opinion your revision plan addresses the referees' criticism adequately and therefore we would like to invite submission of the revised manuscript to our journal.

Further consideration of a revision that addresses reviewers' concerns in full will entail a second round of review. EMBO Molecular Medicine encourages a single round of revision only and therefore, acceptance or rejection of the manuscript will depend on the completeness of your responses included in the next, final version of the manuscript. For this reason, and to save you from any frustrations in the end, I would strongly advise against returning an incomplete revision.

We would welcome the submission of a revised version within three months for further consideration. Please let us know if you require longer to complete the revision.

I look forward to receiving your revised manuscript.

Yours sincerely,

Zeljko Durdevic

Zeljko Durdevic
Editor
EMBO Molecular Medicine

*****

When submitting your revised manuscript, please carefully review the instructions that follow below. We perform an initial quality control of all revised manuscripts before re-review; failure to include requested items will delay the evaluation of your revision.

We require:

1) A .docx formatted version of the manuscript text (including legends for main figures, EV figures and tables). Please make sure that the changes are highlighted to be clearly visible.

2) Individual production quality figure files as .eps, .tif, .jpg (one file per figure). For guidance, download the 'Figure Guide PDF': (https://www.embopress.org/page/journal/17574684/authorguide#figureformat).

3) A .docx formatted letter INCLUDING the reviewers' reports and your detailed point-by-point responses to their comments. As part of the EMBO Press transparent editorial process, the point-by-point response is part of the Review Process File (RPF), which will be published alongside your paper.

4) A complete author checklist, which you can download from our author guidelines (https://www.embopress.org/page/journal/17574684/authorguide#submissionofrevisions). Please insert information in the checklist that is also reflected in the manuscript. The completed author checklist will also be part of the RPF.

5) Please note that all corresponding authors are required to supply an ORCID ID for their name upon submission of a revised manuscript.

6) It is mandatory to include a 'Data Availability' section after the Materials and Methods. Before submitting your revision, primary

datasets produced in this study need to be deposited in an appropriate public database, and the accession numbers and database listed under 'Data Availability'. Please remember to provide a reviewer password if the datasets are not yet public (see https://www.embopress.org/page/journal/17574684/authorguide#dataavailability).

In case you have no data that requires deposition in a public database, please state so in this section. Note that the Data Availability Section is restricted to new primary data that are part of this study.

7) For data quantification: please specify the name of the statistical test used to generate error bars and P values, the number (n) of independent experiments (specify technical or biological replicates) underlying each data point and the test used to calculate p-values in each figure legend. The figure legends should contain a basic description of n, P and the test applied. Graphs must include a description of the bars and the error bars (s.d., s.e.m.). See also 'Figure Legend' guidelines: https://www.embopress.org/page/journal/17574684/authorguide#figureformat

8) At EMBO Press we ask authors to provide source data for the main manuscript figures. Our source data coordinator will contact you to discuss which figure panels we would need source data for and will also provide you with helpful tips on how to upload and organize the files.

9) Our journal encourages inclusion of *data citations in the reference list* to directly cite datasets that were re-used and obtained from public databases. Data citations in the article text are distinct from normal bibliographical citations and should directly link to the database records from which the data can be accessed. In the main text, data citations are formatted as follows: "Data ref: Smith et al, 2001" or "Data ref: NCBI Sequence Read Archive PRJNA342805, 2017". In the Reference list, data citations must be labeled with "[DATASET]". A data reference must provide the database name, accession number/identifiers and a resolvable link to the landing page from which the data can be accessed at the end of the reference. Further instructions are available at .

10) We replaced Supplementary Information with Expanded View (EV) Figures and Tables that are collapsible/expandable online. A maximum of 5 EV Figures can be typeset. EV Figures should be cited as 'Figure EV1, Figure EV2" etc... in the text and their respective legends should be included in the main text after the legends of regular figures.

- For the figures that you do NOT wish to display as Expanded View figures, they should be bundled together with their legends in a single PDF file called *Appendix*, which should start with a short Table of Content. Appendix figures should be referred to in the main text as: "Appendix Figure S1, Appendix Figure S2" etc.

- Additional Tables/Datasets should be labeled and referred to as Table EV1, Dataset EV1, etc. Legends have to be provided in a separate tab in case of .xls files. Alternatively, the legend can be supplied as a separate text file (README) and zipped together with the Table/Dataset file.

See detailed instructions here:

.

11) The paper explained: EMBO Molecular Medicine articles are accompanied by a summary of the articles to emphasize the major findings in the paper and their medical implications for the non-specialist reader. Please provide a draft summary of your article highlighting

- the medical issue you are addressing,

- the results obtained and

- their clinical impact.

This may be edited to ensure that readers understand the significance and context of the research. Please refer to any of our published articles for an example.

12) For more information: There is space at the end of each article to list relevant web links for further consultation by our readers. Could you identify some relevant ones and provide such information as well? Some examples are patient associations, relevant databases, OMIM/proteins/genes links, author's websites, etc...

13) Author contributions: the contribution of every author must be detailed in a separate section (before the acknowledgments).

14) A Conflict of Interest statement should be provided in the main text.

15) Every published paper now includes a 'Synopsis' to further enhance discoverability. Synopses are displayed on the journal

webpage and are freely accessible to all readers. They include a short stand first (maximum of 300 characters, including space) as well as 2-5 one-sentences bullet points that summarizes the paper. Please write the bullet points to summarize the key NEW findings. They should be designed to be complementary to the abstract - i.e. not repeat the same text. We encourage inclusion of key acronyms and quantitative information (maximum of 30 words / bullet point). Please use the passive voice. Please attach these in a separate file or send them by email, we will incorporate them accordingly.

Please also suggest a striking image or visual abstract to illustrate your article as a PNG file 550 px wide x 300-600 px high.

EMBO Molecular Medicine has a "scooping protection" policy, whereby similar findings that are published by others during review or revision are not a criterion for rejection. Should you decide to submit a revised version, I do ask that you get in touch after three months if you have not completed it, to update us on the status.

***** Reviewer's comments *****

***

Rev_Com_number: RC-2022-01797
New_manu_number: EMM-2023-17683
Corr_author: Rinaldi
Title: Type I PRMT inhibitor MS023 promotes SMN2 exon 7 inclusion and synergizes with nusinersen to rescue the phenotype of SMA mice

Revision Plan

Manuscript number: RC-2022-01797
Corresponding author: Carlo, Rinaldi

1. General Statements

We thank the reviewers for the time and effort dedicated to carefully assess our manuscript: ‘Type I PRMT inhibitor MS023 promotes SMN2 exon 7 inclusion and synergizes with nusinersen to rescue the phenotype of SMA mice’ and we are grateful for the positive comments and suggestions provided. As first-time users of the Review Commons platform we have been positively impressed by the high quality and fairness of the assessment.

As correctly pointed out by Reviewer #2, this work would be of general interest to a wide audience for the following reasons: 1) MS023 is a next generation small molecule inhibitor of protein arginine methyltransferases, which are recently being recognized as promising therapeutic targets for a number of human diseases, from cancer to neurodegeneration (Jee Won Hwang, *Experimental & Molecular Medicine*, 2021); and 2) SMA, being at the very forefront of therapeutic development for neurological diseases, is a beacon for the development of combinatorial drug regimens, which is most likely how in the near future most complex diseases would need to be tackled. This study, by assessing the efficacy of MS023 alone and in combination with a clinically approved medicine (nusinersen), demonstrates an added therapeutic benefit of the combination of these two drugs compared to nusinersen alone in SMA models.

Please find below in green the response point-by-point to the reviewers, against the originally submitted Revision Plan, in blue. Again, we thank the Reviewers and the Editorial Team for the consideration and we look forward to hearing in due course.

2. Description of the planned revisions

Insert here a point-by-point reply that explains what revisions, additional experimentations and analyses are planned to address the points raised by the referees.

Reviewer #1:

A) Their transcriptomic analyses do not contain a condition that investigates the effects of MS023 on the transcriptome in WT and SMA animals on its own. I belief this would have been an essential addition to support the conclusion on off-target effects of MS023, especially considering the benefits that the authors list in the discussion when compared to e.g. VPA.

We fully agree with the Reviewer. This dataset (MS023 alone and comparison with wild type and SMA) is already available, showing correction of a several SMA-related dysregulated pathways and, importantly, a high degree of specificity, which highlights the competitive advantage of this molecule compared to other epigenetic small molecules, such as VPA.

See principal component (PC) analysis below, showing clustering of the following group of samples: untreated, MS023, nusinersen, MS023+nusinersen (MS_nus), and control. This information, together with the full list of differentially expressed gene in SMA mice treated with MS023 only versus SMA untreated and control littermates, will be included in the revised manuscript.

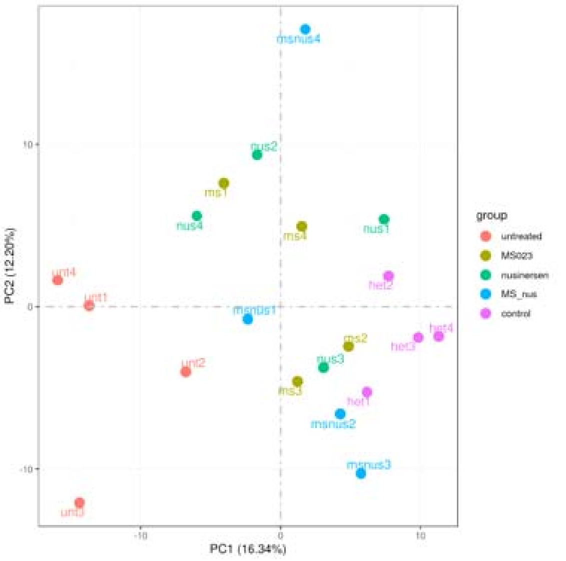

The transcriptomic analysis of spinal cords from P7 SMA mice treated with MS023 alone has been added to the comparisons (Pag. 6, lanes 41-45; Pag. 7, lanes 9-11; and Table EV2 in the revised version). Interestingly, treatment with MS023 alone resulted in correction of 4270 (77.5%) of the 5509 significantly dysregulated transcripts in SMA, with minimal off-target effects, likely due to the very low dose required to achieve *SMN* modulation compared to other applications, as outlined in the Discussion (Pag. 9, lanes 16-20).

B) There are many inconsistencies between SMN RNA and SMN protein levels

We thank the Reviewer for point this out. We observed no inconsistency in the in vitro data using patient-derived fibroblasts, where treatment with MS023 invariably led to increase exon 7 inclusion and SMN protein expression. This was recapitulated in skeletal muscle of SMA mice, but not in spinal cords, where we only observed an increase in SMN protein expression but not exon 7 inclusion (Fig 3D-F and Fig. 4B-E). We believe that the reason for this discrepancy can be attributed to the fact that: 1) the kinetics of full length and Δ7 *SMN2* mRNA have been shown to increase within 4 hours of both risdiplam and SMN-C2, two splicing inducing small molecules, and return to baseline within 32 hours after both single and repeated dosing, while the SMN protein levels have shown to accumulate following repeated dosing (Naryshkin N, *Science*, 2014; and that 2) spinal cord SMN mRNA expression has been shown to correlate only moderately with protein expression (Ramos DM, *JCI* 2019), overall suggesting that mechanisms other than transcriptional activity or splicing, such as translational efficiency and/or protein

stability, may contribute to SMN protein expression in spinal cord and that variations in these other molecular regulators could also account for variations of therapeutic response observed in patients treated with nusinersen (Ramos DM, JCI 2019). We will include these considerations in the discussion of the revised manuscript.
We have included a paragraph in the Discussion section of this revised version, outlining the possible reasons of the discrepancies between *SMN* mRNA and protein levels in spinal cords of SMA mice treated with MS023 (Pag. 7-8, lanes 42-5).

C) The authors pick hnRNPA1 as a promising target from a list of proteins that contains 72 in total. Are there no other promising candidates in this list that would be able to explain the unclear and inconsistent correlation between SMN RNA splicing and SMN protein levels?

A recent paper reported the list of methyl-arginine peptides identified by stable isotope labeling using amino acids in cell culture (SILAC) methodology in human cells upon treatment with MS023 (Fong JY, *Cancer Cell*, 2019). Out of 72 responsive targets, MS023 changed the methylation pattern of 9 splicing factors reported to affect *SMN2* exon 7 inclusion (*hnRNPA1, hnRNPU, hnRNPA2/B1, HuR, RBM10, Sam68, SRp30c, Tra2-beta1, U2AF35*). We decided to focus on hnRNPA1 because its established role in SMN biology. Nevertheless, our studies do not exclude other mechanisms nor the involvement of other RNA binding proteins (RBP). In order to reinforce the leading role of hnRNPA1 in MS023 mechanism of action, we plan to knock down these candidate RBPs and verify whether the effect of MS023 is preserved. We will discuss these limitations in the discussion in the revised version of the manuscript.

We tested silencing of the splicing factors and observed significant toxicity for most of those in the SMA cells, confirming the fundamental role of these proteins in cellular homeostasis and preventing to draw conclusions from this experiment over the individual contributions on MS023 mechanism of action. In this revised version of the manuscript we have listed the 8 other factors implicated in *SMN2* splicing, whose methylation profiles have been shown to change upon MS023 treatment in human cells by methylome analysis (Fong et al. 2019). In the Discussion section of this revised version, we explain the reasons why we decided to investigate the effects of MS023 on HNRNPA1 among those (i.e. established and fundamental role in SMN splicing regulation and putative target for nusinersen mechanism of action). Additionally, we also outline the fact that the individual contributions of these splicing factors remain to be investigated as a limitation of the study and propose as a way forward that this question is addressed by use of advanced techniques such as heavy methyl SILAC and label-free surface plasmon resonance (Pag. 8-9, lanes 36-7).

D) It would be relatively straightforward to characterized the effect of MS023 on e.g. type 1 and type 3 patient-derived lines, thus providing a clearer overview of the use of this type of drug in SMA patients of different types…It would have been interesting to get more details on the effect of this novel compound: further mouse experiments (a control group in figs. 3 and 5) and analysis (e.g. pathological analysis of the neuromuscular system).

We have acquired type 1 and type 3 fibroblasts from Prof Talbot (University of Oxford) and we will characterize the effect of MS023 in these patient-derived models. We have agreed to include Prof Talbot in the list of authors as a reflection of this collaboration.

All the functional experiments shown in Fig 3 and Fig 5 have been performed using unaffected mice ($Smn^{+/-}$; $hSMN2^{+/-}$) as controls. We will include in the revised version of this manuscript, as requested by the Reviewer.

We have acquired fibroblasts derived from patients with type I and type III SMA and confirmed a dose-response effect upon treatment with MS023, suggesting that this small molecule has therapeutic potential for all types of SMA patients. We have also included in Figure 3B, 3C, 4F, and 4G of this revised version the control arm of unaffected mice ($Smn^{+/-}$; $hSMN2^{+/-}$) as reference.

E) The mechanism that the authors suggest in e.g. Fig. 2D about the interaction of hnRNPA1 with the SMN2-ISSN1 in relation to PMRT inhibition is very similar to how nusinersen prevents SMN2-ISSN1 binding of hnRNPA1 (as the authors mention in the discussion). How do the authors suggest this would work? Do they have suggestions for further experiments to investigate this interaction (e.g. using hnRNPA1 and nusinersen molecules with point mutations?)

Nusinersen is an antisense oligonucleotide that targets specifically the intronic splicing silencer N1 (ISS-N1) sequence in *SMN2* pre-mRNA, by a proposed mechanism of interference with the binding of negative splicing modulator hnRNP A1/A2 (Hya Y, *Am J Hum Genet.* 2008). hnRNPA1 directly binds *SMN2* pre-mRNA across multiple sites, as you can see from the figure below (extracted from Lejman et al., *Genes* 2021), promoting exon 7 exclusion not only by binding to the ISS-N1 sequence, but also the splicing enhancer (SE) regions 1 and 2 (Pedrotti S, *EMBO J.* 2010).

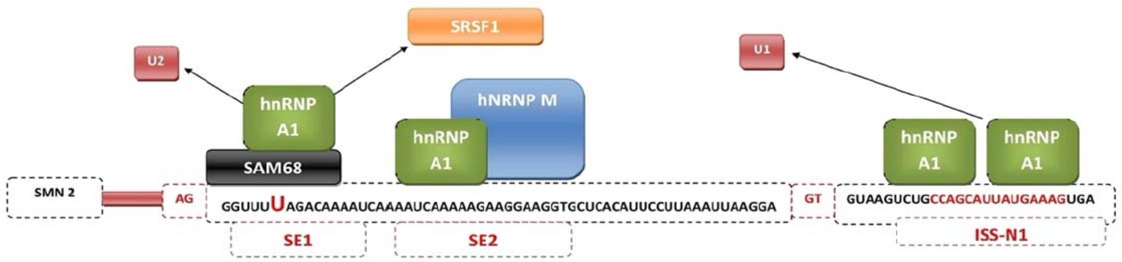

By altering the methylation pattern of hnRNPA1, MS023 affects its *overall* ability to bind *SMN2* mRNA, as shown in Figure 2E-G in the manuscript, therefore resulting in the synergistic effect observed upon co-treatment with nusinersen, which only interferes with binding to the ISS-N1 region. We will include this explanation in the discussion of the manuscript.

In the revised manuscript, we will discuss future experiments to confirm this mechanism of action in the discussion section of the revised paper; those would include: 1) to identify the sites of protein methylation changes in motor neurons by stable isotope labeling using amino acids in cell culture (SILAC) methodology, and 2) to introduce mutations to either abrogate hnRNPA1 activity and/or prevent binding to pre-mRNA *SMN* and verify that the effect of MS023 on splicing is lost.

The proposed mechanism of action for MS023 and its synergistic effect with nusinersen have been added the Discussion of this revised version (Pag. 8-9, lanes 36-7), together with suggestions for further experiments to corroborate and expand our findings.

F) Do I understand correctly that none of the screened molecules in figure 1 lead to significantly unregulated SMN protein levels (including MS023)? … It would be useful to see some examples for e.g. the western blots for the quantifications in fig. 1C. Similarly, I appreciate the complexity of the IPs and arginine-methylation specific blotting in fig 2E, but the current tightly cropped blots are not super convincing and the uncropped blots are not included in the Expanded View data.

In the experiment shown in Fig 1C, in red are depicted all the molecules that led to an increase in SMN protein levels. No statistical analysis is shown, as for most targets the experiment was performed in duplicate, given the large number of targets. We decided to follow up with MS023 as it was the only molecule promoting *SMN2* exon 7 inclusion (Fig 1A, B), therefore pointing towards a specific mechanism of action.

We will include the uncropped blots for all the Western blots shown in the paper in the Supplementary data, including the IP from Fig 2E.

We have included in Appendix (S1) a representative uncropped blot referring to the western blot shown in Figure 2C, showing the increase in SMN protein levels upon treatment of SMA-patient derived fibroblasts with increasing concentrations of MS023.
We have also included in Appendix (S2) the uncropped blots referring to the IP data shown in Fig. 2E.

G) There are some what appear to be reference formatting errors (e.g. lines 17 and 20 on page 15 of the manuscript PDF amongst others).

These will be corrected in the final version.

Reference formatting errors have been amended.

G) The PDF version of Supplementary table 2 in its current format is not really usable or readable; an Excel version would be preferable.

Supplementary Table 2 will be provided as an Excel file.

Expanded Tables are provided as excel files in this revised version.

Reviewer #2:

A) The mechanism of action needs further clarification. Does MS023 work similarly to Risdaplam? Also, if Nusinersen is already interfering with hnRNPA1 how does MS023 augment the splicing.

For explanation of the mechanism of action, please see response to question E) of Reviewer #1. Risdiplam's proposed mechanism of action, which has not been entirely elucidated, entails binding on two sites within the exon 7 of the SMN2 transcript, namely exonic splicing enhancer 2 (ESE2) and 5′ splice site (5'ss), leading to dislocation of the hnRNP G allowing the binding of the U1 snRNP complex. As the Reviewer correctly points out, it is expected that MS023 treatment may also enhancer the effect of risdiplam.

We have further explained the proposed mechanism of action of MS023 and outlined the differences with risdplam's proposed mechanism of action in the Discussion of this revised version (Pag. 8-9, lanes 36-7).

B) MS023 alone did not increase improve exon 7 inclusion in the spinal cord of treated SMA mice yet the protein levels were increased (Fig. 3D,F). Are there alternative mechanisms through which MS023 is acting? Further explanation of why MS023 did not improve exon 7 inclusion in the spinal cord but enhanced the effect of the ASO (Fig. 4B) is needed.

We agree with the Reviewer. Please see answer to question B) of Reviewer #1.

We have included a paragraph in the Discussion section of this revised version, outlining the possible reasons of the discrepancies between *SMN* mRNA and protein levels in spinal cords of SMA mice treated with MS023 (Pag. 7-8, lanes 42-5).

C) Nusinersen has been shown to almost completely rescue the SMA phenotype in mice. Was the dose used here chosen to be suboptimal?

Yes: in order to appreciate a synergistic effect we have chosen intentionally a suboptimal dose, based on work previously performed in the Wood lab (Hammond S, *PNAS*, 2016).

This has been further clarified in the text (Pag. 6, lanes 22-23).

3. Description of the revisions that have already been incorporated in the transferred manuscript

Revision Plan

Revisions have not yet been incorporated in the transferred manuscript.

4. Description of analyses that authors prefer not to carry out

- The direct comparison between MS023 and risdiplam or testing the combination of MS023 with risdiplam is beyond the scope of the current work. This is based on the consideration that a combinatorial approach between two complementary strategies (nusinersen, delivered intrathecally and specifically targeting motor neurons) and a small molecule (MS023, orally active and with potential to reduce nusinersen dose and frequency of administration while acting on peripheral tissues) is of higher translational value than two overlapping approaches (i.e. two small molecules). The authors recognize the importance of comparing the combination of MS023 and nusinersen versus risdiplam and nusinersen, and this should be addressed in future studies.

9th Aug 2023

Dear Dr. Rinaldi,

Thank you for the submission of your revised manuscript to EMBO Molecular Medicine. I am pleased to inform you that we will be able to accept your manuscript pending the following final amendments:

1) Figures: Main figures and EV figures should be uploaded as individual high-resolution files and only the legends should be in the manuscript text. Panel A should be removed from Fig EV1 and 4 as they only have one panel.
2) In the main manuscript file, please do the following:
- Correct/answer the track changes suggested by our data editors by working from the attached document.
- Add up to 5 keywords.
- Remove data not shown (p. 5).
- Author contributions: Please remove it from the manuscript and specify author contributions in our submission system. CRediT has replaced the traditional author contributions section because it offers a systematic machine-readable author contributions format that allows for more effective research assessment. You are encouraged to use the free text boxes beneath each contributing author's name to add specific details on the author's contribution. More information is available in our guide to authors:
https://www.embopress.org/page/journal/17574684/authorguide#authorshipguidelines
- Please rename "Conflict of Interest" to "Disclosure Statement & Competing Interests" and place it after "Acknowledgements". We updated our journal's competing interests policy in January 2022 and request authors to consider both actual and perceived competing interests. Please review the policy https://www.embopress.org/competing-interests and update your competing interests if necessary.
- Please rename "Data and materials availability" to "Data availability" place it after M&M sectio and remove the sentence All data needed to evaluate the conclusions in the paper are present in the paper and/or the Expanded View Figures and Tables. Please use the following format to report the accession number of your data:

The datasets produced in this study are available in the following databases:
[data type]: [full name of the resource] [accession number/identifier] ([doi or URL or identifiers.org/DATABASE:ACCESSION])

Please check "Author Guidelines" for more information.
https://www.embopress.org/page/journal/17574684/authorguide#availabilityofpublishedmaterial
- Correct the reference citation in the reference list. Author's last name should be followed by initials of first names. Please remove DOIs of published articles. Please check "Author Guidelines" for more information.
https://www.embopress.org/page/journal/17574684/authorguide#referencesformat
3) Tables: Tables EV2, 3, 4 should be renamed Dataset EV1, 2, 3 and their callouts should be updated. Please upload EV tables 1, 5 and 6 as separate .xls files, renamed them to Tables EV1, 2, 3 and update their callouts in the main manuscript text.
4) The Paper Explained: Please provide "The Paper Explained" and add it to the main manuscript text. Please check "Author Guidelines" for more information. https://www.embopress.org/page/journal/17574684/authorguide#researcharticleguide
5) Synopsis: Every published paper now includes a 'Synopsis' to further enhance discoverability. Synopses are displayed on the journal webpage and are freely accessible to all readers. They include separate synopsis image and synopsis text.
- Synopsis image: Please provide the visual abstract as a high-resolution jpeg file 550 px-wide x (250-400)-px high.
- Synopsis text: Please provide a short standfirst (maximum of 300 characters, including space) as well as 2-5 one sentence bullet points that summarise the paper as a .doc file. Please write the bullet points to summarise the key NEW findings. They should be designed to be complementary to the abstract - i.e. not repeat the same text. We encourage inclusion of key acronyms and quantitative information (maximum of 30 words / bullet point). Please use the passive voice.
- Please check your synopsis text and image before submission with your revised manuscript. Please be aware that in the proof stage minor corrections only are allowed (e.g., typos).
6) For more information: This space should be used to list relevant web links for further consultation by our readers. Could you identify some relevant ones and provide such information as well? Some examples are patient associations, relevant databases, OMIM/proteins/genes links, author's websites, etc...
7) As part of the EMBO Publications transparent editorial process initiative (see our Editorial at http://embomolmed.embopress.org/content/2/9/329), EMBO Molecular Medicine will publish online a Review Process File (RPF) to accompany accepted manuscripts. This file will be published in conjunction with your paper and will include the anonymous referee reports, your point-by-point response and all pertinent correspondence relating to the manuscript. Let us know whether you agree with the publication of the RPF and as here, if you want to remove or not any figures from it prior to publication. Please note that the Authors checklist will be published at the end of the RPF.
8) Please provide a point-by-point letter INCLUDING my comments as well as the reviewer's reports and your detailed responses (as Word file).

I look forward to reading a new revised version of your manuscript as soon as possible.

Yours sincerely,
Zeljko Durdevic

Yours sincerely,

Zeljko Durdevic

Zeljko Durdevic
Editor
EMBO Molecular Medicine

*** Instructions to submit your revised manuscript ***

*** PLEASE NOTE *** As part of the EMBO Publications transparent editorial process initiative (see our Editorial at https://www.embopress.org/doi/pdf/10.1002/emmm.201000094), EMBO Molecular Medicine will publish online a Review Process File to accompany accepted manuscripts.

In the event of acceptance, this file will be published in conjunction with your paper and will include the anonymous referee reports, your point-by-point response and all pertinent correspondence relating to the manuscript. If you do NOT want this file to be published, please inform the editorial office at contact@embomolmed.org.

When submitting your revised manuscript, please include:

1) a .docx formatted version of the manuscript text (including Figure legends and tables)

2) Separate figure files*

3) supplemental information as Expanded View and/or Appendix. Please carefully check the authors guidelines for formatting Expanded view and Appendix figures and tables at
https://www.embopress.org/page/journal/17574684/authorguide#expandedview

4) a letter INCLUDING the reviewer's reports and your detailed responses to their comments (as Word file).

5) The paper explained: EMBO Molecular Medicine articles are accompanied by a summary of the articles to emphasize the major findings in the paper and their medical implications for the non-specialist reader. Please provide a draft summary of your article highlighting
- the medical issue you are addressing,
- the results obtained and
- their clinical impact.
This may be edited to ensure that readers understand the significance and context of the research.
Please refer to any of our published articles for an example.

6) For more information: There is space at the end of each article to list relevant web links for further consultation by our readers. Could you identify some relevant ones and provide such information as well? Some examples are patient associations, relevant databases, OMIM/proteins/genes links, author's websites, etc...

7) Author contributions: the contribution of every author must be detailed in a separate section.

8) EMBO Molecular Medicine now requires a complete author checklist (https://www.embopress.org/page/journal/17574684/authorguide) to be submitted with all revised manuscripts. Please use the checklist as guideline for the sort of information we need WITHIN the manuscript. The checklist should only be filled with page numbers were the information can be found. This is particularly important for animal reporting, antibody dilutions (missing) and

exact values and n that should be indicted instead of a range.

9) Every published paper now includes a 'Synopsis' to further enhance discoverability. Synopses are displayed on the journal webpage and are freely accessible to all readers. They include a short stand first (maximum of 300 characters, including space) as well as 2-5 one sentence bullet points that summarise the paper. Please write the bullet points to summarise the key NEW findings. They should be designed to be complementary to the abstract - i.e. not repeat the same text. We encourage inclusion of key acronyms and quantitative information (maximum of 30 words / bullet point). Please use the passive voice. Please attach these in a separate file or send them by email, we will incorporate them accordingly.

You are also welcome to suggest a striking image or visual abstract to illustrate your article. If you do please provide a jpeg file 550 px-wide x 400-px high.

10) A Conflict of Interest statement should be provided in the main text

11) Please note that we now mandate that all corresponding authors list an ORCID digital identifier. This takes <90 seconds to complete. We encourage all authors to supply an ORCID identifier, which will be linked to their name for unambiguous name identification.

Currently, our records indicate that the ORCID for your account is 0000-0002-8307-6638.

Please click the link below to modify this ORCID:
Link Not Available

12) The system will prompt you to fill in your funding and payment information. This will allow Wiley to send you a quote for the article processing charge (APC) in case of acceptance. This quote takes into account any reduction or fee waivers that you may be eligible for. Authors do not need to pay any fees before their manuscript is accepted and transferred to our publisher.

*Additional important information regarding Figures

Each figure should be given in a separate file and should have the following resolution:
Graphs 800-1,200 DPI
Photos 400-800 DPI
Colour (only CMYK) 300-400 DPI"

Figures are not edited by the production team. All lettering should be the same size and style; figure panels should be indicated by capital letters (A, B, C etc). Gridlines are not allowed except for log plots. Figures should be numbered in the order of their appearance in the text with Arabic numerals. Each Figure must have a separate legend and a caption is needed for each panel.

*Additional important information regarding figures and illustrations can be found at https://bit.ly/EMBOPressFigurePreparationGuideline. See also figure legend preparation guidelines: https://www.embopress.org/page/journal/17574684/authorguide#figureformat

The system will prompt you to fill in your funding and payment information. This will allow Wiley to send you a quote for the article processing charge (APC) in case of acceptance. This quote takes into account any reduction or fee waivers that you may be eligible for. Authors do not need to pay any fees before their manuscript is accepted and transferred to our publisher.

***** Reviewer's comments *****

Referee #1 (Comments on Novelty/Model System for Author):

Nothing to add to my original Review Commons comments.

Referee #1 (Remarks for Author):

The author's have done an extensive revision of the manuscript. They have included extensive additional analyses, experiments and added data that address many of my previous comments. I would be happy for the paper to be accepted in this form.

Referee #2 (Comments on Novelty/Model System for Author):

The most relevant models were used for this study.

Referee #2 (Remarks for Author):

The authors have satisfactorily responded to the previous concerns and the manuscript is suitable for publication. This will be a welcomed addition to the SMA literature and expands on the potential therapeutic avenues for improving disease outcomes.

The authors addressed the minor editorial issues.

1st Sep 2023

Dear Dr. Rinaldi,

We are pleased to inform you that your manuscript is accepted for publication and is now being sent to our publisher to be included in the next available issue of EMBO Molecular Medicine.

Please read below for additional IMPORTANT information regarding your article, its publication and the production process.

Congratulations on your interesting work,

Zeljko Durdevic

Zeljko Durdevic
Editor
EMBO Molecular Medicine

Follow us on Twitter @EmboMolMed
Sign up for eTOCs at embopress.org/alertsfeeds

*** *** *** IMPORTANT INFORMATION *** *** ***

SPEED OF PUBLICATION
The journal aims for rapid publication of papers, using using the advance online publication "Early View" to expedite the process: A properly copy-edited and formatted version will be published as "Early View" after the proofs have been corrected. Please help the Editors and publisher avoid delays by providing e-mail address(es), telephone and fax numbers at which author(s) can be contacted.

Should you be planning a Press Release on your article, please get in contact with embomolmed@wiley.com as early as possible, in order to coordinate publication and release dates.

LICENSE AND PAYMENT:

All articles published in EMBO Molecular Medicine are fully open access: immediately and freely available to read, download and share.

EMBO Molecular Medicine charges an article processing charge (APC) to cover the publication costs. You, as the corresponding author for this manuscript, should have already received a quote with the article processing fee separately. Please let us know in case this quote has not been received.

Once your article is at Wiley for editorial production you will receive an email from Wiley's Author Services system, which will ask you to log in and will present you with the publication license form for completion. Within the same system the publication fee can be paid by credit card, an invoice, pro forma invoice or purchase order can be requested.

Payment of the publication charge and the signed Open Access Agreement form must be received before the article can be published online.

PROOFS

You will receive the proofs by e-mail approximately 2 weeks after all relevant files have been sent o our Production Office. Please return them within 48 hours and if there should be any problems, please contact the production office at embopressproduction@wiley.com.

Please inform us if there is likely to be any difficulty in reaching you at the above address at that time. Failure to meet our

deadlines may result in a delay of publication.

All further communications concerning your paper proofs should quote reference number EMM-2023-17683-V3 and be directed to the production office at embopressproduction@wiley.com.

Thank you,

Zeljko Durdevic
Editor
EMBO Molecular Medicine

EMBO Press Author Checklist

Corresponding Author Name: Carlo Rinaldi
Journal Submitted to: EMBO Molecular Medicine
Manuscript Number: EMM-2023-17683, RC-2022-01797

USEFUL LINKS FOR COMPLETING THIS FORM
The EMBO Journal - Author Guidelines
EMBO Reports - Author Guidelines
Molecular Systems Biology - Author Guidelines
EMBO Molecular Medicine - Author Guidelines

Reporting Checklist for Life Science Articles (updated January
This checklist is adapted from Materials Design Analysis Reporting (MDAR) Checklist for Authors. MDAR establishes a minimum set of requirements in transparent reporting in the life sciences (see Statement of Task: 10.31222/osf.io/9sm4x). Please follow the journal's guidelines in preparing your
Please note that a copy of this checklist will be published alongside your article.

Abridged guidelines for figures
1. Data
The data shown in figures should satisfy the following conditions:
- the data were obtained and processed according to the field's best practice and are presented to reflect the results of the experiments in an accurate and unbiased manner.
- ideally, figure panels should include only measurements that are directly comparable to each other and obtained with the same assay.
- plots include clearly labeled error bars for independent experiments and sample sizes. Unless justified, error bars should not be shown for technical
- if n<5, the individual data points from each experiment should be plotted. Any statistical test employed should be justified.
- Source Data should be included to report the data underlying figures according to the guidelines set out in the authorship guidelines on Data

2. Captions
Each figure caption should contain the following information, for each panel where they are relevant:
- a specification of the experimental system investigated (eg cell line, species name).
- the assay(s) and method(s) used to carry out the reported observations and measurements.
- an explicit mention of the biological and chemical entity(ies) that are being measured.
- an explicit mention of the biological and chemical entity(ies) that are altered/varied/perturbed in a controlled manner.
- the exact sample size (n) for each experimental group/condition, given as a number, not a range;
- a description of the sample collection allowing the reader to understand whether the samples represent technical or biological replicates (including how many animals, litters, cultures, etc.).
- a statement of how many times the experiment shown was independently replicated in the laboratory.
- definitions of statistical methods and measures:
  - common tests, such as t-test (please specify whether paired vs. unpaired), simple χ2 tests, Wilcoxon and Mann-Whitney tests, can be unambiguously identified by name only, but more complex techniques should be described in the methods section;
  - are tests one-sided or two-sided?
  - are there adjustments for multiple comparisons?
  - exact statistical test results, e.g., P values = x but not P values < x;
  - definition of 'center values' as median or average;
  - definition of error bars as s.d. or s.e.m.

Please complete ALL of the questions below.
Select "Not Applicable" only when the requested information is not relevant for your study.

Materials

Newly Created Materials	Information included in the manuscript?	In which section is the information available? (Reagents and Tools Table, Materials and Methods, Figures, Data Availability Section)
New materials and reagents need to be available; do any restrictions apply?	Not Applicable	

Antibodies	Information included in the manuscript?	In which section is the information available? (Reagents and Tools Table, Materials and Methods, Figures, Data Availability Section)
For **antibodies** provide the following information:<br>- Commercial antibodies: RRID (if possible) or supplier name, catalogue number and or/clone number<br>- Non-commercial: RRID or citation	Yes	Materials and Methods

DNA and RNA sequences	Information included in the manuscript?	In which section is the information available? (Reagents and Tools Table, Materials and Methods, Figures, Data Availability Section)
**Short novel DNA or RNA including primers, probes**: provide the sequences.	Yes	Supplementary Material

Cell materials	Information included in the manuscript?	In which section is the information available? (Reagents and Tools Table, Materials and Methods, Figures, Data Availability Section)
**Cell lines:** Provide species information, strain. Provide accession number in repository **OR** supplier name, catalog number, clone number, and/**OR** RRID.	Yes	Materials and Methods
**Primary cultures:** Provide species, strain, sex of origin, genetic modification status.	Not Applicable	
Report if the cell lines were recently **authenticated** (e.g., by STR profiling) and tested for mycoplasma contamination.	Yes	Materials and Methods

Experimental animals	Information included in the manuscript?	In which section is the information available? (Reagents and Tools Table, Materials and Methods, Figures, Data Availability Section)
**Laboratory animals or Model organisms:** Provide species, strain, sex, age, genetic modification status. Provide accession number in repository **OR** supplier name, catalog number, clone number, **OR** RRID.	Yes	Materials and Methods
**Animal observed in or captured from the field:** Provide species, sex, and age where possible.	Not Applicable	
Please detail **housing and husbandry conditions**.	Yes	Materials and Methods

Plants and microbes	Information included in the manuscript?	In which section is the information available? (Reagents and Tools Table, Materials and Methods, Figures, Data Availability Section)
**Plants:** provide species and strain, ecotype and cultivar where relevant, unique accession number if available, and source (including location for collected wild specimens).	Not Applicable	
**Microbes:** provide species and strain, unique accession number if available, and source.	Not Applicable	

Human research participants	Information included in the manuscript?	In which section is the information available? (Reagents and Tools Table, Materials and Methods, Figures, Data Availability Section)
If collected and within the bounds of privacy constraints report on age, sex and gender or ethnicity for all study participants.	Not Applicable	

Core facilities	Information included in the manuscript?	In which section is the information available? (Reagents and Tools Table, Materials and Methods, Figures, Data Availability Section)
If your work benefited from core facilities, was their service mentioned in the acknowledgments section?	Yes	Acknowlegments

Design

Study protocol	Information included in the manuscript?	In which section is the information available? (Reagents and Tools Table, Materials and Methods, Figures, Data Availability Section)
If study protocol has been **pre-registered, provide DOI in the manuscript**. For clinical trials, provide the trial registration number **OR** cite DOI.	Not Applicable	
Report the **clinical trial registration number** (at ClinicalTrials.gov or equivalent), where applicable.	Not Applicable	

Laboratory protocol	Information included in the manuscript?	In which section is the information available? (Reagents and Tools Table, Materials and Methods, Figures, Data Availability Section)
Provide DOI OR other citation details if **external detailed step-by-step protocols** are available.	Not Applicable	

Experimental study design and statistics	Information included in the manuscript?	In which section is the information available? (Reagents and Tools Table, Materials and Methods, Figures, Data Availability Section)
Include a statement about **sample size** estimate even if no statistical methods were used.	Yes	Figures
Were any steps taken to minimize the effects of subjective bias when allocating animals/samples to treatment (e.g. **randomization procedure**)? If yes, have they been described?	Yes	Materials and Methods
Include a statement about **blinding** even if no blinding was done.	Yes	Materials and Methods
Describe **inclusion/exclusion criteria** if samples or animals were excluded from the analysis. Were the criteria pre-established?<br><br>If sample or data points were omitted from analysis, report if this was due to attrition or intentional exclusion and provide justification.	Yes	Materials and Methods
For every figure, are **statistical tests** justified as appropriate? Do the data meet the assumptions of the tests (e.g., normal distribution)? Describe any methods used to assess it. Is there an estimate of variation within each group of data? Is the variance similar between the groups that are being statistically compared?	Yes	Figures

Sample definition and in-laboratory replication	Information included in the manuscript?	In which section is the information available? (Reagents and Tools Table, Materials and Methods, Figures, Data Availability Section)
In the figure legends: state number of times the experiment was **replicated** in laboratory.	Yes	Figures
In the figure legends: define whether data describe **technical or biological replicates**.	Yes	Figures

Ethics

Ethics	Information included in the manuscript?	In which section is the information available? (Reagents and Tools Table, Materials and Methods, Figures, Data Availability Section)
Studies involving **human participants**: State details of **authority granting ethics approval** (IRB or equivalent committee(s), provide reference number for approval.	Not Applicable	
Studies involving **human participants**: Include a statement confirming that **informed consent** was obtained from all subjects and that the experiments conformed to the principles set out in the WMA Declaration of Helsinki and the Department of Health and Human Services Belmont Report.	Not Applicable	
Studies involving **human participants: For publication of patient photos**, include a statement confirming that consent to publish was obtained.	Not Applicable	
Studies involving experimental **animals**: State details of **authority granting ethics approval** (IRB or equivalent committee(s), provide reference number for approval. Include a statement of compliance with ethical regulations.	Yes	Materials and Methods
Studies involving **specimen and field samples:** State if relevant **permits** obtained, provide details of authority approving study; if none were required, explain why.	Not Applicable	

Dual Use Research of Concern (DURC)	Information included in the manuscript?	In which section is the information available? (Reagents and Tools Table, Materials and Methods, Figures, Data Availability Section)
Could your study fall under dual use research restrictions? Please check biosecurity documents and list of **select agents and toxins** (CDC): https://www.selectagents.gov/sat/list.htm	Not Applicable	
If you used a select agent, is the security level of the lab appropriate and reported in the manuscript?	Not Applicable	
If a study is subject to dual use research of concern regulations, is the name of the **authority granting approval and reference number** for the regulatory approval provided in the manuscript?	Not Applicable	

Reporting

The MDAR framework recommends adoption of discipline-specific guidelines, established and endorsed through community initiatives. Journals have their own policy about requiring specific guidelines and recommendations to complement MDAR.

Adherence to community standards	Information included in the manuscript?	In which section is the information available? (Reagents and Tools Table, Materials and Methods, Figures, Data Availability Section)
State if relevant guidelines or checklists (e.g., **ICMJE, MIBBI, ARRIVE, PRISMA**) have been followed or provided.	Yes	Materials and Methods
For **tumor marker prognostic studies,** we recommend that you follow the **REMARK** reporting guidelines (see link list at top right). See author guidelines, under 'Reporting Guidelines'. Please confirm you have followed these guidelines.	Not Applicable	
For **phase II and III randomized controlled trials**, please refer to the **CONSORT** flow diagram (see link list at top right) and submit the CONSORT checklist (see link list at top right) with your submission. See author guidelines, under 'Reporting Guidelines'. Please confirm you have submitted this list.	Not Applicable	

Data Availability

Data availability	Information included in the manuscript?	In which section is the information available? (Reagents and Tools Table, Materials and Methods, Figures, Data Availability Section)
Have **primary datasets** been deposited according to the journal's guidelines (see 'Data Deposition' section) and the respective accession numbers provided in the Data Availability Section?	Yes	Materials and Methods
Were **human clinical and genomic datasets** deposited in a public access-controlled repository in accordance to ethical obligations to the patients and to the applicable consent agreement?	Not Applicable	
Are **computational models** that are central and integral to a study available without restrictions in a machine-readable form? Were the relevant accession numbers or links provided?	Not Applicable	
If publicly available data were reused, provide the respective **data citations in the reference list**.	Not Applicable	
